# Supplementary material for: Biobank of genetically defined murine prostate cancer tumoroids uncovers oncogenic pathways and drug vulnerabilities driven by PTEN-loss
Source: Cell Rep Methods. 2026 Mar 30;6(4):101370. doi: 10.1016/j.crmeth.2026.101370 (PMC13106976; doi:10.1016/j.crmeth.2026.101370)
Supplement: Document S1. Figures S1–S7 and Tables S1 and S2 [file mmc1.pdf]

Cell Reports Methods, Volume *6*

Supplemental information

Biobank of genetically defined murine prostate cancer tumoroids uncovers oncogenic pathways and drug vulnerabilities driven by PTEN-loss

Jessica Kalla, Thomas Dillinger, Zlata Pavlovicova, Reema Jacob, Emine Atas, Katarina Mišura, Anil Baskan, Kristina Draganić, Andreas Tiefenbacher, Tanja Limberger, Theresia Mair, Gabriel Wasinger, Ludovica Villanti, Stefan Kubicek, Lukas Kenner, and Gerda Egger

SUPPLEMENTARY FIGURES AND FIGURE LEGENDS

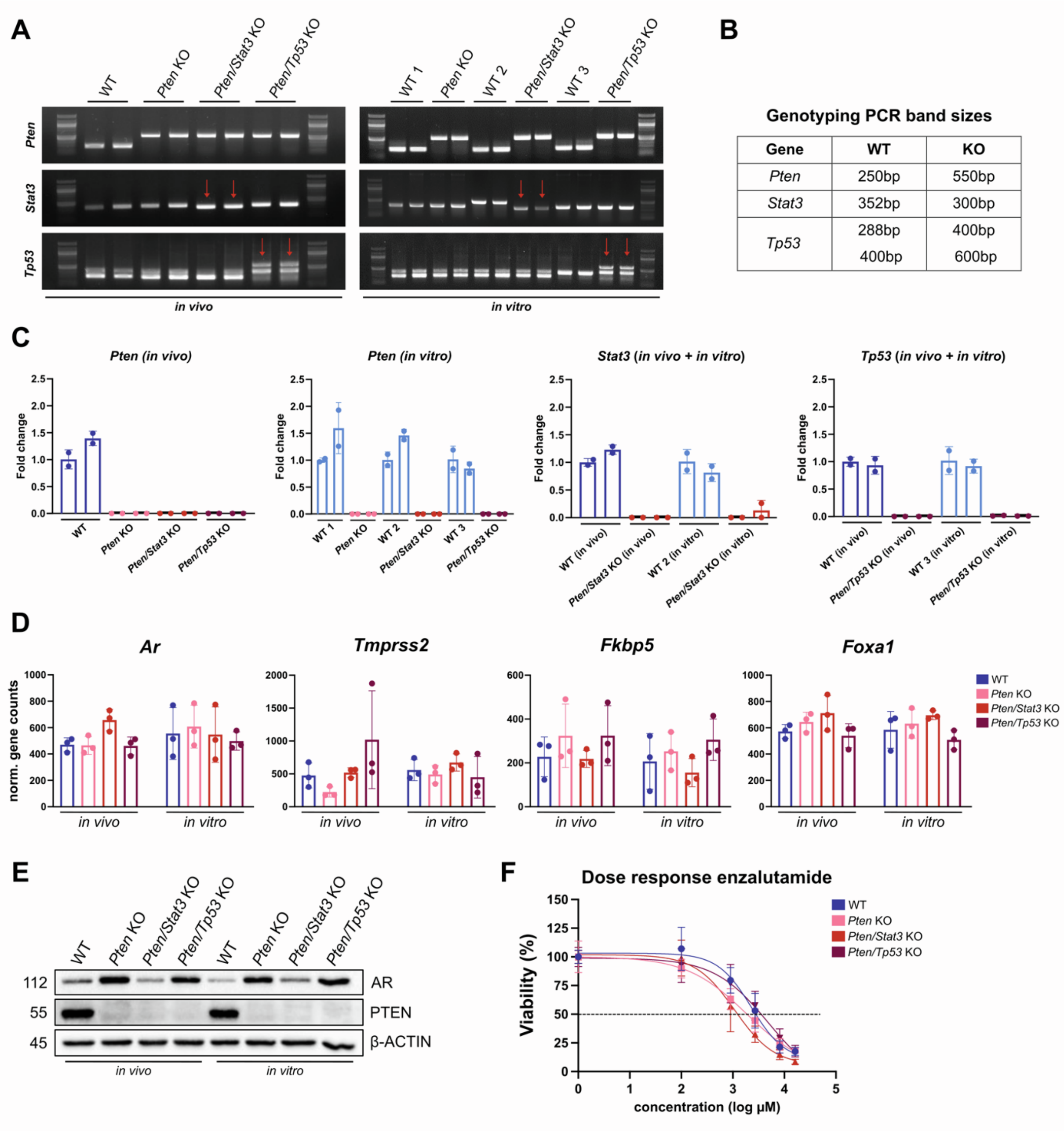

Gene	WT	KO
Pten	250bp	550bp
Stat3	352bp	300bp
Tp53	288bp<br>400bp	400bp<br>600bp

Figure S1. Confirmation of deletion of genes of interest on DNA and RNA level, and androgen status of organoid/tumoroid models, Related to Figure 1.

(A) Representative images of gel electrophoresis for genotyping using genomic DNA of *in vivo* (left) and *in vitro* (right) organoids and tumoroids for *Pten* (top), *Stat3* (middle), and *Tp53* (bottom). Bands were visualised using UV light. Arrows in middle and lower panels point to KO-specific bands (N=2). **(B)** Table summarising band sizes shown in (A) for WT or KO sequences for *Pten*, *Stat3*, and *Tp53*. **(C)** Representative gene expression analysis for *Pten*, *Stat3*, and *Tp53* of indicated organoid and tumoroid lines quantified by qRT-PCR. Expression levels are shown relative to *β-Actin* as housekeeping gene and normalised to one WT organoid line. Bars represent the mean ± SD of technical duplicates. **(D)** Bar graph showing normalised counts of androgen receptor (*Ar*), AR target genes *Tmprss2* and *Fkbp5*, and AR co-factor *Foxa1* across different models and genotypes. Bars represent the mean ± SD of biological triplicates. Normalisation and transformation were performed using DESeq2 to account for library size differences. **(E)** Western blot analysis of representative murine *in vivo* and *in vitro* organoids and KO tumoroids for indicated genotypes for AR, PTEN, and β-ACTIN as loading control. **(F)** Dose-response curves for enzalutamide on *in vivo* WT organoids and KO tumoroids (WT: 22.86μM, *Pten* KO: 18.28μM, *Pten/Stat3* KO: 10.86μM, *Pten/Tp53* KO: 48.19μM). Points represent means and ± SD of technical triplicates (N=3). Curve fitting was performed using GraphPad Prism 8.0.2.

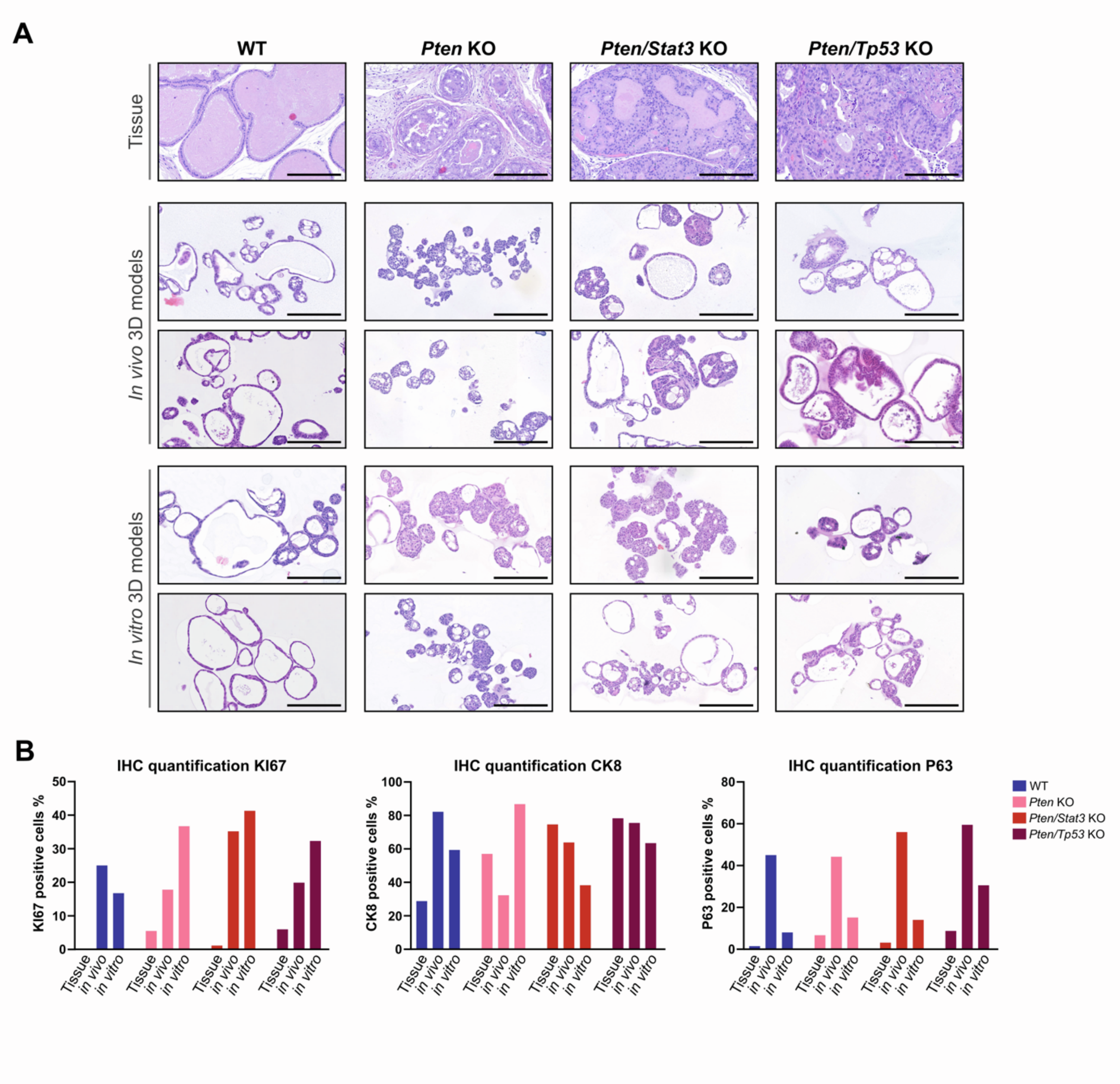

Figure S2. Morphology and marker expression with quantification of tissue and organoid/tumoroids lines, Related to Figure 2.

(A) Representative images of murine WT and tumour tissues and *in vivo* and *in vitro* organoid and tumoroid models stained with haematoxylin and eosin (HE). Stainings show biological replicates for tissues and organoids/tumoroids in addition to samples shown in Figure 2. Scale bar 200µm. (**B)** Quantification of protein expression of immunohistochemistry stainings from Figure 2 for KI67, CK8, and P63. Bars represent percentages of positive cells (nucleus or cytoplasm) for each representative tissue/organoid/tumoroid. Quantification was performed in QuPath 0.4.4 and data was visualised with GraphPad Prism 8.0.2.

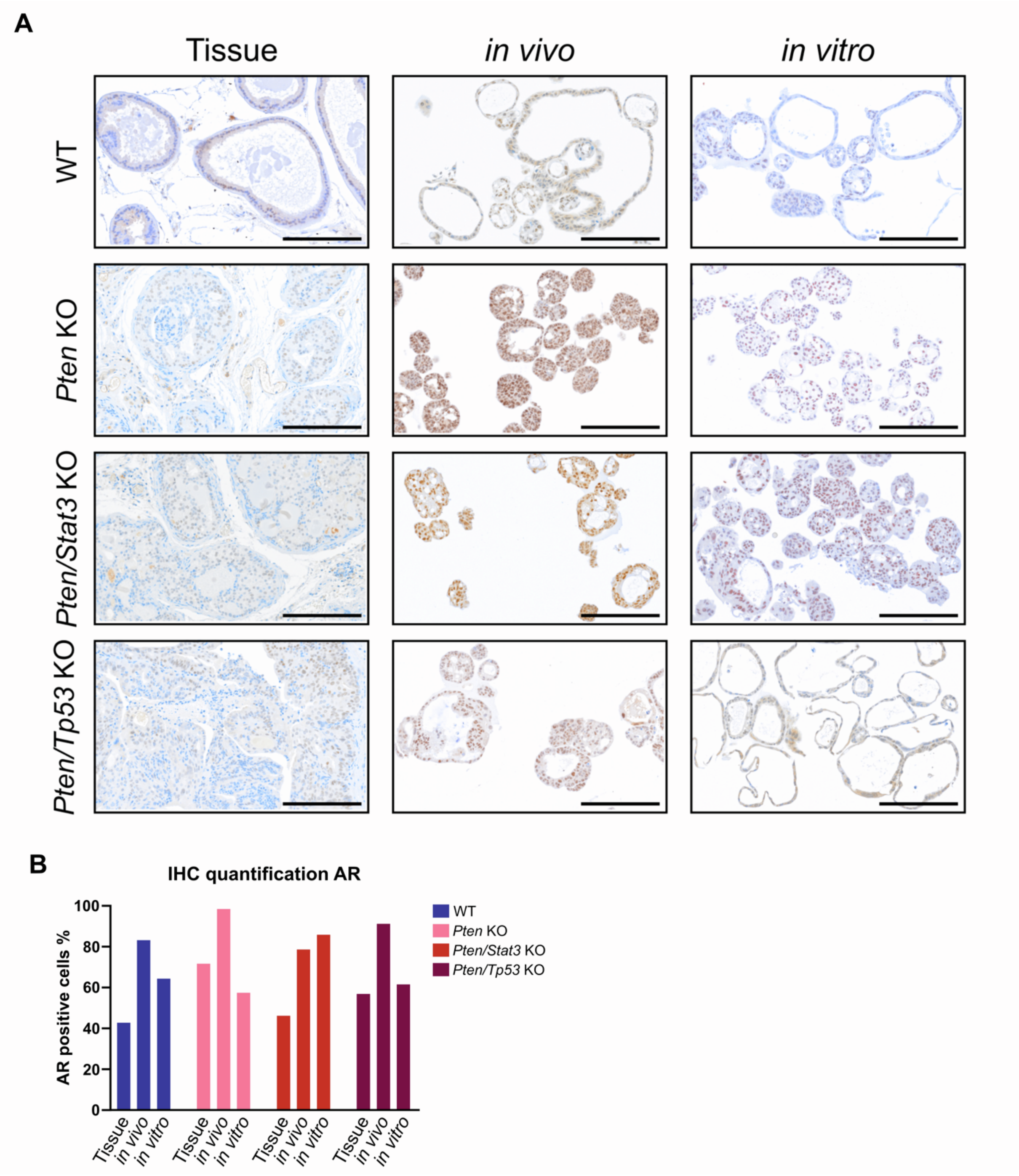

Figure S3. AR expression with quantification of tissue and organoid/tumoroids lines, Related to Figure 2.

(A) Immunohistological comparison between representative prostate tissues/tumours and organoids/tumoroids of all genotypes stained for androgen receptor (AR). Same samples as in Figure S1D-E are shown. Scale bar 200μM. **(B)** Quantification of nuclear AR expression shown in A. performed as described in B.

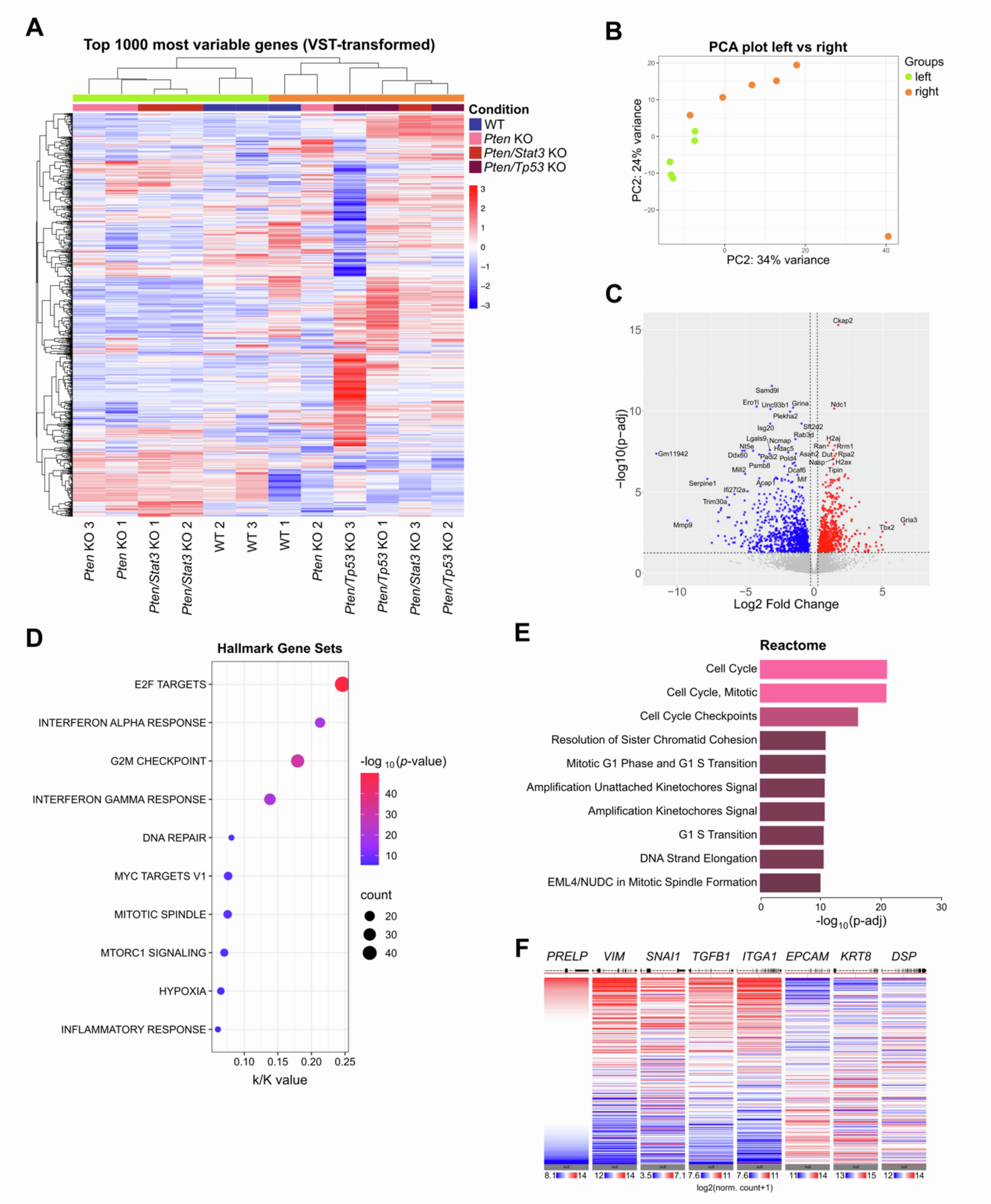

Figure S4. Organoid and tumoroid lines show different transcriptomes based on cell cycle regulation, Related to Figure 3.

(A) Dendrogram and heatmap showing unsupervised hierarchical clustering of the top 1000 most variable genes for all *in vivo* organoid and tumoroid lines based on VST-normalised gene counts as shown in Figure 3D, colour-coded for the two main branches of the dendrogram in green (left) and orange (right). (**B)** Principal component analysis (PCA) of bulk RNA sequencing data of *in vivo* WT organoid and indicated KO tumoroid lines (N=3). Samples are grouped based on clustering in the heatmap in (A). (**C)** Volcano plot depicting differentially expressed genes (DEGs) for *in vivo* WT organoid and KO tumoroid lines grouped as in (A,B). Genes with p-adj < 0.05 and Log2fold > 0 (red) or < 0 (blue) are highlighted. (**D)** Bubble plot showing the top 10 enriched hallmark gene sets identified by MSigDB gene set enrichment analysis of DEGs as in (C). Size of points reflects number of DEGs mapped to specific pathways, while colour reflects statistical significance (-log10 *p*-value). k/K value describes the

ratio of number of genes in input list (k) divided by the number of total genes in the gene set of the database (K). (**E)** Bar graph showing the top 10 enriched Reactome pathways (Enrichr) of DEGs as in (C) sorted by statistical significance (-log10 p-adj). (**F)** XENA browser analysis[1] showing gene expression analysis based on the TCGA-PRAD RNA sequencing data set[2] for *Prelp* and its correlation with mesenchymal genes *VIM*, *SNAI1*, *TGFβ1*, and *ITGA1*, and negative correlation with epithelial genes *EPCAM*, *KRT8*, and *DSP*. Each line represents one patient with gene expression indicated as log2 (norm. count +1, red: upregulation, blue: downregulation).

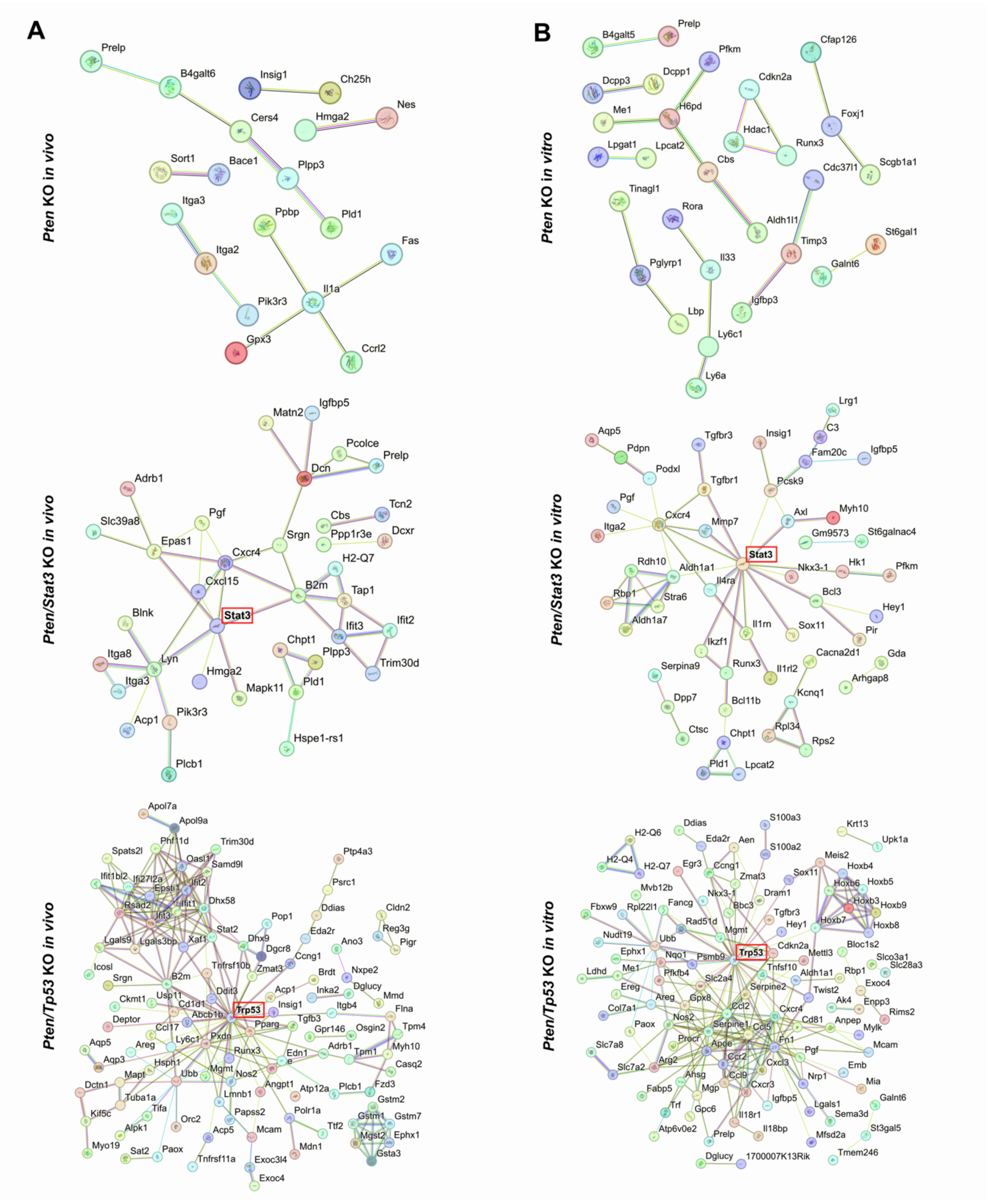

Figure S5. Differentially expressed genes extracted from in vivo and in vitro tumoroid vs organoid comparisons form functional clusters, Related to Figure 4.

Protein-protein interaction networks based on significant differentially expressed genes (DEGs) for *in vivo* (**A**) and *in vitro* (**B**) *Pten* KO (top), *Pten/Stat3* KO (middle), and *Pten/Tp53* KO (bottom) tumoroids compared to WT organoids constructed using the STRING database. For *Pten* KO and *Pten/Stat3* KO tumoroids all DEGs were used, while for *Pten/Tp53* KO tumoroids the top 200 DEGs were used. *Stat3* and *Tp53* are highlighted in red.

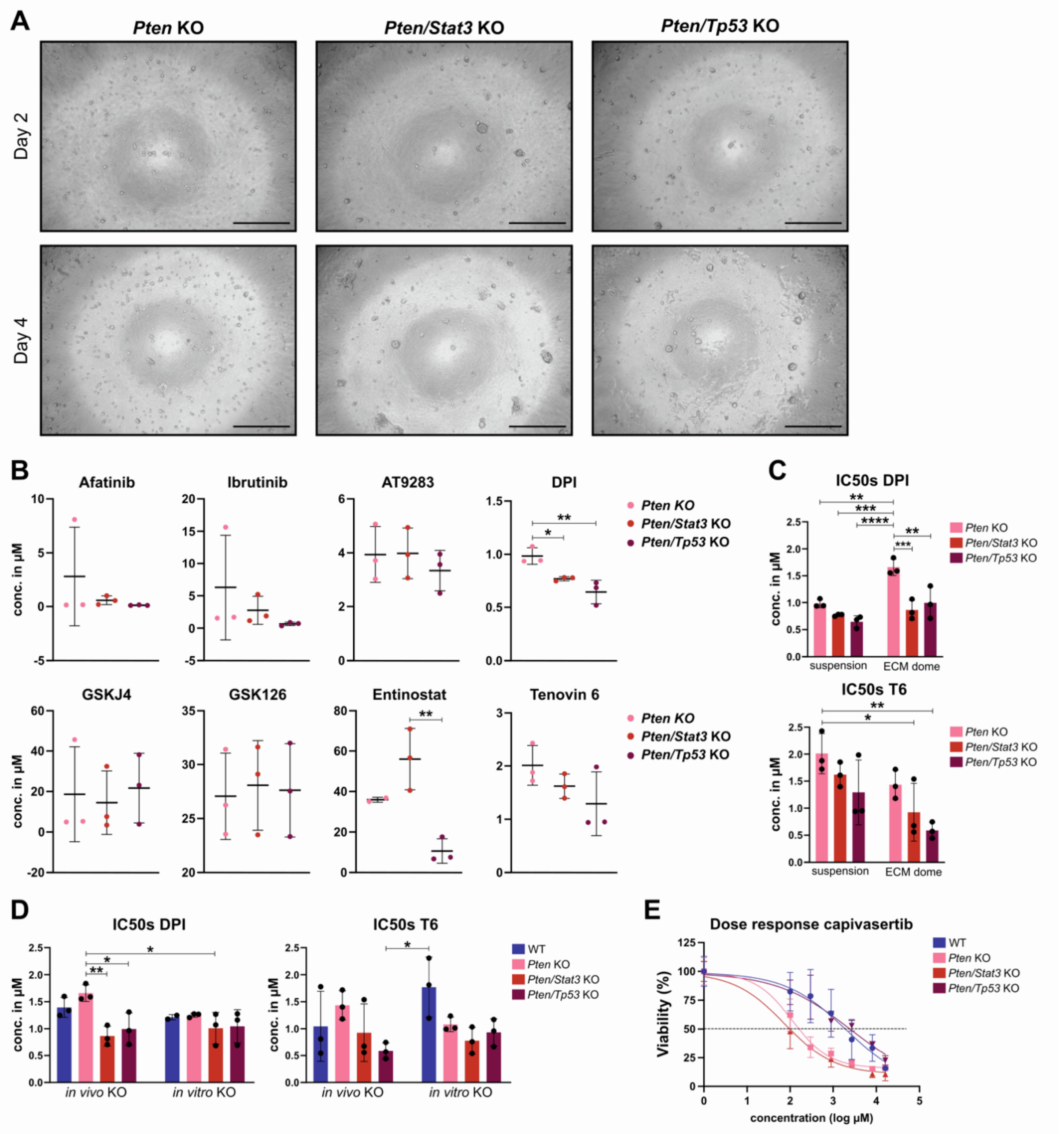

Figure S6. Dose-response of selected compounds and survival analysis of PDPK1/AKT/FLT dual pathway inhibitor (DPI) and tenovin-6 (T6) targets, Related to Figure 6.

(A) Representative bright-field microscopic images of untreated control tumoroids seeded in 384-well plates for dose-response screens. Day 2 corresponds to the first day under drug treatment, after tumoroids were seeded as single cells on Day 0. Scalebar 500µM. (**B)** Dot plots showing the mean ± SD of the half-maximal inhibitory concentrations (IC50) for eight compounds selected from the medium-throughput drug screen (related to Fig. 6E) screened in suspension (N=3). Statistical analysis was performed using GraphPad Prism 8.0.2 (One-way ANOVA, Tukey´s test). $p > 0.05$ if not specified otherwise, *$p \leq 0.05$; **$p \leq 0.01$. **(C)** Bar graphs showing means and ± SD of IC50 values for the DPI (top) and T6 (bottom) either measured in suspension or in extracellular matrix (ECM) domes for all *in vivo* KO tumoroids (N=3). Statistical analysis was performed using GraphPad Prism 8.0.2 (One-way ANOVA, Tukey´s test). $p > 0.05$ if not specified otherwise, *$p \leq 0.05$, **$p \leq 0.01$, ***$p \leq 0.001$, ****$p \leq 0.0001$. **(D)** Bar graphs showing means and ± SD of IC50 as shown in Figure 7D for DPI (left) and T6 (right) for *in vivo* and *in vitro* WT organoid and tumoroid lines of all genotypes (N=3). Statistical analysis was performed using GraphPad Prism 8.0.2 (One-way ANOVA, Tukey´s test). $p > 0.05$ if not specified otherwise, *$p \leq 0.05$. **(E)** Dose-response curves for capivasertib on *in vivo* WT organoids and KO tumoroids (WT: 19.82µM, *Pten* KO: 1.12µM, *Pten/Stat3* KO: 0.74µM, *Pten/Tp53* KO: 25.59µM). Points represent means and ± SD of technical triplicates (N=3). Curve fitting was performed using GraphPad Prism 8.0.2.

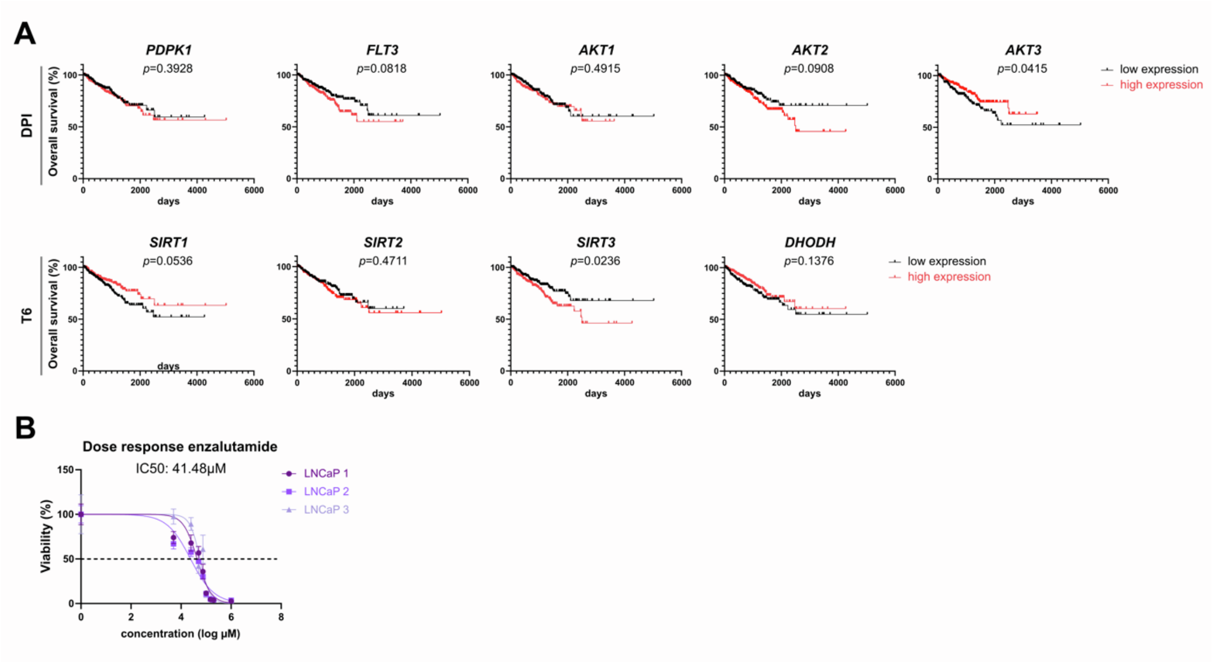

Figure S7. Kaplan-Meier analysis of DPI and T6 targets on publicly available human PCa data and response of LNCaP cells to enzalutamide, Related to Figure 7.

(A) Kaplan-Meier survival analysis based on TCGA-PRAD RNA sequencing data for DPI targets *PDPK1*, *AKT1*, *AKT2*, *AKT3*, and *FLT3* (top) and T6 targets *SIRT1*, *SIRT2*, *SIRT3*, and *DHODH* (bottom).[3] Statistical analysis was done using GraphPad Prism 8.0.2 (Mantel-Cox test). **(B)** Dose-response curves for enzalutamide on the human PCa cell line LNCaP. Points represent means and ± SD of technical triplicates (N=3). Curve fitting was performed using GraphPad Prism 8.0.2.

SUPPLEMENTARY TABLES

Table S1. PCR primer sequences, Related to STAR Methods.

**Genotyping primer**	**Sequence**
Cre fw	ATGCTTCTGTCCGTTTGCCG
Cre rev	TGAGTGAACGAACCTGGTCG
CreCut fw	CGACCAGGTTCGTTCACTCA
CreCut rev	GGTTGGCAGCTCTCATGTCT
Pten fw	TTCAGTTGTCTTTTACATTTCCTTTG
Pten rev 1	GTCAGAAACGGCCTTAACGA
Pten rev 2	TCAGGTAAGGGGTACTGATTTTT
Stat3 fw	GCGTCTGACTCTACA ACC
Stat3 rev 1	AGCCTCATCCTTAGGTACT
Stat3 rev 2	GACTGTGATAACCTTCAGTG
Tp53 fw	CACAAAAACAGGTTAAACCCAG
Tp53 rev 1	AGCACATAGGAGGCAGAGAC
Tp53 rev 2	GAAGACAGAAAAGGGGAGGG
**qRT-PCR primer**	**Sequence**
Pten fw	GACATTATGACACCGCCAAA
Pten rev	CAGTGAATTGCTGCAACATGA
Stat3 fw	AGACTCTGGGGATGTTGCTG
Stat3 rev	ACAGGCTGCCGTTGTTAGAC
Tp53 fw	CCATGGCCCCTGTCATCTTT
Tp53 rev	TGACCCACAACTGCACAGG
Ar fw	ATTGAGCCAGGAGTGGTGTG
Ar rev	ACCATCAGTCCCATCCAGGA
β-Actin fw	ATGCCCTGAGGCTCTTTTCC
β-Actin rev	AATGCCTGGGTACATGGTGG

Table S2. Antibody dilutions, Related to STAR Methods

**Target**	**Dilution**
β-ACTIN	WB 1:5000
AKT (pan)	WB 1:1000
CK8	IHC 1:200
KI67	IHC 1:400
P63	IHC 1:80
pospho-AKT	WB 1:1000
PTEN	WB 1:1000
STAT3	WB 1:1000
TP53	WB 1:500
AR (Abcam)	WB 1:1000<br>IHC 1:120
AR (Cell Signaling)	IHC 1:120

SUPPLEMENTARY REFERENCES

1. Goldman, M.J., Craft, B., Hastie, M., Repečka, K., McDade, F., Kamath, A., Banerjee, A., Luo, Y., Rogers, D., Brooks, A.N., et al. (2020). Visualizing and interpreting cancer genomics data via the Xena platform. Nat Biotechnol *38*, 675–678. https://doi.org/10.1038/s41587-020-0546-8.

2. Abeshouse, A., Ahn, J., Akbani, R., Ally, A., Amin, S., Andry, C.D., Annala, M., Aprikian, A., Armenia, J., Arora, A., et al. (2015). The Molecular Taxonomy of Primary Prostate Cancer. Cell *163*, 1011–1025. https://doi.org/10.1016/j.cell.2015.10.025.

3. Smith, J.C., and Sheltzer, J.M. (2022). Genome-wide identification and analysis of prognostic features in human cancers. Cell Reports *38*, 110569. https://doi.org/10.1016/j.celrep.2022.110569.
