# Supplementary material for: Biobank of genetically defined murine prostate cancer tumoroids uncovers oncogenic pathways and drug vulnerabilities driven by PTEN-loss
Source: Cell Rep Methods. 2026 Mar 30;6(4):101370. doi: 10.1016/j.crmeth.2026.101370 (PMC13106976; doi:10.1016/j.crmeth.2026.101370)
Supplement: Document S2. Article plus supplemental information [file mmc4.pdf]

Cell Reports
Methods

Resource

Biobank of genetically defined murine prostate cancer tumoroids uncovers oncogenic pathways and drug vulnerabilities driven by PTEN-loss

Graphical abstract

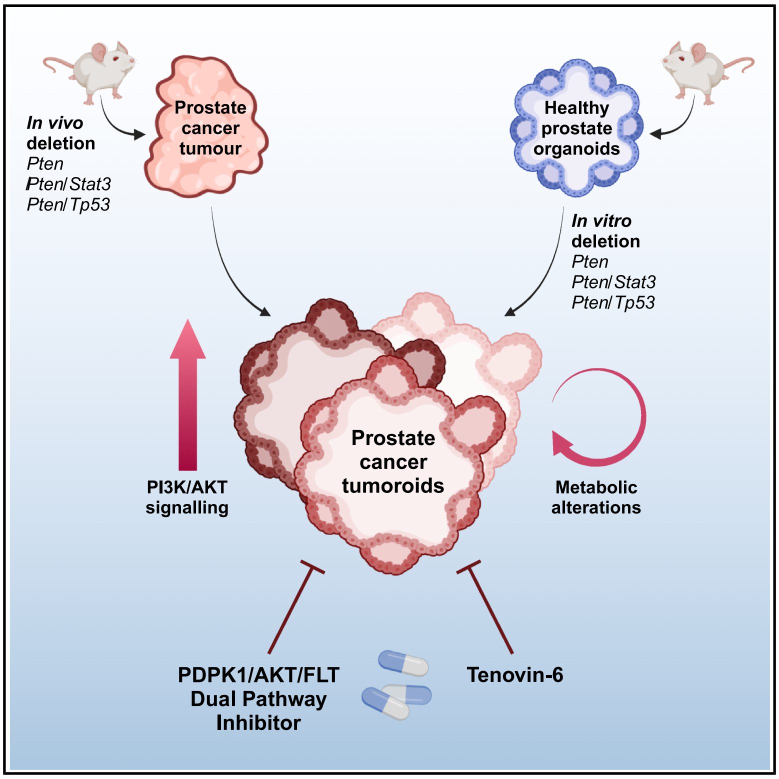

Authors

Jessica Kalla, Thomas Dillinger, Zlata Pavlovicova, ..., Stefan Kubicek, Lukas Kenner, Gerda Egger

Correspondence

gerda.egger@meduniwien.ac.at

In brief

Kalla et al. establish and characterize a biobank of murine prostate organoids and prostate cancer tumoroids that model key patient-relevant mutations, providing a versatile platform for drug discovery. A medium-throughput drug screen identifies targeted compounds that effectively inhibit prostate cancer growth and enhance antiandrogen therapy for future treatment development.

Highlights

- We establish a murine organoid/tumoroid biobank for PCa research
- Unique metabolic characteristics of PCa are dependent on the PI3K/AKT pathway
- A medium-throughput drug screen identifies PCa-specific compounds
- Targeting PI3K/AKT signaling or sirtuins are promising treatment options for PCa

Kalla et al., 2026, Cell Reports Methods *6*, 101370
April 20, 2026 © 2026 The Authors. Published by Elsevier Inc.
https://doi.org/10.1016/j.crmeth.2026.101370

CellPress

Cell Reports Methods

CellPress
OPEN ACCESS

Resource

Biobank of genetically defined murine prostate cancer tumoroids uncovers oncogenic pathways and drug vulnerabilities driven by PTEN-loss

Jessica Kalla,[1] Thomas Dillinger,[2] Zlata Pavlovicova,[2] Reema Jacob,[2] Emine Atas,[1,3] Katarina Mišura,[1] Anil Baskan,[1] Kristina Draganić,[1] Andreas Tiefenbacher,[1] Tanja Limberger,[1,4] Theresia Mair,[1] Gabriel Wasinger,[1] Ludovica Villanti,[5] Stefan Kubicek,[5] Lukas Kenner,[1,3,4,6,7,8] and Gerda Egger[1,2,7,9,*]

[1]Department of Pathology, Medical University of Vienna, Vienna, Vienna 1090, Austria
[2]Ludwig Boltzmann Institute Applied Diagnostics, Vienna, Vienna 1090, Austria
[3]Christian Doppler Laboratory for Applied Metabolomics, Medical University of Vienna, Vienna, Vienna 1090, Austria
[4]Centre for Biomarker Research in Medicine GmbH (CBmed), Graz, Styria 8010, Austria
[5]CeMM Research Center for Molecular Medicine of the Austrian Academy of Sciences, Vienna, Vienna 1090, Austria
[6]Unit of Laboratory Animal Pathology, University of Veterinary Medicine Vienna, Vienna, Vienna 1210, Austria
[7]Comprehensive Cancer Center, Medical University of Vienna, Vienna, Vienna 1090, Austria
[8]Department of Molecular Biology, Umeå University, 901 87 Umeå, Västerbottens, Sweden
[9]Lead contact
*Correspondence: gerda.egger@meduniwien.ac.at
https://doi.org/10.1016/j.crmeth.2026.101370

MOTIVATION Patient-derived organoid and tumoroid models have proven invaluable for cancer research, enhancing our understanding of tumorigenesis and enabling the testing of therapeutics for personalized treatment. In case of prostate cancer, long-term cultivation of primary tumors has been less successful and thus fewer models are available. Therefore, we developed organoid and tumoroid models originating from murine prostate tissues and tumors of transgenic mice, harboring selected patient-relevant mutations, to study genotype-specific drug responses. These models are readily genetically manipulated *in vitro* and can be cultured for extended passages.

SUMMARY

Prostate cancer (PCa) is the second most common cancer in men and shows high inter- and intra-patient heterogeneity. Consequently, treatment options are limited and there is a lack of representative preclinical models. Here, we establish a comprehensive biobank of murine organoids and tumoroids that reflect common patient mutations. We demonstrate that the deletion of *Pten* alone, or in combination with *Stat3*, or *Tp53*, drives the activation of cancer-related pathways in both prostate organoids and tumor-derived tumoroids. A medium-throughput drug screen identified two potent compounds, the PDPK1/AKT/FLT dual pathway inhibitor and the sirtuin inhibitor tenovin-6, which effectively suppressed tumoroid proliferation. Notably, these compounds also inhibited the growth of several human PCa cell lines and displayed synergistic effects when combined with the standard-of-care antiandrogen enzalutamide. Together, our findings provide evidence that murine tumoroids are versatile preclinical models for studying PCa tumorigenesis and drug sensitivities to develop therapeutic options for PCa patients.

INTRODUCTION

Prostate cancer (PCa), the second leading cause of cancer-related death in men worldwide,[1] is characterized by a diverse mutational landscape and high inter- and intra-patient heterogeneity.[2–4] The malignant transformation of the normal prostate gland, which consists of luminal and basal epithelial cells, to PCa is a multifactorial process. Different driver events lead to the development of adenocarcinoma lesions that ultimately progress to metastatic disease.[5,6] Radical prostatectomy, radiation therapy, and subsequent androgen deprivation therapy represent the primary treatment options for localized disease.[7] However, despite initial response to these therapies, many patients eventually develop castration-resistant PCa and metastases, posing a significant therapeutic challenge due to limited treatment options and poor prognosis.[8]

© 2026 The Authors. Published by Elsevier Inc.
This is an open access article under the CC BY license (http://creativecommons.org/licenses/by/4.0/).

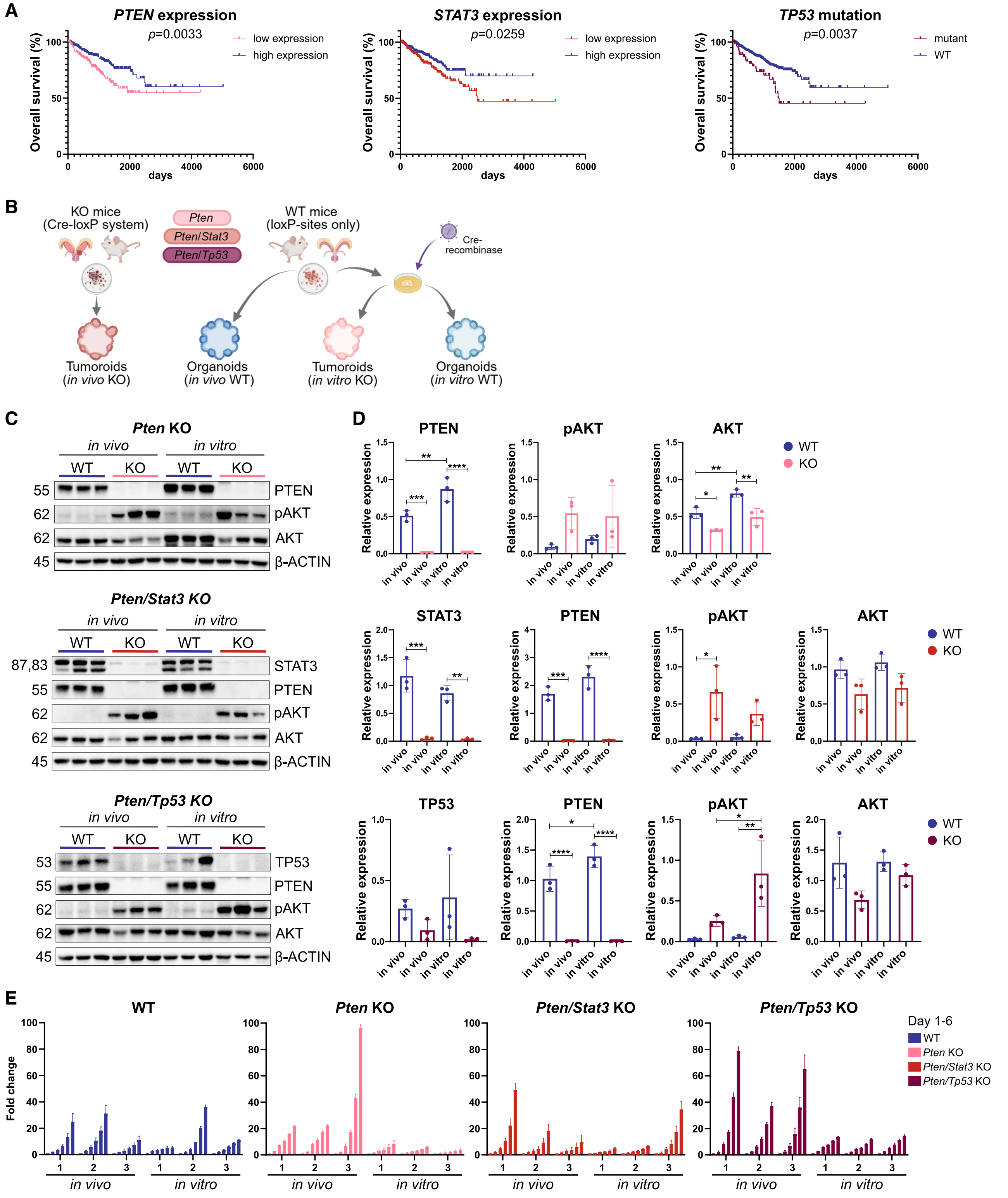

Figure 1. Establishment and genetic stability of murine PCa tumoroids reflecting patient mutations

(A) Kaplan-Meier survival analysis of human PCa patients based on TCGA-PRAD RNA sequencing data for expression of *PTEN* (left), and *STAT3* (middle), or whole-genome/exome sequencing for *TP53* mutation status (right).[39] Statistical analysis was done using GraphPad Prism 8.0.2 (Mantel-Cox test).

(legend continued on next page)

Even though PCa is a very heterogeneous disease, some mutational patterns can be found in a large subgroup of patients.[9] The loss of the tumor suppressor *PTEN* and thus an activation of the PI3K/AKT pathway, is one of the most common mutations found in PCa with an incidence of ~20% in primary cases, and ~50% in metastatic disease.[10] *TP53* is also commonly mutated in PCa patients, and mutations in *TP53* frequently occur in combination with *PTEN* deletions.[9,11] As *STAT3* is upregulated in many cancer types including PCa,[12,13] the inhibition of the IL-6/STAT3 signaling axis has been reported as a therapy approach for PCa.[14] However, in mice the loss of *Stat3* in combination with the loss of *Pten*, a co-deletion observed in 66% of patients, led to a more aggressive and invasive phenotype, highlighting the dual role of STAT3 for PCa.[15–17]

Despite significant advances in our understanding of this disease, the development of effective therapies has been hindered by the lack of robust preclinical models that recapitulate the complex biology and treatment response of PCa patients.[18] Until now, mainly 2D cell lines including 22RV1 cells (primary tumor), the metastatic cell lines LNCaP (lymph node), DU145 (brain), and PC3 (bone), or non-cancerous cell lines including RWPE-1 (normal) and BPH-1 (benign hyperplastic) have been among the most widely used human models for PCa research.[18,19] Since these cell lines consist of only one cell type, they do not fully recapitulate the *in vivo* tissue function and signaling of PCa tumors.[20] Organoids derived from stem cells of healthy tissues, and tumoroids derived from malignant lesions, are 3D *in vitro* models generated from primary patient tissue or animal samples, that have emerged as a promising platform for cancer research.[21] Even though it is possible to generate human prostate organoids and PCa tumoroids from tissue biopsies but also from induced pluripotent stem cells,[22–26] these models only reflect a small subset of PCa and long-term cultivation for extended passages is limited. Primary PCa tumoroids get overgrown by healthy cells and most 3D models stop proliferating due to suboptimal medium and matrix conditions.[25,27–30] Thus, murine organoids and tumoroids, which can be maintained *in vitro* indefinitely, provide a versatile tool for PCa research to study PCa tumorigenesis and therapy response.[23,25,31–33]

As the influence of different genetic mutations of murine prostate organoid and PCa tumoroid models on gene expression and drug response has not been studied extensively, we focused on establishing a biobank of organoids and tumoroids derived from wild-type (WT) or transgenic mice, respectively. Additionally, we generated PCa tumoroids by inducing the deletion of *Pten*, *Stat3*, and *Tp53* in WT organoids *in vitro*. Interestingly, the deletion of the target genes induced the deregulation of metabolic pathways in all knockout (KO) tumoroids. In addition, a medium-throughput compound screen identified the PDPK1/AKT/FLT dual pathway inhibitor (DPI) (also called KP372-1)[34–36] and the epigenetic modifier tenovin-6 (T6), a sirtuin inhibitor and TP53 activator,[37] as promising agents. These compounds effectively inhibited the growth of murine tumoroid models and several human PCa cell lines. Thus, murine tumoroids provide reliable preclinical models for PCa research and could be used to identify treatments for PCa patients based on their genetic background.

RESULTS

Establishment and genetic stability of murine PCa tumoroids reflecting patient mutations

To highlight the importance of modeling genetic mutations of *PTEN*, *STAT3*, and *TP53*, we analyzed publicly available RNA sequencing data from primary PCa patients of the PRAD-TCGA dataset.[38] A lower expression of the tumor suppressor *PTEN*, or the transcription factor *STAT3*, significantly correlated with shorter overall survival time (Figure 1A). Additionally, patients carrying mutations in the tumor suppressor *TP53* had a significantly shorter survival time compared to patients with a WT *TP53* gene. Together, this data underlined the important role of these genes for PCa tumorigenesis and patient prognosis.

To develop reliable preclinical models for PCa research, we took advantage of previously established conditional murine PCa models that harbor deletions of genes highly relevant for human PCa, including *Pten* single KO,[40] *Pten*/*Stat3*,[16] or *Pten*/*Tp53* double KO (dKO)[41] (Figure 1B). Tumoroids were derived from the tumors of these mice at 19 weeks of age[42] (*in vivo* KO), while WT organoids were generated from healthy prostates of Cre-negative mice carrying *loxP*-sites for the respective genes (*in vivo* WT). These WT organoids were subsequently transduced with a tamoxifen-inducible Cre-recombinase to induce the deletion of the respective genes (*in vitro* KO), to investigate the effects of these mutations on malignant transformation. In addition, *in vivo* WT organoids were transduced with a non-functional Cre-recombinase as a control (*in vitro* WT). Taken together, we generated an extensive biobank of murine organoids and tumoroids reflecting common PCa patient mutations associated with different stages of tumor aggressiveness.

The stable KO of the genes of interest in the *in vivo* and *in vitro* tumoroid models was confirmed on DNA and RNA level,

(B) Overview of the experimental design of this study. Tumoroids were derived from prostate tumors of mice with a genetic deletion based on the Cre-*loxP* system of *Pten* alone, or in combination with *Stat3*, or *Tp53* (*in vivo* KO). Healthy organoids were derived from Cre-negative mice with *loxP*-sites for the genes of interest (*in vivo* WT). Organoids with *loxP*-sites were then either transduced with a functional Cre-recombinase to induce the deletion of the genes (*in vitro* KO) or a non-functional Cre-recombinase as a control (*in vitro* WT).

(C) Western blot analysis of murine *in vivo* and *in vitro* organoids and KO tumoroids for indicated genotypes for PTEN, STAT3, TP53, phospho-AKT (pAKT), total AKT (AKT), and representative β-ACTIN as loading control. All samples shown in the *Pten*/*Tp53* KO blot were treated with $CoCl_2$ to induce TP53 expression.

(D) Quantification of protein expression of western blots shown in (C) (top: *Pten* KO, middle *Pten*/*Stat3* KO, bottom: *Pten*/*Tp53* KO). Bar graphs represent relative band intensity of proteins of interest normalized to β-ACTIN as loading control. Data are presented as means of triplicates ± SD. Statistical analysis was performed using Image Lab 6.1 and GraphPad Prism 8.0.2 (one-way ANOVA, Tukey's test). $p > 0.05$ if not specified otherwise, $*p \leq 0.05$; $**p \leq 0.01$, $***p \leq 0.001$, $****p \leq 0.0001$.

(E) Bar graphs depicting proliferation rates of *in vivo* and *in vitro* organoids and tumoroids (1–3 = biological replicates/single clones) for all genotypes over 6 days normalized to day 1 (fold change). Of note, *in vitro* organoid line WT1 has the same maternal line as *in vitro Pten* KO 1–3, WT2 corresponds to *in vitro Pten*/*Stat3* KO 1–3, and WT3 corresponds to *in vitro Pten*/*Tp53* KO 1–3. Each bar represents technical triplicates ±SD per organoid/tumoroid line. See also Figure S1.

whereby the deletion of the targeted exons for *Pten*, *Stat3*, and *Tp53* on DNA level (Figures S1A and S1B) resulted in the complete loss of gene expression in the KO tumoroids (Figure S1C). While all healthy organoid lines showed an expression of PTEN on protein level, the loss of PTEN and a subsequent activation of the PI3K/AKT pathway was seen in the KO tumoroids (Figures 1C and 1D). In addition, the absence of the STAT3 protein was confirmed in the *Pten*/*Stat3* dKO tumoroids. To visualize the expression or loss of TP53, we treated all organoid and tumoroid lines with cobalt chloride ($CoCl_2$) leading to increased stability of TP53.[43] While a heterogeneous expression of TP53 was seen in the WT organoids, the protein was lost completely in the *Pten*/*Tp53* dKO tumoroids (Figures 1C and 1D, bottom). In summary, all 3D models showed a clear loss of the respective proteins of interest, and an activation of the pro-tumorigenic PI3K/AKT pathway. In addition to PI3K/AKT signaling, androgen receptor (AR) signaling is one of the most important survival pathways of PCa cells.[44] Both *in vivo* and *in vitro* murine organoids and tumoroids expressed *Ar* and AR target genes on RNA level (Figure S1D) and AR expression was confirmed on protein level in all organoids and tumoroids (Figure S1E). Interestingly, while *Ar* expression was comparable on RNA level in all organoid and tumoroid lines, protein expression was upregulated in *Pten* and *Pten*/*Tp53* KO tumoroids, suggesting that loss of *Stat3* in the *Pten*/*Stat3* KO tumoroids counteracts this upregulation.[45] Additionally, both the *in vivo* WT organoids and the KO tumoroids showed a response to AR pathway inhibition by enzalutamide with IC50s ranging from 22.86 μM for WT organoids, to 10.86 μM for *Pten*/*Stat3* KO, 18.28 μM for *Pten* KO, and 48.19 μM for *Pten*/*Tp53* KO tumoroids (Figure S1F). As these values are similar to IC50 values of human hormone-sensitive PCa cell lines,[46] our murine organoids and tumoroids represent hormone-sensitive models with active AR signaling.

Importantly, all *in vitro* organoids and KO tumoroids stably reflected the protein expression levels of their *in vivo* counterparts. Based on the negative influence of mutations in *PTEN*, *STAT3*, and *TP53* on PCa patient survival, and the activation of the PI3K/AKT pathway promoting proliferation,[47] we expected a growth advantage of the KO tumoroids compared to WT organoids. Interestingly, we observed heterogeneous proliferation rates among WT organoids and KO tumoroids, with *Pten*/*Tp53* dKO tumoroids showing the highest proliferation rate on average (Figure 1E). As organoid growth medium was optimized for the growth of healthy cells, we hypothesize that the proliferation rate mainly depends on the medium composition.[25] Overall, *in vivo* KO tumoroids exhibited a noticeable trend of accelerated proliferation compared to WT organoids. In addition, exponential growth patterns were observed primarily in *in vivo* KO tumoroids, while *in vitro* KO tumoroids mainly exhibited linear growth patterns.

Murine organoids and PCa tumoroids reflect the morphology of their tissue of origin

To investigate whether the organoids and tumoroids with different genetic backgrounds stably reflect their tissue of origin, we performed histo-morphological analyses including immunohistochemistry (IHC) on tissues and corresponding 3D models (Figures 2 and S2A). The murine healthy prostate tissue consists of glands made up of a two-layered epithelium, visible in hematoxylin and eosin (H&E) staining and IHC for CK8-positive luminal cells and fewer P63-positive basal cells (Figures 2A and S2B). While there were nearly no proliferating cells expressing KI67 in the WT tissue, complex multi-layered, partly cribriform, and invasive glands with increased KI67 expression were observed in the KO tumors. KO tissues were also characterized by an increase in CK8-positive invasive cells and scattered basal cells.

The *in vivo* WT organoids formed mostly large and hollow structures reflecting normal prostate glands, while the *Pten* KO and *Pten*/*Stat3* dKO tumoroids displayed a compact growth pattern resembling irregular tumor tissues (Figure 2B). The *Pten*/*Tp53* dKO tumoroids displayed a slightly different growth pattern and formed the largest tumoroid spheres among all 3D models. In line with previous literature,[23,48] we mostly observed organoids and tumoroids consisting of both luminal and basal cells, with few structures consisting of only one cell type. The proliferation marker KI67 was expressed at similar levels in all organoid and tumoroid lines. Importantly, the *in vitro* deletion of *Pten* alone, or in combination with *Stat3*, or *Tp53* in WT organoids resulted in morphological changes reflected by dense growth patterns as previously observed in *in vivo* KO tumoroids (Figure 2C). Moreover, all *in vitro* KO tumoroids showed cancer-specific increased nuclear atypia in comparison to their healthy counterparts. CK8/P63 distribution and proliferation marked by KI67 were comparable to WT organoids and *in vivo* KO tumoroids (Figure S2B). In addition, we detected nuclear AR expression in all tissues and organoid and tumoroid lines, hinting to active androgen signaling in our models (Figures S3A and S3B). In conclusion, the phenotypic changes in organoid morphology possibly indicate the malignant transformation of WT organoids after genetic deletion of the target genes.

Transcriptomic analysis of PCa tumoroids reveals upregulation of oncogenic signaling and alterations in metabolic pathways

To investigate the impact of PCa-specific mutations on gene expression and signaling, we performed bulk RNA sequencing on *in vivo* WT and KO 3D models. Principal component analysis of biological replicates showed heterogeneity both among and within the different genotypes, with two out of three biological replicates clustering closely together (Figure 3A). The *in vivo WT* organoids also showed some heterogeneity, which might be explained by different ratios between luminal and basal cells in different WT lines and cellular plasticity.[31] The most considerable heterogeneity was apparent in the *Pten*/*Tp53* dKO tumoroids. Unsupervised hierarchical clustering of the top 1,000 most variably expressed genes reflected the previously observed heterogeneity of organoid and tumoroid lines (Figure 3B). Interestingly, *in vivo* 3D models separated independent of their genotype into two main groups, both containing organoids and tumoroids. Differential gene expression between these two groups identified a deregulation of genes and pathways involved in cell cycle regulation and mitosis (Figures S4A–S4E), highlighting the major impact of different proliferation rates on overall gene expression.

CellPress
OPEN ACCESS

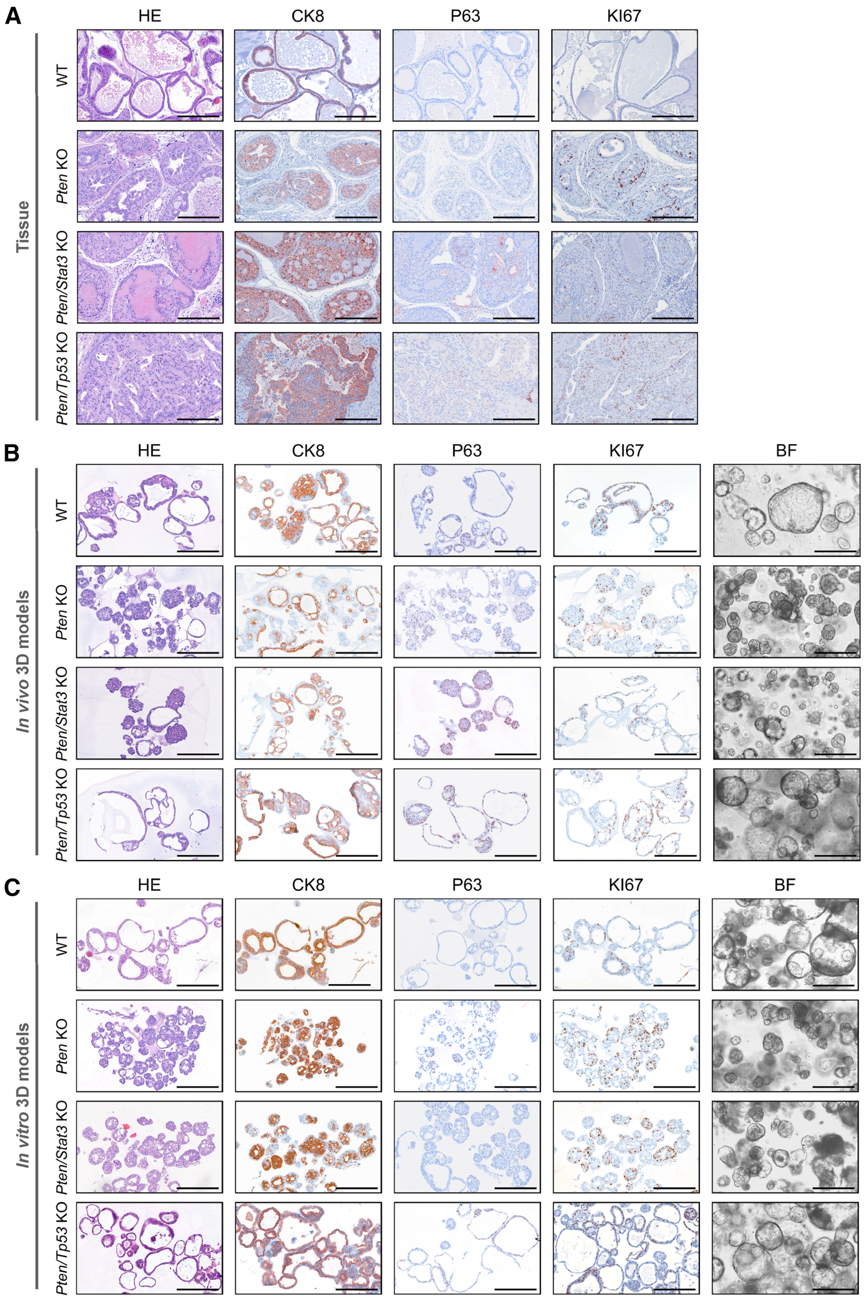

Figure 2. Murine organoids and PCa tumoroids reflect the morphology of their tissue of origin

(A) Comparison between WT prostate tissues and prostate tumors of the *Pten* KO, *Pten/Stat3* KO, and *Pten/Tp53* KO PCa mouse models stained with H&E, or antibodies against KI67 (proliferation), CK8 (luminal cell marker), or P63 (basal cell marker). One representative mouse per genotype is shown ($N = 2$). Scale bars, 200 μm.

(B) Comparison between *in vivo* WT organoids and KO tumoroids for all genotypes. Organoids/tumoroids derived from murine tissues depicted in (A) are shown. In addition to H&E, KI67, CK8, and P63 IHC stainings, bright-field (BF) microscopic images of 3D models are shown. Scale bars, 200 μm.

(C) Comparison between *in vitro* WT organoids and KO tumoroids for all genotypes. In addition to H&E, KI67, CK8, and P63 IHC stainings, BF microscopic images of 3D models are shown. One representative line per genotype is shown ($N = 3$). Scale bars, 200 μm. See also Figures S2 and S3.

Next, we analyzed the genotype-specific significant differentially expressed genes (DEGs) between *in vivo* KO tumoroids and WT organoids (Figures 3C and 3D; Tables S3A–S3C). The KO of *Pten* alone, or a dKO of *Pten* and *Stat3*, only resulted in 57 or 82 significant DEGs, respectively. In contrast, combined *Pten* and *Tp53* KO resulted in 309 significant DEGs (Figure 3C). Four genes, which were all reported to interact with the PI3K/AKT pathway, were highly deregulated in all tumoroid genotypes compared to WT organoids (Figure 3D). Among these, the insulin-induced gene 1 (*Insig1*) and the insulin-like growth factor binding protein 5 (*Igfbp5*) are involved in lipid metabolism and can support cell proliferation.[49,50] Additionally, proline arginine-rich end leucine-rich repeat protein (*Prelp*), an extracellular matrix (ECM) anchoring protein, might be involved in cell adhesion[51] and epithelial-to-mesenchymal transition (EMT).[52] Of note, *PRELP* expression was correlated with the expression of mesenchymal EMT genes *VIM* (Vimentin), *SNAI1*, *TGFB1*, and *ITGA1*, while being anticorrelated with epithelial genes *EPCAM*, *KRT8*, and *DSP* in human TCGA-PRAD expression data (Figure S4F). Lastly, tripartite motif-containing 30D (*Trim30d*) is predicted to be a transcription co-activator and possible E3 ubiquitin ligase, and thus might influence several signaling pathways.[53]

To better understand how the identified DEGs impact broader biological processes, we performed KEGG pathway enrichment analysis (Figure 3E) and studied the connections between DEGs using the String database (Figure S5A). Both *Stat3* and *Tp53* appeared as central points in the String networks, validating the dKO tumoroids as representative models to study the changes in protein interactions after genetic deletion of specific genes. In addition, signaling networks of *Prelp*, *Insig1*, and *Igfbp5* were detected. Importantly, in the *Pten* KO and *Pten/Stat3* dKO tumoroids *Pik3r3*, which is part of a regulatory subunit of the PI3K/AKT pathway, showed interactions with integrins, while a network of immune-related proteins was observed in the *Pten/Tp53* dKOs. Among the most significantly enriched KEGG pathways, we detected several metabolic pathways including choline

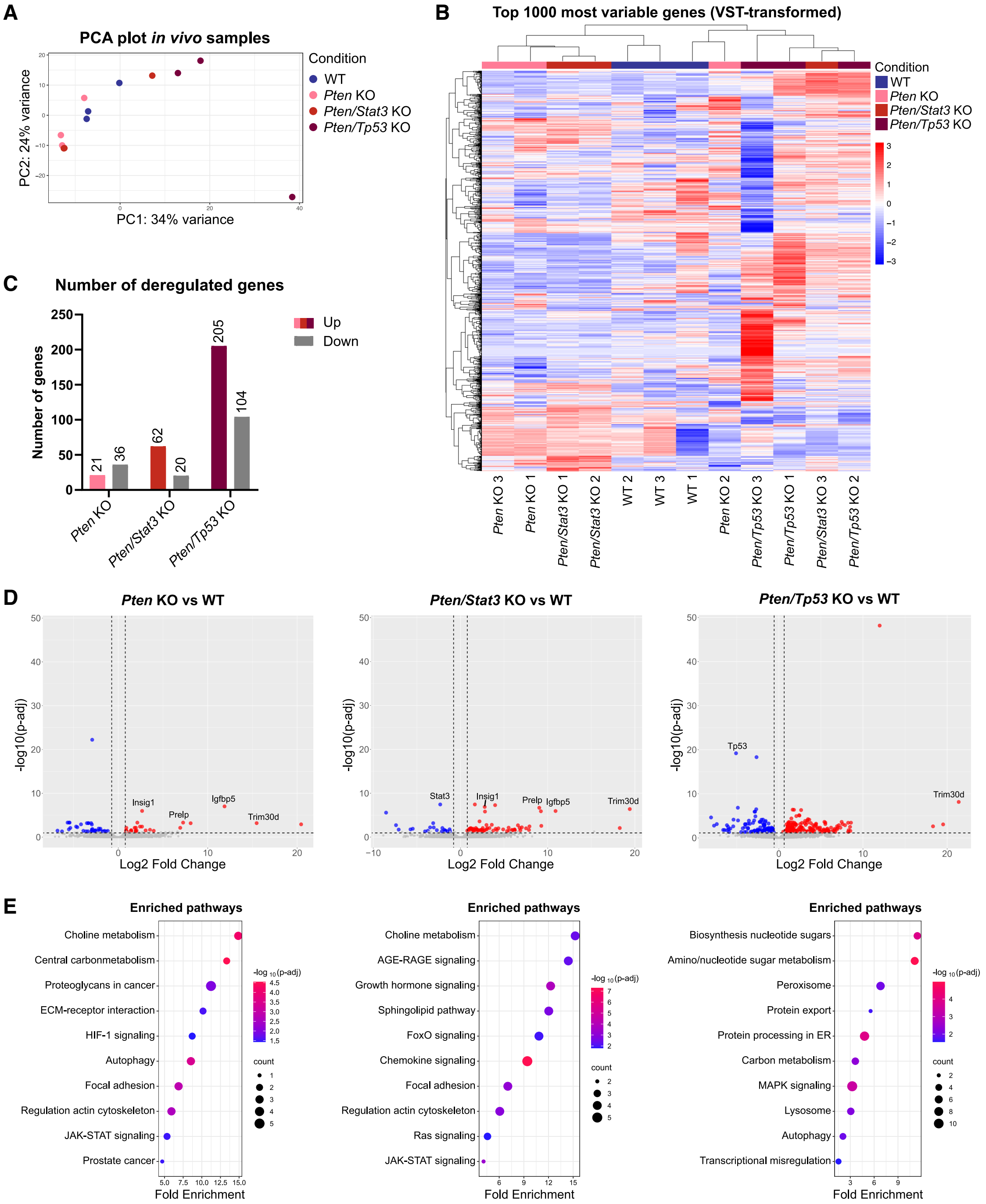

Figure 3. Transcriptomic analysis of PCa tumoroids reveals upregulation of oncogenic signaling and alterations in metabolic pathways

(A) Principal component analysis (PCA) based on bulk RNA sequencing data from *in vivo* WT organoids and indicated KO tumoroids (biological triplicates per genotype).

(B) Dendrogram and heatmap showing unsupervised hierarchical clustering of the top 1,000 most variable genes for all *in vivo* organoids and tumoroids based on VST-normalized gene counts. Rows represent individual genes, while columns represent organoid/tumoroid lines. Colors and intensity reflect expression levels of genes (red: upregulation, blue: downregulation).

(C) Bar graph depicting all significantly overexpressed and downregulated genes (p-adj < 0.05, |Log2fold| > 0) per KO genotype compared to WT organoids (N = 3).

(D) Volcano plots depicting DEGs for *in vivo Pten* KO (left), *Pten/Stat3* KO (middle), and *Pten/Tp53* KO (right) tumoroids compared to WT organoids. Genes with p-adj < 0.05 and Log2fold > 0 (red) or < 0 (blue) are highlighted (N = 3).

(E) Bubble plots showing selected significantly enriched pathways based on the KEGG Pathway Database for *in vivo Pten* KO (left), *Pten/Stat3* KO (middle), and *Pten/Tp53* KO (right) tumoroids compared to WT organoids. Size of points reflects number of DEGs mapped to specific pathways, while color reflects statistical significance (-log10 p-adj) (N = 3). See also Figure S4 and Tables S3A–S3C.

metabolism, the central carbon metabolism, the sphingolipid pathway, and amino/nucleotide sugar metabolism, highlighting the influence of *Pten* loss and PI3K/AKT activation on the metabolism of tumoroid lines (Figure 3E).

Along these lines, several PI3K/AKT-dependent signaling pathways such as the JAK/STAT, FOXO, RAS, and MAPK pathways were deregulated in PCa tumoroids of all genotypes. Additionally, in line with the top DEGs we found an enrichment in focal adhesion and regulation of the actin cytoskeleton. The *Pten*/*Stat3* dKO tumoroids were enriched for chemokine and interferon signaling, which might be a direct effect of the deletion of *Stat3*. While we also detected an enrichment of interferon signaling in the *Pten*/*Tp53* dKO tumoroids, these tumoroids upregulated pathways involved in protein processing and sugar metabolism indicating increased catabolic needs upon dual loss of *Pten* and *Tp53*. In summary, the loss of *Pten*, *Stat3*, and *Tp53* greatly impacted the transcriptional signatures of tumoroids and highlighted their dependency on PI3K/AKT signaling, which induced the deregulation of major pathways related to metabolism and oncogenic signaling.

In vitro deletion of target genes replicates activation of metabolic pathways and oncogenic signaling observed in *in vivo* KO models

To investigate the effect of target gene deletion on the malignant transformation of healthy organoids, we analyzed the differences in gene expression between WT organoids and *in vitro* KO tumoroids, which showed morphological changes upon genetic deletion. For each genotype, we analyzed three single clones derived from the same maternal line upon tamoxifen induction of the Cre-recombinase. The single clones harboring either *Pten*, *Pten*/*Stat3*, or *Pten*/*Tp53* deletions clustered together based on their genotypes (Figure 4A). Interestingly, the WT control organoids did not group together but clustered in close proximity to the respective KO tumoroid lines derived from the same maternal line. Similarly, hierarchical clustering of the top 1,000 most variable genes revealed three clusters, which were dependent on the gene expression of the maternal organoid lines (Figure 4B). Taken together, these results confirmed that the genetic deletion of the target genes *in vitro* changed the gene expression of the organoids but also highlighted the major influence of the transcriptome of their line of origin.

Next, we focused on the significant DEGs between the *in vitro* KO tumoroids and their WT controls (Figures 4C and 4D; Tables S3D–S3F). The deletion of *Pten* alone, or together with *Stat3* resulted in similar numbers of significant DEGs with 101 or 105 genes, respectively. In line with the *in vivo* 3D cultures, the *Pten*/*Tp53* dKO tumoroids showed the highest number of DEGs with 300 genes. Out of all DEGs, the phosphofructokinase enzyme (*Pfkm*), a key player in glycolysis,[54] and Refilin A (*Rflna*), which might influence cell adhesion,[55] were significantly upregulated in all *in vitro* tumoroids. Of note, only *Prelp* was overexpressed in all *in vivo* and *in vitro* KO tumoroids.

To identify major deregulated biological processes in the *in vitro* KO tumoroids, we performed pathway enrichment analysis (Figure 4E) and focused on functional String-networks between the DEGs (Figure S5B). *Stat3* and *Tp53* appeared as central points in the interaction networks, highlighting that their deletion *in vitro* influences major signaling networks. In addition, the interactions of *Prelp* and *Pfkm* were visible in the networks. Importantly, mimicking the *in vivo Pten*/*Tp53* dKO tumoroids, an immune-related network of proteins was also observed in the *in vitro Pten*/*Tp53* dKO models. Even though *in vitro* WT organoids exhibited heterogeneous gene expression determined by different maternal lines, pathway enrichment analysis showed similar results as observed for the *in vivo* tumoroids following deletion of the respective genes. Major changes in metabolic pathways, including the pentose phosphate pathway, fructose/mannose metabolism, carbon metabolism, and glycerophospholipid metabolism were detected upon deletion of *Pten*, *Pten*/*Stat3*, or *Pten*/*Tp53* (Figure 4E). This again highlights the significant role of the PI3K/AKT pathway for metabolic adaptation of cells following *Pten* loss. Interestingly, we found an enrichment in cell cycle and senescence pathways mediated by the upregulation of *Cdkn2a* in the *Pten* KO tumoroids, which has previously been connected to replication stress caused by the loss of tumor suppressor genes.[56] Similar to the *in vivo* KO tumoroids, we observed the deregulation of major signaling pathways like FOXO, AGE/RAGE, and TGFβ signaling. *In vitro* KO of *Pten* and *Tp53* resulted in deregulation of protein processing as well as autocrine chemokine and cytokine signaling, together promoting cancer-specific processes.

Next, we compared the genotype-specific changes in gene expression between *in vivo* and *in vitro* KO tumoroids. Even though the deletion of *Pten* or the co-deletion of *Pten* and *Stat3* resulted in similar numbers of significant DEGs, the overlap between the *in vivo* and *in vitro* KO tumoroids was around 5%, or 18%, respectively (Figure 5A). The *Pten*/*Tp53* dKO tumoroids, which showed the highest number of DEGs overall, shared only 16% of all genes between the conditions. Despite this relatively low overlap, gene set enrichment analysis based on the Hallmark Gene Set Collection[57] revealed an overlap of pathways implicated in tumorigenesis such as the upregulation of KRAS signaling for *Pten* KOs (Figure 5B). In line with the larger overlap of DEGs for the *Pten*/*Stat3* dKO tumoroids, more hallmark gene sets, including EMT, MTORC1 signaling, and interferon-γ response, were shared between the *in vivo* and *in vitro* tumoroids. Interestingly, seven out of ten hallmark gene sets were identical between the *Pten*/*Tp53* dKO tumoroids. Of those, different signaling pathways including MTORC1 and P53 signaling, but also pathways influencing the immune response and inflammation, were enriched.

Of note, the gene sets UV response down and MTORC1 signaling were enriched in all *in vivo* KO tumoroids, indicating a deregulation of stress response, major signaling pathways, and metabolic pathways. The EMT and Hypoxia gene sets were shared between all *in vitro* KO tumoroids hinting to cellular plasticity and an activation of oncogenic pathways after the deletion of the target genes *in vitro*. In conclusion, even though the overlap of significant DEGs is rather small, many biological processes are shared between the *in vivo* and *in vitro* KO tumoroids, especially for the *Pten*/*Stat3* and *Pten*/*Tp53* dKO tumoroids. This suggests that major tumor-driving processes can be replicated *in vitro* and depend on cell-intrinsic mechanisms.

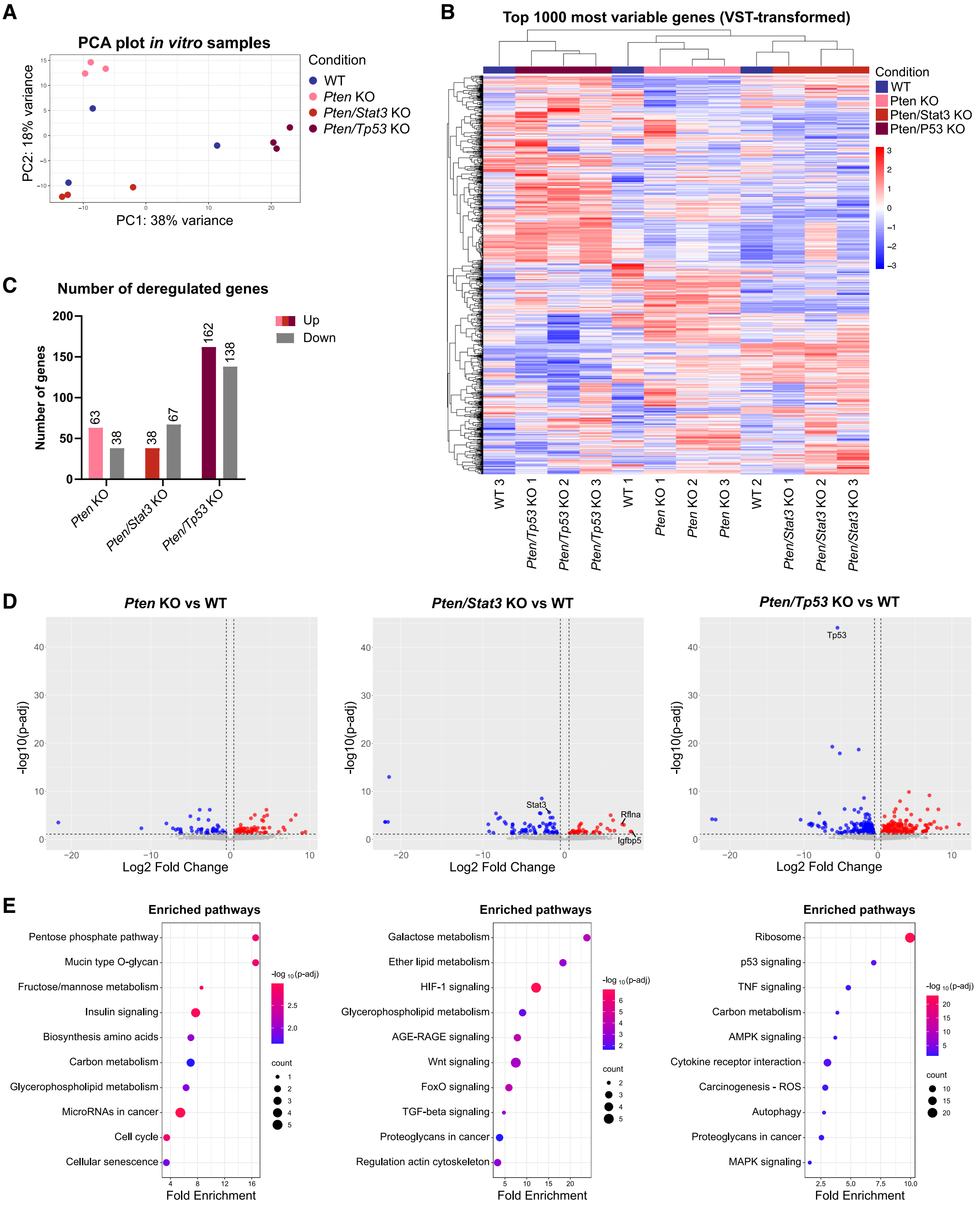

Figure 4. *In vitro* deletion of target genes replicates activation of metabolic pathways and oncogenic signaling observed in *in vivo* KO models

(A) PCA based on bulk RNA sequencing data of *in vitro* WT organoids and indicated KO tumoroids (single clones/triplicates per genotype).

(B) Dendrogram and heatmap showing unsupervised hierarchical clustering of the top 1,000 most variable genes for all *in vitro* organoid and tumoroid lines based on VST-normalized gene counts. Rows represent individual genes, while columns represent organoid/tumoroid lines. Colors and intensity reflect expression levels of genes (red: upregulation, blue: downregulation).

(C) Bar graph depicting all significantly overexpressed and downregulated genes (p-adj < 0.05, |Log2fold| > 0) per KO genotype compared to WT organoids ($N = 3$). (D) Volcano plots depicting DEGs for *in vitro Pten* KO (left), *Pten/Stat3* KO (middle), and *Pten/Tp53* KO (right) tumoroids compared to WT organoids. Genes with p-adj < 0.05 and Log2fold > 0 (red) or < 0 (blue) are highlighted ($N = 3$). (E) Bubble plots showing selected significantly enriched pathways based on the KEGG Pathway Database for *in vitro Pten* KO (left), *Pten/Stat3* KO (middle), and *Pten/Tp53* KO (right) tumoroids compared to WT organoids. Size of points reflects number of DEGs mapped to specific pathways, while color reflects statistical significance (-log10 p-adj) ($N = 3$). See also Figure S5 and Tables S3D–S3F.

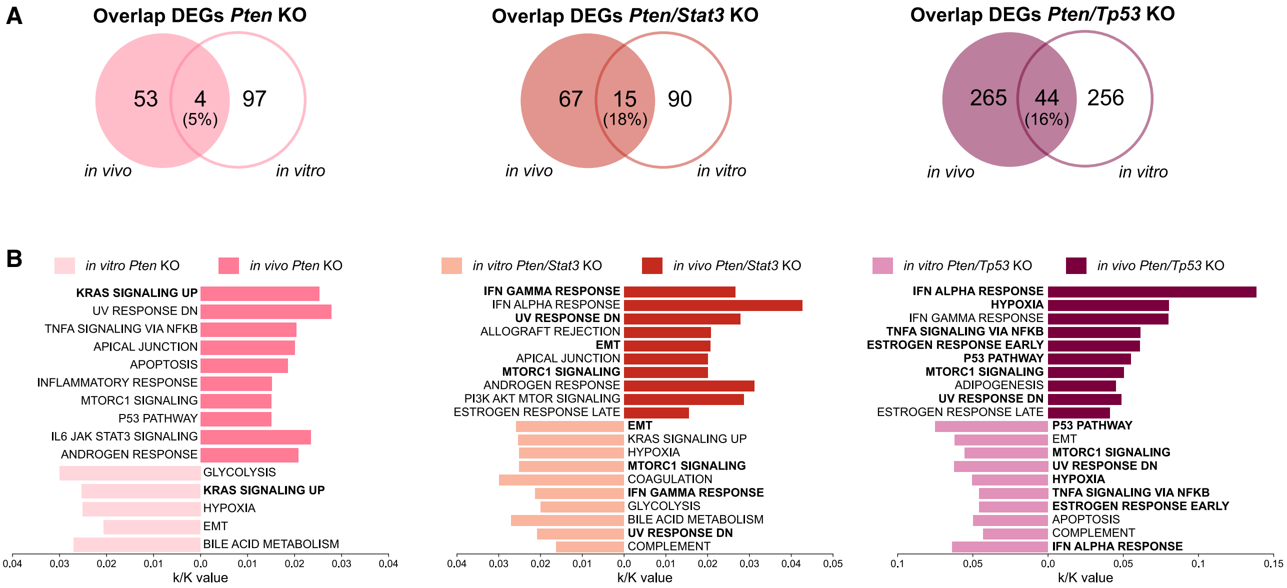

Figure 5. Comparison of DEGs and enriched hallmark gene sets between *in vivo* and *in vitro* KO tumoroids

(A) Overlap of significant DEGs (p-adj < 0.05, |Log2fold| > 0) between *in vivo* and *in vitro Pten* KO (left), *Pten/Stat3* KO (middle), and *Pten/Tp53* KO (right) tumoroids compared to WT organoids (*N* = 3).

(B) Bar graphs depicting the comparison of enriched hallmark gene sets based on MSigDB gene set enrichment analysis of DEGs in (A). *k/K* value describes the ratio of number of genes in input list (*k*) divided by the number of total genes in the gene set of the database (*K*).

Medium-throughput drug screen identified compounds inhibiting PCa tumoroid growth independent of mutational background

As we observed phenotypic and molecular differences between the various KO tumoroid lines, we evaluated the genotype-specific sensitivities of the PCa tumoroids to different pharmaceuticals. For this, we performed a medium-throughput compound screen including 388 common anti-cancer and epigenetic drugs, in addition to selected kinase and pathway inhibitors using small 3D *in vivo* KO tumoroids of all three genotypes (Figures 6A and S6A; Table S4). Following standard high-throughput protocols,[58] compounds were tested at a single concentration of 10 μM, which serves as a high enough concentration to detect active compounds for follow-up testing, but might not reflect lower clinically effective drug concentrations. For all compounds a percentage of control (POC) value was calculated based on positive and negative controls reflecting 0% and 100% cell viability, respectively. Out of 388 tested compounds, 146 induced POC values lower than 50, representing compounds with distinct anti-cancer effects in the first screening. Hierarchical clustering of these hits showed that two out of three KO lines of each genotype were grouped together, while one line clustered separately (Figure 6B). Interestingly, most compounds effectively inhibited tumoroid growth independent of their mutational background, and the number of Hit-compounds was similar among all lines (Figure 6C). However, for the three tumoroid lines that also did not cluster with their respective replicates in the heatmap, more compounds had to be excluded for further analysis, hinting to technical rather than biological effects. Linear regression analysis of compounds shared between two genotypes further supported the fact that the proliferation of tumoroids with different mutations was effectively inhibited by the same compounds (Figure 6D). Based on these results and previously published literature, we selected eight compounds for further analysis (Figure 6E). Among these were several kinase inhibitors, including the EGFR inhibitor afatinib and the Bruton's kinase inhibitor ibrutinib, but also multitargeted kinase inhibitors AT9283 and the PDPK1/AKT/FLT DPI. On the other hand, we focused on four epigenetic modifiers, including the histone demethylase inhibitor GSKJ4, the methyltransferase inhibitor GSK126, and two histone deacetylase (HDAC) inhibitors entinostat and tenovin-6 (T6). Half-maximal inhibitory screening (IC50) revealed that most compounds inhibited tumoroid growth consistently around 1–15 μM, which represent clinically relevant and translatable IC50 concentrations, while GSKJ4 and GSK126 showed high heterogeneity even between biological replicates. High doses of entinostat, which usually exhibits IC50 values between 0.5 and 10 μM on cancer cell lines,[59] were necessary to inhibit PCa tumoroid growth (Figure S6B).

Based on the results of the transcriptomic analysis and our compound screening, we chose the DPI, targeting kinases involved in PI3K/AKT signaling, and T6, an inhibitor of sirtuin HDACs and TP53 activator, for further analysis. IC50 screening of *in vivo* and *in vitro* KO tumoroids of all genotypes revealed highly similar sensitivities of both tumoroid models (Figure 7A). Notably, dKO tumoroids showed higher sensitivity to DPI and T6 treatments, with significant differences for *in vivo* dKOs (Figure 7B). Thus, both compounds showed higher efficiencies on more advanced PCa models, and the effect of different genetic deletions on drug response was recapitulated in *in vitro* tumoroids. Of note, the medium-throughput drug screen was performed on tumoroids seeded in suspension, while for the final

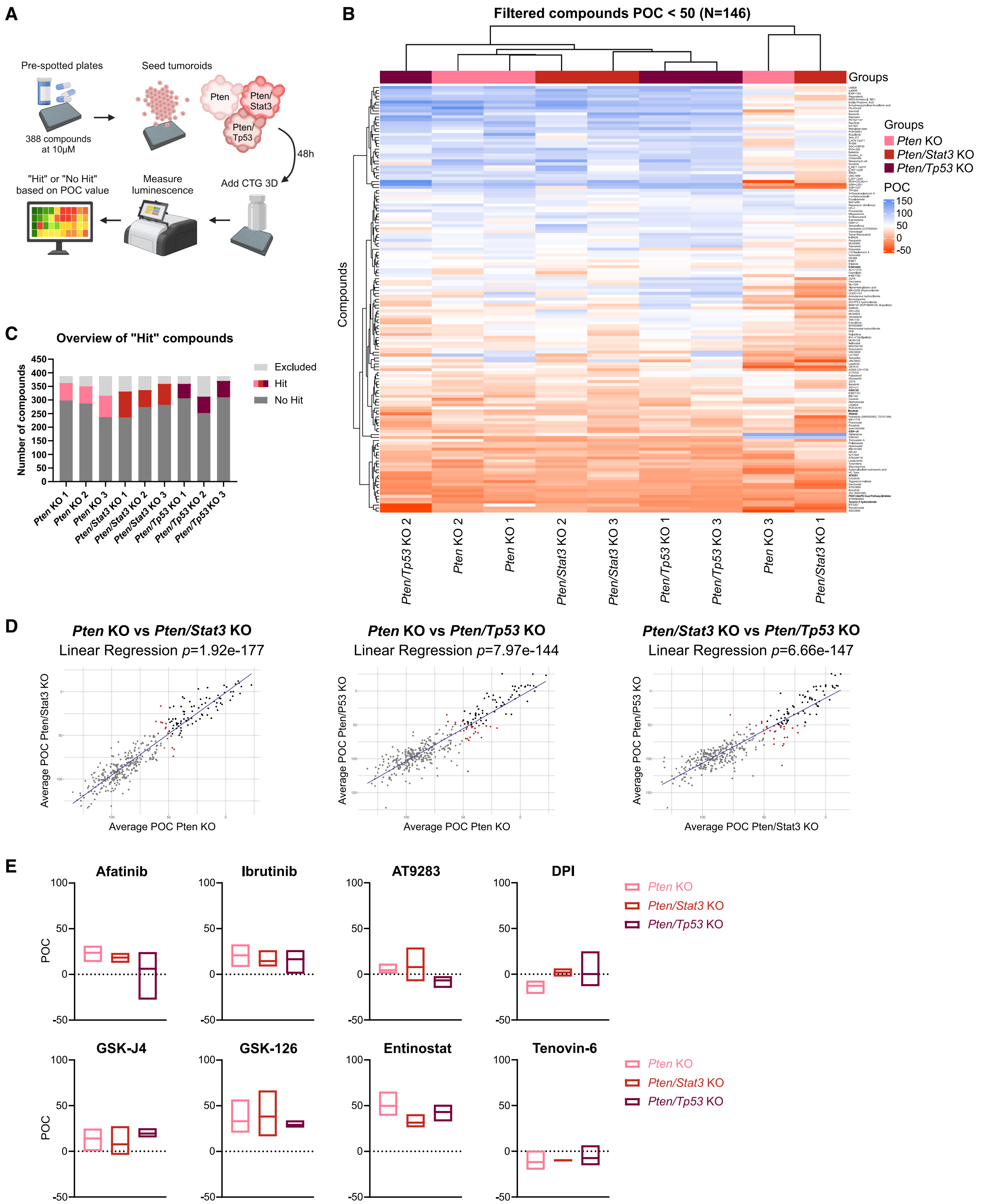

(legend on next page)

confirmation all lines were cultured in ECM domes. Even though it has been reported that the ECM can influence the drug response of tumoroids *in vitro*,[60] we did not observe major differences in IC50 values (Figure S6C). In addition, we investigated the effect of DPI and T6 on healthy WT organoids (Figure S6D). Interestingly, WT organoids and KO tumoroids showed similar IC50 values. However, as WT organoids displayed similar proliferation rates to PCa tumoroid lines, they may not fully represent non-proliferative healthy prostate tissue. Therefore, while the WT response provides a useful baseline, drug sensitivity in preclinical screens should be interpreted with caution regarding physiological relevance. To further elaborate on this, we tested whether there is a difference in response to the targeted pan-AKT kinase inhibitor capivasertib.[61] As expected, both *Pten* and *Pten/Stat3* KO tumoroids were highly sensitive to AKT pathway inhibition with IC50 values of 1.12 and 0.74 μM respectively, while WT organoids showed much higher IC50 values of 19.82 μM. Interestingly, *Pten/Tp53* KO tumoroids were less sensitive with an IC50 concentration of 25.59 μM, which might be due to their inability to induce proper programmed cell death,[62] or the induction of other compensatory signaling pathways (Figure S6E). Together, these data suggest genotype-specific drug sensitivities of WT organoids and tumoroid lines.

To test a potential prognostic significance of genes targeted by DPI and T6 for PCa, we explored the overall survival times of PCa patients dependent on low/high expression levels of the genes of interest.[38] While the DPI targets, *PDPK1*, *AKT1*, *AKT2*, *AKT3*, and *FLT3*, and the T6 targets *SIRT1*, *SIRT2*, and *DHODH* showed no significant correlation with survival on RNA level, high expression of the T6 target *SIRT3* was significantly associated with worse overall survival (Figure S7A). However, as most of these genes code for effector proteins, their expression on RNA level might not reflect their role for PCa signaling.[63]

To confirm the anti-cancer effect of the selected compounds on human cells, we analyzed the cell viability of human PCa cell lines including one primary and three metastatic lines after DPI or T6 treatment (Figure 7C). Indeed, both DPI and T6 effectively inhibited the proliferation of the human cell lines with IC50 concentrations in the same range as for the different tumoroids, confirming that murine PCa tumoroids model the drug response of patient-derived cell line models. Of note, the effect of the two compounds was higher on the metastatic cell lines, indicating that more progressed tumors might be more sensitive to the treatment with DPI and T6. As enzalutamide is one of the most used antiandrogen compounds for the treatment of advanced PCa patients, we used the androgen-responsive LNCaP cell line to investigate whether DPI and T6 could be used in combination with enzalutamide to improve its anti-tumor effect (Figures 7D and S7B). Indeed, both DPI and T6 showed high synergy scores with enzalutamide in the range of the respective IC50 concentrations. Importantly, even low concentrations of enzalutamide in combination with multiple T6 concentrations resulted in high synergy values. In conclusion, both compounds improved the anti-cancer effect of enzalutamide and could thus be beneficial especially for advanced and castration-resistant PCa patients.

DISCUSSION

In recent years, both human and murine organoid and tumoroid models have been used for studying tumorigenesis and finding treatment options for cancer patients as they stably reflect the genetic and epigenetic background, but also the drug response of patients or mouse models.[21] However, patient-derived prostate organoids and PCa tumoroids have low establishment rates and cannot be maintained for several passages *in vitro*.[25,27–30] Here, we established a biobank of organoids and PCa tumoroids derived from murine tumors reflecting common patient mutations and compared them to tumoroids generated by genetic deletion of target genes *in vitro*.

Interestingly, RNA sequencing and pathway enrichment analysis of murine PCa tumoroids suggested a metabolic adaption of healthy cells upon deletion of target genes. In humans, both the healthy prostate and PCa tumors exhibit unique metabolic dependencies.[64,65] While the TCA cycle is suppressed in healthy prostate cells, the development of PCa leads to a metabolic switch by activating the TCA cycle and OXPHOS. Early-stage tumors also heavily rely on lipid and amino acid metabolism for energy production and growth. During advanced and castration-resistant stages of PCa, glycolysis is enhanced (Warburg effect[66]), but OXPHOS and lipid metabolism remain active.[64,65] Importantly, murine PCa models have been used to study PCa metabolism as they recapitulate the metabolic switch observed in humans.[67,68]

Enriched pathways in *in vivo Pten* KO and *Pten/Stat3* dKO tumoroids were mainly mediated by the upregulation of *Pik3r3*, which is part of a regulatory subunit of the PI3K/AKT pathway.[69] High *PIK3R3* expression in combination with the loss of *Pten* leads to the constant activation of the PI3K/AKT pathway,[70] which we also observed on protein level. In addition, aberrant lipid metabolism has been observed in PCa, and choline-PET is used to monitor the progression and therapy response of

Figure 6. Medium-throughput drug screen identified compounds inhibiting PCa tumoroid growth independent of mutational background

(A) Experimental setup of medium-throughput compound screening. 388 compounds were screened at a single dose of 10 μM on small tumoroids with different genetic backgrounds in suspension. CellTiter-Glo 3D (CTG) was added, and luminescence was measured to calculate the POC value representing cell viability.

(B) Dendrogram and heatmap showing unsupervised hierarchical clustering of compounds with POC < 50 ($N = 146$) for all tumoroid lines. Rows represent individual compounds, while columns represent *in vivo* KO tumoroids ($N = 3$). Colors and intensity reflect POC values (red: inhibition of growth, blue: no effect).

(C) Bar graph depicting the number of "Hit" (POC < 50) and "No Hit" (POC > 50) compounds per tumoroid line. Compounds were excluded when POC values of duplicates did not match.

(D) Linear regression analysis based on POC values for all compounds for *Pten* KO ($N = 3$) vs. *Pten/Stat3* KO ($N = 3$), $p = 1.92e-177$ (left); *Pten* KO vs. *Pten/Tp53* KO ($N = 3$), $p = 7.97e-144$ (middle); and *Pten/Stat3* KO vs. *Pten/Tp53* KO, $p = 6.66e-147$ (right). Gray: "No Hit", Black: "Hit", Red: "Genotype-specific "Hit."

(E) Boxplots showing mean ± SD of POC values for selected compounds effective on all genotypes ($N = 3$ per genotype). Negative POC values are the result of tested compounds showing a higher inhibitory effect than the positive control bortezomib. Statistical analysis was performed using GraphPad Prism 8.0.2 (one-way ANOVA, Tukey's test). $p > 0.05$ if not specified otherwise. See also Figures S6 and S7.

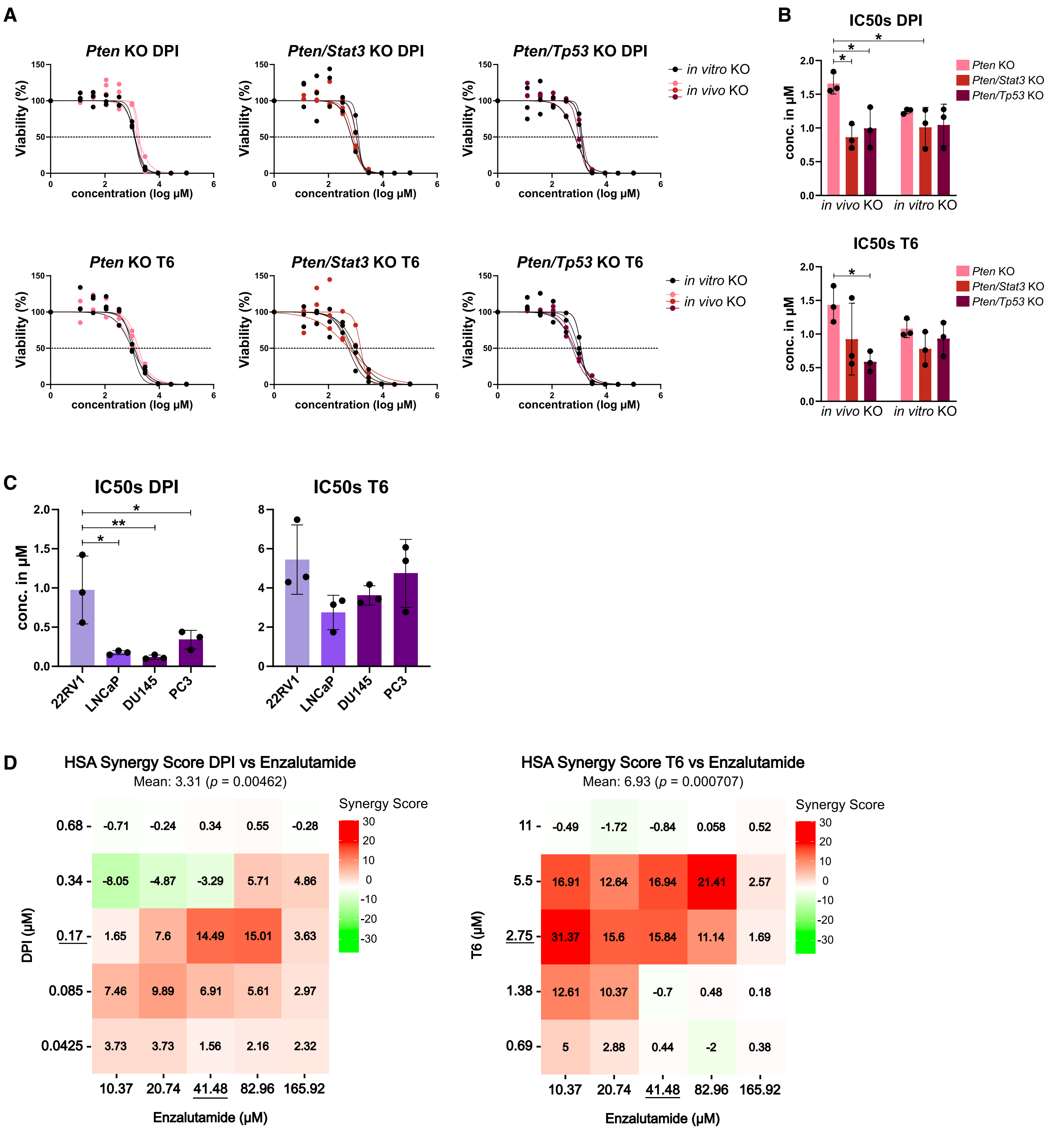

Figure 7. The PDPK1/AKT/FLT DPI and tenovin-6 (T6) show high anti-cancer efficacy in murine tumoroids and human PCa cell lines

(A) Dose-response curves for DPI (top) and T6 (bottom) for *in vivo* and *in vitro* *Pten* KO (left), *Pten/Stat3* KO (middle), and *Pten/Tp53* KO (right) tumoroids. Points represent means of technical duplicates per tumoroid line ($N = 3$). Curve fitting was performed using GraphPad Prism 8.0.2.

(B) Bar graphs showing means and ±SD of half-maximal inhibitory concentration (IC50) for DPI (top) and T6 (bottom) for *in vivo* and *in vitro* tumoroid lines of all genotypes ($N = 3$). Statistical analysis was performed using GraphPad Prism 8.0.2 (one-way ANOVA, Tukey's test). $p > 0.05$ if not specified otherwise, $^*p \leq 0.05$.

(C) Bar graphs depicting means and ±SD of IC50 values of DPI (left) and T6 (right) on human PCa cell lines. 22RV1: primary PCa; LNCaP: metastatic PCa; DU145, PC3: metastatic castration-resistant PCa ($N = 3$). Statistical analysis was performed using GraphPad Prism 8.0.2 (one-way ANOVA). $p > 0.05$ if not specified otherwise, $^*p \leq 0.05$; $^{**}p \leq 0.01$.

(D) Heatmaps of synergy scores calculated with the highest single agent (HSA) model for DPI and enzalutamide (left), and T6 and enzalutamide (right) on the human LNCaP cell line. Values > 0 represent synergistic effects, and values < 0 represent antagonistic effects. IC50 concentrations of respective compounds are underlined ($N = 3$). See also Figure S6.

PCa.[71] We also observed the upregulation of *Pld1*, which mediates PI3K/AKT and mTOR signaling, but also choline metabolism, and thus promotes proliferation and castration resistance in PCa.[72–74]

Even though different genes were deregulated in the *Pten/Tp53* dKO tumoroids, several genes directly involved in glycolysis, including *Hk1*, which is connected to AKT1,[75] *Pgm1*, and *Gfpt1*, were upregulated. Importantly, *Pfkm*, which is also directly involved in glycolysis, showed higher expression in all *in vitro* KO tumoroids. Together, this highlights the major role of the PI3K/AKT pathway in PCa progression but also metabolism of PCa cells.[76] Importantly, we observed changes in metabolic pathways connected to PCa in tumoroids of all three genotypes. *In vitro* KO tumoroids showed similar enrichment of metabolic signaling upon deletion of target genes, highlighting the potential impact of *Pten* loss on metabolic reprogramming during the first steps of tumorigenesis.

On the other hand, increased activation of the PI3K/AKT pathway together with androgen and TGFβ signaling can induce EMT in PCa to drive metastasis and therapy resistance.[77] Interestingly, the only gene that was highly upregulated in all *in vivo* and *in vitro* KO tumoroid lines was the proteoglycan *Prelp*, and both its overexpression[52] and downregulation[78,79] have been associated with tumor progression and EMT. In colorectal cancer, PRELP interacts with integrins to reduce the stiffness of the ECM to drive metastasis.[52] Moreover, the analysis of human PCa transcriptomic data revealed that high expression of *PRELP* correlates with high expression of mesenchymal EMT genes. Together with the enrichment of TGFβ signaling and EMT gene sets in the murine tumoroids, these results suggest that *Prelp* might support EMT and thus PCa progression in our models and reflects a state of high cellular plasticity.[31,77]

Even though the deletion of *Pten* and the consequent activation of the PI3K/AKT pathway have been explored as a therapeutic option for PCa, most PI3K/AKT inhibitors failed as monotherapies during early clinical testing, mostly due to compensatory signaling mechanisms.[80] It has been proposed that multitarget kinase inhibitors are a more promising approach for PCa treatment.[81,82] Out of 388 compounds we identified the DPI,[34–36] which simultaneously inhibits the kinases AKT, and PDPK1 and FLT3 involved in the phosphorylation and thus the complete activation of AKT.[83,84] DPI has shown promising inhibitory effects in multiple cancer types, notably also in combination with PARP inhibitors.[85–88] However, although PI3K/AKT signaling plays a major role in PCa development and progression, the inhibitor has not been widely tested as a treatment for PCa.[89] Here, we show that DPI potently inhibits the proliferation of PCa tumoroids and human PCa cell lines, with an even higher effect on more advanced models. The AR pathway is constitutively active in 22RV1 cells due to the AR-V7 splice variant,[90] and *PTEN* is expressed in these cells. On the other hand, PC3 and LNCaP cells do not express *PTEN*, and the AR pathway is inactive in DU145 cells. This might lead to a dependency on PI3K/AKT signaling, which could explain the increased sensitivity to DPI in the metastatic PCa cell lines.[90] As therapy options for advanced PCa patients are limited, DPI could be a treatment option for these patients.

Apart from metabolic alterations, epigenetic reprogramming is essential for PCa progression and therapy resistance.[91] Several epigenetic compounds have been investigated as treatment options for PCa, and especially HDAC inhibitors have been tested extensively in preclinical and clinical studies.[92] Interestingly, our medium-throughput compound screen identified T6 as one of the most effective inhibitors of PCa tumoroid growth. T6 inhibits SIRT1, SIRT2, SIRT3, and the enzyme DHODH.[37,93] While SIRT1 and SIRT2 can activate the PI3K/AKT pathway to promote proliferation, migration, and neuroendocrine differentiation of PCa cells, SIRT3 usually acts as a tumor suppressor.[94–96] However, patients with high expression of *SIRT3* in the PRAD-TCGA dataset have shorter overall survival time, hinting to a tumor-promoting effect in PCa. In addition, high *DHODH* expression has been correlated with worse prognosis in PCa patients.[97] DHODH is involved in the synthesis of pyrimidines, which are needed for the biosynthesis of DNA, RNA, glycoproteins, and phospholipids.[98] Thus, highly proliferative cancer cells might be more sensitive to inhibition of nucleotide synthesis. Indeed, we observed a stronger effect of T6 on *Pten/Tp53* dKO tumoroids, which showed the highest proliferation rates among the different genotypes. Importantly, we also confirmed the anti-cancer effect of T6 on several human PCa cell lines and propose this compound as a potential therapy option for PCa that has not yet been investigated for this cancer type.

So far, epigenetic compounds are not used as single treatments for solid cancers, and the combination with chemotherapeutics or antiandrogens has shown promising results for PCa.[92] In line with this, the inhibition of PI3K/AKT in combination with androgen signaling has emerged as a treatment strategy.[80] Importantly, we showed that both DPI and T6 in combination with enzalutamide synergistically inhibit human PCa cell proliferation, and could thus be used to increase the anti-cancer effect of enzalutamide. Future studies should validate the mechanisms of DPI and T6 in both *in vitro* and *in vivo* PCa models.

In conclusion, by using murine PCa tumoroids, we identified two promising compounds for further validation for PCa treatment. As tumoroids replicated the drug response of human PCa cell lines, they could help reduce the number of animal models used for cancer research in line with the 3R principles.[99] While *in vivo* KO tumoroids capture the tumor development within the native microenvironment and thus might better reflect the heterogeneity of PCa lesions, our *in vitro* KO tumoroids demonstrate that the effect of mutations on gene expression and drug response can be modeled *in vitro* without the use of further animals. Additional mutations can easily be introduced for a personalized medicine approach, and the malignant transformation of cells can be studied over time to gain further insights into PCa development.

Limitations of the study

Even though tumoroids present useful preclinical models, they do not reflect the complex interactions of cancer cells with the tumor microenvironment.[21] In addition, we and others[100] did not observe an effect of the ECM on drug response, but its negative influence has previously been described.[60] Thus, refining

tumoroid culture conditions by incorporating components of the TME and physical stimuli could enhance their physiological relevance for drug development.[21,101,102] Whenever possible, healthy control organoids should be included in the initial compound screening to exclude compounds with general cytotoxicity. As we observed similar proliferation rates between WT organoids and tumoroids, employing differentiation protocols may better mimic non-proliferative, healthy epithelial tissues.[103] Although we were able to show that prostate organoids and PCa tumoroids express AR on RNA and protein level, and respond to AR pathway inhibition by enzalutamide, we were not able to reliably detect the AR target NKX3.1 using IHC. Here, further optimization or alternative detection methods are needed. Lastly, since our compounds were effective in castration-resistant human PCa cell lines, establishing castration-resistant tumoroids with defined mutational backgrounds could further support the development of targeted therapies for advanced disease.

RESOURCE AVAILABILITY

Lead contact

Requests for further information and resources should be directed to and will be fulfilled by the lead contact, Gerda Egger (gerda.egger@meduniwien.ac.at).

Materials availability

All organoid and tumoroid lines generated in this study are available from the lead contact. We are glad to share all models with reasonable compensation by requestor for its processing and shipping, and a completed materials transfer agreement.

Data and code availability

- Bulk RNA sequencing data were deposited at Gene Expression Omnibus [GSE291912] and are publicly available as of the date of publication. This paper analyses existing, publicly available data, accessible at the TCGA (Accession number phs000178).
- No original code has been generated during this study. Publicly available code and packages are cited in the text or method section.
- Any additional information required to reanalyze the data reported in this paper is available from the lead contact upon request.

ACKNOWLEDGMENTS

The authors thank Sabrina Wohlhaupter, Astrid Haase, Barbara Neudert, and Michaela Schlederer for performing IHC stainings and Martin Raigel for help with pathological analysis. This research was funded by the Austrian Science Fund (FWF) (10.55776/P32771, 10.55776/DOC59, and 10.55776/F8300). Z.P. was supported by an FFG-FEMtech scholarship (no. 8743637). K.M. received funding from the European Union's Horizon 2020 Marie Skłodowska-Curie Innovative Training Networks (ITN-ETN) FANTOM under grant agreement no. 101072735. For open access purposes, the author has applied a CC BY public copyright license to any author accepted manuscript version arising from this submission. Figures were partially created using BioRender.

AUTHOR CONTRIBUTIONS

Conceptualization, G.E., T.D., and J.K.; methodology, J.K., T.D., Z.P., T.L., T.M., L.V., and S.K.; formal analysis, J.K., K.D., A.T., and G.W.; investigation, J.K., T.D., R.J., E.A., K.M., and A.B.; resources, T.L. and L.K.; writing – original draft, J.K. and G.E.; writing – review and editing, all; visualization, J.K. and G.E.; supervision, G.E.; project administration, J.K. and G.E.; funding acquisition, G.E. and L.K.

DECLARATION OF INTERESTS

The authors declare no competing interests.

DECLARATION OF GENERATIVE AI AND AI-ASSISTED TECHNOLOGIES IN THE WRITING PROCESS

During the preparation of this work, the authors used ChatGPT (OpenAI) in order to improve grammar and clarity of the text. After using this tool, the authors reviewed and edited the content as needed and take full responsibility for the content of the publication.

STAR★METHODS

Detailed methods are provided in the online version of this paper and include the following:

- KEY RESOURCES TABLE
- EXPERIMENTAL MODEL AND STUDY PARTICIPANT DETAILS
  - Animal models
  - Murine PCa organoids and tumoroids
  - Human PCa cell lines
- METHOD DETAILS
  - Analysis of human publicly available data
  - Lentiviral transduction
  - Genotyping PCR
  - qRT-PCR
  - Western blotting
  - Proliferation assay
  - Immunohistochemistry
  - RNA sequencing and gene expression analysis
  - Medium-throughput compound screen
  - Validation of compounds on murine tumoroids
  - Validation of compounds on human cell lines
  - Synergy assay
- QUANTIFICATION AND STATISTICAL ANALYSIS

SUPPLEMENTAL INFORMATION

Supplemental information can be found online at https://doi.org/10.1016/j.crmeth.2026.101370.

Received: October 29, 2025
Revised: January 30, 2026
Accepted: February 26, 2026
Published: March 30, 2026

REFERENCES

1. James, N.D., Tannock, I., N'Dow, J., Feng, F., Gillessen, S., Ali, S.A., Trujillo, B., Al-Lazikani, B., Attard, G., Bray, F., et al. (2024). The Lancet Commission on prostate cancer: planning for the surge in cases. Lancet *403*, 1683–1722. https://doi.org/10.1016/S0140-6736(24)00651-2.
2. Crowley, L., and Shen, M.M. (2022). Heterogeneity and complexity of the prostate epithelium: New findings from single-cell RNA sequencing studies. Cancer Lett. *525*, 108–114. https://doi.org/10.1016/j.canlet.2021.10.035.
3. Haffner, M.C., Zwart, W., Roudier, M.P., True, L.D., Nelson, W.G., Epstein, J.I., De Marzo, A.M., Nelson, P.S., and Yegnasubramanian, S. (2021). Genomic and phenotypic heterogeneity in prostate cancer. Nat. Rev. Urol. *18*, 79–92. https://doi.org/10.1038/s41585-020-00400-w.
4. Flores-Téllez, T.D.N.J., Baena, E., and Baena, E. (2022). Experimental challenges to modeling prostate cancer heterogeneity. Cancer Lett. *524*, 194–205. https://doi.org/10.1016/j.canlet.2021.10.012.
5. Kaushal, J.B., Takkar, S., Batra, S.K., and Siddiqui, J.A. (2024). Diverse landscape of genetically engineered mouse models: Genomic and

CellPress
OPEN ACCESS

molecular insights into prostate cancer. Cancer Lett. *593*, 216954. https://doi.org/10.1016/j.canlet.2024.216954.

6. Rebello, R.J., Oing, C., Knudsen, K.E., Loeb, S., Johnson, D.C., Reiter, R.E., Gillessen, S., Van der Kwast, T., and Bristow, R.G. (2021). Prostate cancer. Nat. Rev. Dis. Primers *7*, 9–27. https://doi.org/10.1038/s41572-020-00243-0.
7. Almeeri, M.N.E., Awies, M., and Constantinou, C. (2024). Prostate Cancer, Pathophysiology and Recent Developments in Management: A Narrative Review. Curr. Oncol. Rep. *26*, 1511–1519. https://doi.org/10.1007/s11912-024-01614-6.
8. Yamada, Y., and Beltran, H. (2021). The treatment landscape of metastatic prostate cancer. Cancer Lett. *519*, 20–29. https://doi.org/10.1016/j.canlet.2021.06.010.
9. Cotter, K., and Rubin, M.A. (2022). The evolving landscape of prostate cancer somatic mutations. Prostate *82*, S13–S24. https://doi.org/10.1002/pros.24353.
10. Jamaspishvili, T., Berman, D.M., Ross, A.E., Scher, H.I., De Marzo, A.M., Squire, J.A., and Lotan, T.L. (2018). Clinical implications of PTEN loss in prostate cancer. Nat. Rev. Urol. *15*, 222–234. https://doi.org/10.1038/nrurol.2018.9.
11. Armenia, J., Wankowicz, S.A.M., Liu, D., Gao, J., Kundra, R., Reznik, E., Chatila, W.K., Chakravarty, D., Han, G.C., Coleman, I., et al. (2018). The long tail of oncogenic drivers in prostate cancer. Nat. Genet. *50*, 645–651. https://doi.org/10.1038/s41588-018-0078-z.
12. Don-Doncow, N., Marginean, F., Coleman, I., Nelson, P.S., Ehrnström, R., Krzyzanowska, A., Morrissey, C., Hellsten, R., and Bjartell, A. (2017). Expression of STAT3 in Prostate Cancer Metastases. Eur. Urol. *71*, 313–316. https://doi.org/10.1016/j.eururo.2016.06.018.
13. Abdulghani, J., Gu, L., Dagvadorj, A., Lutz, J., Leiby, B., Bonuccelli, G., Lisanti, M.P., Zellweger, T., Alanen, K., Mirtti, T., et al. (2008). Stat3 Promotes Metastatic Progression of Prostate Cancer. Am. J. Pathol. *172*, 1717–1728. https://doi.org/10.2353/ajpath.2008.071054.
14. Han, Z., Wang, X., Ma, L., Chen, L., Xiao, M., Huang, L., Cao, Y., Bai, J., Ma, D., Zhou, J., and Hong, Z. (2014). Inhibition of STAT3 signaling targets both tumor-initiating and differentiated cell populations in prostate cancer. Oncotarget *5*, 8416–8428. https://doi.org/10.18632/oncotarget.2314.
15. Tuo, Z., Zhang, H., He, K., Jiang, Z., Jiang, C., Chen, X., and Yuan, H. (2024). Pan-cancer analysis of STAT3 indicates its potential prognostic value and correlation with immune cell infiltration in prostate cancer. Discov Onc *15*, 654. https://doi.org/10.1007/s12672-024-01527-7.
16. Pencik, J., Schlederer, M., Gruber, W., Unger, C., Walker, S.M., Chalaris, A., Marié, I.J., Hassler, M.R., Javaheri, T., Aksoy, O., et al. (2015). STAT3 regulated ARF expression suppresses prostate cancer metastasis. Nat. Commun. *6*, 7736. https://doi.org/10.1038/ncomms8736.
17. Pencik, J., Philippe, C., Schlederer, M., Atas, E., Pecoraro, M., Grund-Gröschke, S., Li, W.J., Tracz, A., Heidegger, I., Lagger, S., et al. (2023). STAT3/LKB1 controls metastatic prostate cancer by regulating mTORC1/CREB pathway. Mol. Cancer *22*, 133. https://doi.org/10.1186/s12943-023-01825-8.
18. Mai, C.-W., Chin, K.-Y., Foong, L.-C., Pang, K.-L., Yu, B., Shu, Y., Chen, S., Cheong, S.-K., and Chua, C.W. (2022). Modeling prostate cancer: What does it take to build an ideal tumor model? Cancer Lett. *543*, 215794. https://doi.org/10.1016/j.canlet.2022.215794.
19. Cunningham, D., and You, Z. (2015). In vitro and in vivo model systems used in prostate cancer research. J. Biol. Methods *2*, e17. https://doi.org/10.14440/jbm.2015.63.
20. Sailer, V., von Amsberg, G., Duensing, S., Kirfel, J., Lieb, V., Metzger, E., Offermann, A., Pantel, K., Schuele, R., Taubert, H., et al. (2023). Experimental in vitro, ex vivo and in vivo models in prostate cancer research. Nat. Rev. Urol. *20*, 158–178. https://doi.org/10.1038/s41585-022-00677-z.
21. Kalla, J., Pfneissl, J., Mair, T., Tran, L., and Egger, G. (2025). A systematic review on the culture methods and applications of 3D tumoroids for cancer research and personalized medicine. Cell. Oncol. *48*, 1–26. https://doi.org/10.1007/s13402-024-00960-8.
22. Gao, D., Vela, I., Sboner, A., Iaquinta, P.J., Karthaus, W.R., Gopalan, A., Dowling, C., Wanjala, J.N., Undvall, E.A., Arora, V.K., et al. (2014). Organoid cultures derived from patients with advanced prostate cancer. Cell *159*, 176–187. https://doi.org/10.1016/j.cell.2014.08.016.
23. Karthaus, W.R., Iaquinta, P.J., Drost, J., Gracanin, A., van Boxtel, R., Wongvipat, J., Dowling, C.M., Gao, D., Begthel, H., Sachs, N., et al. (2014). Identification of multipotent luminal progenitor cells in human prostate organoid cultures. Cell *159*, 163–175. https://doi.org/10.1016/j.cell.2014.08.017.
24. Puca, L., Bareja, R., Prandi, D., Shaw, R., Benelli, M., Karthaus, W.R., Hess, J., Sigouros, M., Donoghue, A., Kossai, M., et al. (2018). Patient derived organoids to model rare prostate cancer phenotypes. Nat. Commun. *9*, 2404. https://doi.org/10.1038/s41467-018-04495-z.
25. Drost, J., Karthaus, W.R., Gao, D., Driehuis, E., Sawyers, C.L., Chen, Y., and Clevers, H. (2016). Organoid culture systems for prostate epithelial tissue and prostate cancer tissue. Nat. Protoc. *11*, 347–358. https://doi.org/10.1038/nprot.2016.006.
26. Hepburn, A.C., Curry, E.L., Moad, M., Steele, R.E., Franco, O.E., Wilson, L., Singh, P., Buskin, A., Crawford, S.E., Gaughan, L., et al. (2020). Propagation of human prostate tissue from induced pluripotent stem cells. Stem Cells Transl. Med. *9*, 734–745. https://doi.org/10.1002/sctm.19-0286.
27. Brennen, W.N., Le Magnen, C., Karkampouna, S., Anselmino, N., Bock, N., Choo, N., Clark, A.K., Coleman, I.M., Dolgos, R., Ferguson, A.M., et al. (2024). Defining the challenges and opportunities for using patient-derived models in prostate cancer research. Prostate *84*, 623–635. https://doi.org/10.1002/pros.24682.
28. Cheaito, K., Bahmad, H.F., Hadadeh, O., Msheik, H., Monzer, A., Ballout, F., Dagher, C., Telvizian, T., Saheb, N., Tawil, A., et al. (2022). Establishment and characterization of prostate organoids from treatment-naïve patients with prostate cancer. Oncol. Lett. *23*, 6–16. https://doi.org/10.3892/ol.2021.13124.
29. Van Hemelryk, A., Erkens-Schulze, S., Lim, L., de Ridder, C.M.A., Stuurman, D.C., Jenster, G.W., van Royen, M.E., and van Weerden, W.M. (2023). Viability Analysis and High-Content Live-Cell Imaging for Drug Testing in Prostate Cancer Xenograft-Derived Organoids. Cells *12*, 1377. https://doi.org/10.3390/cells12101377.
30. Bigot, L., Sabio, J., Poiraudeau, L., Annereau, M., Menssouri, N., Helissey, C., Déas, O., Aglave, M., Ibrahim, T., Pobel, C., et al. (2024). Development of Novel Models of Aggressive Variants of Castration-resistant Prostate Cancer. Eur. Urol. Oncol. *7*, 527–536. https://doi.org/10.1016/j.euo.2023.10.011.
31. Chan, J.M., Zaidi, S., Love, J.R., Zhao, J.L., Setty, M., Wadosky, K.M., Gopalan, A., Choo, Z.-N., Persad, S., Choi, J., et al. (2022). Lineage plasticity in prostate cancer depends on JAK/STAT inflammatory signaling. Science *377*, 1180–1191. https://doi.org/10.1126/science.abn0478.
32. Gao, X., Wang, Y., Ribeiro, C.F., Manokaran, C., Chang, H., Von, T., Rodrigues, S., Cizmecioglu, O., Jia, S., Korpal, M., et al. (2022). Blocking PI3K p110β Attenuates Development of PTEN-Deficient Castration-Resistant Prostate Cancer. Mol. Cancer Res. *20*, 673–685. https://doi.org/10.1158/1541-7786.MCR-21-0322.
33. Gao, X., Zhao, H., Liu, J., Wang, M., Dai, Z., Hao, W., Wang, Y., Wang, X., Zhang, M., Liu, P., et al. (2024). Enzalutamide Sensitizes Castration-Resistant Prostate Cancer to Copper-Mediated Cell Death. Adv. Sci. *11*, 2401396. https://doi.org/10.1002/advs.202401396.
34. Zeng, Z., Samudio, I.J., Zhang, W., Estrov, Z., Pelicano, H., Harris, D., Frolova, O., Hail, N., Jr., Chen, W., Kornblau, S.M., et al. (2006). Simultaneous Inhibition of PDK1/AKT and Fms-Like Tyrosine Kinase 3 Signaling by a Small-Molecule KP372-1 Induces Mitochondrial Dysfunction and

Apoptosis in Acute Myelogenous Leukemia. Cancer Res. *66*, 3737–3746. https://doi.org/10.1158/0008-5472.CAN-05-1278.

35. Koul, D., Shen, R., Bergh, S., Sheng, X., Shishodia, S., Lafortune, T.A., Lu, Y., de Groot, J.F., Mills, G.B., and Yung, W.K.A. (2006). Inhibition of Akt survival pathway by a small-molecule inhibitor in human glioblastoma. Mol. Cancer Ther. *5*, 637–644. https://doi.org/10.1158/1535-7163.MCT-05-0453.

36. Mandal, M., Younes, M., Swan, E.A., Jasser, S.A., Doan, D., Yigitbasi, O., McMurphey, A., Ludwick, J., El-Naggar, A.K., Bucana, C., et al. (2006). The Akt inhibitor KP372-1 inhibits proliferation and induces apoptosis and anoikis in squamous cell carcinoma of the head and neck. Oral Oncol. *42*, 430–439. https://doi.org/10.1016/j.oraloncology.2005.09.011.

37. Lain, S., Hollick, J.J., Campbell, J., Staples, O.D., Higgins, M., Aoubala, M., McCarthy, A., Appleyard, V., Murray, K.E., Baker, L., et al. (2008). Discovery, In Vivo Activity, and Mechanism of Action of a Small-Molecule p53 Activator. Cancer Cell *13*, 454–463. https://doi.org/10.1016/j.ccr.2008.03.004.

38. Abeshouse, A., Ahn, J., Akbani, R., Ally, A., Amin, S., Andry, C.D., Annala, M., Aprikian, A., Armenia, J., Arora, A., et al. (2015). The Molecular Taxonomy of Primary Prostate Cancer. Cell *163*, 1011–1025. https://doi.org/10.1016/j.cell.2015.10.025.

39. Smith, J.C., and Sheltzer, J.M. (2022). Genome-wide identification and analysis of prognostic features in human cancers. Cell Rep. *38*, 110569. https://doi.org/10.1016/j.celrep.2022.110569.

40. Wang, S., Gao, J., Lei, Q., Rozengurt, N., Pritchard, C., Jiao, J., Thomas, G.V., Li, G., Roy-Burman, P., Nelson, P.S., et al. (2003). Prostate-specific deletion of the murine Pten tumor suppressor gene leads to metastatic prostate cancer. Cancer Cell *4*, 209–221. https://doi.org/10.1016/S1535-6108(03)00215-0.

41. Chen, Z., Trotman, L.C., Shaffer, D., Lin, H.-K., Dotan, Z.A., Niki, M., Koutcher, J.A., Scher, H.I., Ludwig, T., Gerald, W., et al. (2005). Crucial role of p53-dependent cellular senescence in suppression of Pten-deficient tumorigenesis. Nature *436*, 725–730. https://doi.org/10.1038/nature03918.

42. Limberger, T., Schlederer, M., Trachtová, K., Garces de los Fayos Alonso, I., Yang, J., Högler, S., Sternberg, C., Bystry, V., Oppelt, J., Tichý, B., et al. (2022). KMT2C methyltransferase domain regulated INK4A expression suppresses prostate cancer metastasis. Mol. Cancer *21*, 89. https://doi.org/10.1186/s12943-022-01542-8.

43. Lee, M., Kang, H., and Jang, S.-W. (2013). CoCl2 induces PC12 cells apoptosis through p53 stability and regulating UNC5B. Brain Res. Bull. *96*, 19–27. https://doi.org/10.1016/j.brainresbull.2013.04.007.

44. Dai, C., Dehm, S.M., and Sharifi, N. (2023). Targeting the Androgen Signaling Axis in Prostate Cancer. J. Clin. Orthod. *41*, 4267–4278. https://doi.org/10.1200/JCO.23.00433.

45. Thaper, D., Vahid, S., Kaur, R., Kumar, S., Nouruzi, S., Bishop, J.L., Johansson, M., and Zoubeidi, A. (2018). Galiellalactone inhibits the STAT3/AR signaling axis and suppresses Enzalutamide-resistant Prostate Cancer. Sci. Rep. *8*, 17307. https://doi.org/10.1038/s41598-018-35612-z.

46. Enzalutamide (MDV3100) | MedChemExpress. https://www.medchemexpress.com/Enzalutamide.html.

47. Choudhury, A.D. (2022). PTEN-PI3K pathway alterations in advanced prostate cancer and clinical implications. Prostate *82*, S60–S72. https://doi.org/10.1002/pros.24372.

48. Chua, C.W., Shibata, M., Lei, M., Toivanen, R., Barlow, L.J., Bergren, S.K., Badani, K.K., McKiernan, J.M., Benson, M.C., Hibshoosh, H., and Shen, M.M. (2014). Single luminal epithelial progenitors can generate prostate organoids in culture. Nat. Cell Biol. *16*, 951–954. https://doi.org/10.1038/ncb3047.

49. Waters, J.A., Urbano, I., Robinson, M., and House, C.D. (2022). Insulin-like growth factor binding protein 5: Diverse roles in cancer. Front. Oncol. *12*, 1052457. https://doi.org/10.3389/fonc.2022.1052457.

50. Ouyang, S., Mo, Z., Sun, S., Yin, K., and Lv, Y. (2020). Emerging role of Insig-1 in lipid metabolism and lipid disorders. Clin. Chim. Acta *508*, 206–212. https://doi.org/10.1016/j.cca.2020.05.042.

51. Li, X., Jiang, Z., Li, J., Yang, K., He, J., Deng, Q., Xu, S., Jiang, Z., Liu, F., and Jiang, Z. (2025). PRELP inhibits colorectal cancer progression by suppressing epithelial-mesenchymal transition and angiogenesis via the inactivation of the FGF1/PI3K/AKT pathway. Apoptosis *30*, 16–34. https://doi.org/10.1007/s10495-024-02015-7.

52. Gui, Y., Deng, X., Li, N., and Zhao, L. (2024). PRELP reduce cell stiffness and adhesion to promote the growth and metastasis of colorectal cancer cells by binding to integrin α5. Exp. Cell Res. *441*, 114151. https://doi.org/10.1016/j.yexcr.2024.114151.

53. Offermann, A., Kang, D., Watermann, C., Weingart, A., Hupe, M.C., Saraji, A., Stegmann-Frehse, J., Kruper, R., Schüle, R., Pantel, K., et al. (2021). Analysis of tripartite motif (TRIM) family gene expression in prostate cancer bone metastases. Carcinogenesis *42*, 1475–1484. https://doi.org/10.1093/carcin/bgab083.

54. Gao, W., Huang, M., Chen, X., Chen, J., Zou, Z., Li, L., Ji, K., Nie, Z., Yang, B., Wei, Z., et al. (2021). The role of S-nitrosylation of PFKM in regulation of glycolysis in ovarian cancer cells. Cell Death Dis. *12*, 408–414. https://doi.org/10.1038/s41419-021-03681-0.

55. Shao, F., Ling, L., Li, C., Huang, X., Ye, Y., Zhang, M., Huang, K., Pan, J., Chen, J., and Wang, Y. (2023). Establishing a metastasis-related diagnosis and prognosis model for lung adenocarcinoma through CRISPR library and TCGA database. J. Cancer Res. Clin. Oncol. *149*, 885–899. https://doi.org/10.1007/s00432-022-04495-z.

56. Jung, S.H., Hwang, H.J., Kang, D., Park, H.A., Lee, H.C., Jeong, D., Lee, K., Park, H.J., Ko, Y.-G., and Lee, J.-S. (2019). mTOR kinase leads to PTEN-loss-induced cellular senescence by phosphorylating p53. Oncogene *38*, 1639–1650. https://doi.org/10.1038/s41388-018-0521-8.

57. Liberzon, A., Subramanian, A., Pinchback, R., Thorvaldsdóttir, H., Tamayo, P., and Mesirov, J.P. (2011). Molecular signatures database (MSigDB) 3.0. Bioinformatics *27*, 1739–1740. https://doi.org/10.1093/bioinformatics/btr260.

58. Compound Screening Guide | MedChemExpress https://www.medchemexpress.com/literature/compound-screening-guide.html?srsltid=AfmBOopDA8qVUjkXvNkBIWv-2P7Ybe-ck05RHZh6zoTUx8fMIuG7xAiW

59. Entinostat (MS-275) |MedChemExpress. https://www.medchemexpress.com/Entinostat.html.

60. Jung, D.J., Shin, T.H., Kim, M., Sung, C.O., Jang, S.J., and Jeong, G.S. (2019). A one-stop microfluidic-based lung cancer organoid culture platform for testing drug sensitivity. Lab Chip *19*, 2854–2865. https://doi.org/10.1039/C9LC00496C.

61. Capivasertib (AZD5363) |MedChemExpress. https://www.medchemexpress.com/AZD5363.html

62. Zhang, Y., Zhang, C., Li, J., Jiang, M., Guo, S., Yang, G., Zhang, L., Wang, F., Yi, S., Wang, J., et al. (2022). Inhibition of AKT induces p53/SIRT6/PARP1-dependent parthanatos to suppress tumor growth. Cell Commun. Signal. *20*, 93. https://doi.org/10.1186/s12964-022-00897-1.

63. Buccitelli, C., and Selbach, M. (2020). mRNAs, proteins and the emerging principles of gene expression control. Nat. Rev. Genet. *21*, 630–644. https://doi.org/10.1038/s41576-020-0258-4.

64. Ahmad, F., Cherukuri, M.K., and Choyke, P.L. (2021). Metabolic reprogramming in prostate cancer. Br. J. Cancer *125*, 1185–1196. https://doi.org/10.1038/s41416-021-01435-5.

65. Pujana-Vaquerizo, M., Bozal-Basterra, L., and Carracedo, A. (2024). Metabolic adaptations in prostate cancer. Br. J. Cancer *131*, 1250–1262. https://doi.org/10.1038/s41416-024-02762-z.

66. DeBerardinis, R.J., and Chandel, N.S. (2020). We need to talk about the Warburg effect. Nat. Metab. *2*, 127–129. https://doi.org/10.1038/s42255-020-0172-2.

67. Wang, L., Wang, C., Sarwar, M.S., Chou, P., Wang, Y., Su, X., and Kong, A.-N.T. (2022). PTEN-knockout regulates metabolic rewiring and

CellPress
OPEN ACCESS

epigenetic reprogramming in prostate cancer and chemoprevention by triterpenoid ursolic acid. FASEB J. *36*, e22626. https://doi.org/10.1096/fj.202201195R.

68. Sun, J., Bok, R.A., DeLos Santos, J., Upadhyay, D., DeLos Santos, R., Agarwal, S., Van Criekinge, M., Vigneron, D.B., Aggarwal, R., Peehl, D.M., et al. (2021). Resistance to Androgen Deprivation Leads to Altered Metabolism in Human and Murine Prostate Cancer Cell and Tumor Models. Metabolites *11*, 139. https://doi.org/10.3390/metabo11030139.
69. Lin, W., Wang, K., Mo, J., Wang, L., Song, Z., Jiang, H., Wang, C., and Jin, C. (2023). PIK3R3 is upregulated in liver cancer and activates Akt signaling to control cancer growth by regulation of CDKN1C and SMC1A. Cancer Med. *12*, 14413–14425. https://doi.org/10.1002/cam4.6068.
70. Xu, W., Yu, M., Qin, J., Luo, Y., and Zhong, M. (2020). LACTB Regulates PIK3R3 to Promote Autophagy and Inhibit EMT and Proliferation Through the PI3K/AKT/mTOR Signaling Pathway in Colorectal Cancer. Cancer Manag. Res. *12*, 5181–5200. https://doi.org/10.2147/CMAR.S250661.
71. Zhou, S., Fu, H., Liu, C., Zhu, Z., Zhang, J., Weng, W., Kang, J., and Liu, Q. (2020). Value of 11C-Choline PET/CT-Based Multi-Metabolic Parameter Combination in Distinguishing Early-Stage Prostate Cancer From Benign Prostate Diseases. Front. Oncol. *10*, 600380. https://doi.org/10.3389/fonc.2020.600380.
72. Zhou, W., Shi, K., Ji, L., Wu, R., Chen, Y., Tu, H., Zhou, B., Wang, Z., and Zhang, M. (2018). Inhibition of Phospholipase D1 mRNA Expression Slows Down the Proliferation Rate of Prostate Cancer Cells That Have Transited to Androgen Independence. J. Cancer *9*, 3620–3625. https://doi.org/10.7150/jca.26689.
73. Borel, M., Cuvillier, O., Magne, D., Mebarek, S., and Brizuela, L. (2020). Increased phospholipase D activity contributes to tumorigenesis in prostate cancer cell models. Mol. Cell. Biochem. *473*, 263–279. https://doi.org/10.1007/s11010-020-03827-2.
74. Utter, M., Chakraborty, S., Goren, L., Feuser, L., Zhu, Y.-S., and Foster, D.A. (2018). Elevated phospholipase D activity in androgen-insensitive prostate cancer cells promotes both survival and metastatic phenotypes. Cancer Lett. *423*, 28–35. https://doi.org/10.1016/j.canlet.2018.03.006.
75. Yu, Y., Wang, S., Wang, Y., Zhang, Q., Zhao, L., Wang, Y., Wu, J., Han, L., Wang, J., Guo, J., et al. (2024). AKT1 Promotes Tumorigenesis and Metastasis by Directly Phosphorylating Hexokinases. J. Cell. Biochem. *125*, e30613. https://doi.org/10.1002/jcb.30613.
76. Hoxhaj, G., and Manning, B.D. (2020). The PI3K-AKT network at the interface of oncogenic signalling and cancer metabolism. Nat. Rev. Cancer *20*, 74–88. https://doi.org/10.1038/s41568-019-0216-7.
77. Papanikolaou, S., Vourda, A., Syggelos, S., and Gyftopoulos, K. (2021). Cell Plasticity and Prostate Cancer: The Role of Epithelial–Mesenchymal Transition in Tumor Progression, Invasion, Metastasis and Cancer Therapy Resistance. Cancers *13*, 2795. https://doi.org/10.3390/cancers13112795.
78. Kosuge, H., Nakakido, M., Nagatoishi, S., Fukuda, T., Bando, Y., Ohnuma, S.I., and Tsumoto, K. (2021). Proteomic identification and validation of novel interactions of the putative tumor suppressor PRELP with membrane proteins including IGFI-R and p75NTR. J. Biol. Chem. *296*, 100278. https://doi.org/10.1016/j.jbc.2021.100278.
79. Hong, R., Gu, J., Niu, G., Hu, Z., Zhang, X., Song, T., Han, S., Hong, L., and Ke, C. (2020). PRELP has prognostic value and regulates cell proliferation and migration in hepatocellular carcinoma. J. Cancer *11*, 6376–6389. https://doi.org/10.7150/jca.46309.
80. Braglia, L., Zavatti, M., Vinceti, M., Martelli, A.M., and Marmiroli, S. (2020). Deregulated PTEN/PI3K/AKT/mTOR signaling in prostate cancer: Still a potential druggable target? Biochim. Biophys. Acta. Mol. Cell Res. *1867*, 118731. https://doi.org/10.1016/j.bbamcr.2020.118731.
81. Imamura, J., Ganguly, S., Muskara, A., Liao, R.S., Nguyen, J.K., Weight, C., Wee, C.E., Gupta, S., and Mian, O.Y. (2023). Lineage plasticity and treatment resistance in prostate cancer: the intersection of genetics, epigenetics, and evolution. Front. Endocrinol. *14*, 1191311. https://doi.org/10.3389/fendo.2023.1191311.
82. Bello, T., Paindelli, C., Diaz-Gomez, L.A., Melchiorri, A., Mikos, A.G., Nelson, P.S., Dondossola, E., and Gujral, T.S. (2021). Computational modeling identifies multitargeted kinase inhibitors as effective therapies for metastatic, castration-resistant prostate cancer. Proc. Natl. Acad. Sci. USA *118*, e2103623118. https://doi.org/10.1073/pnas.2103623118.
83. Zheng, N., Wei, J., Wu, D., Xu, Y., and Guo, J. (2023). Master kinase PDK1 in tumorigenesis. Biochim. Biophys. Acta. Rev. Cancer *1878*, 188971. https://doi.org/10.1016/j.bbcan.2023.188971.
84. Takahashi, S. (2011). Downstream molecular pathways of FLT3 in the pathogenesis of acute myeloid leukemia: biology and therapeutic implications. J. Hematol. Oncol. *4*, 13. https://doi.org/10.1186/1756-8722-4-13.
85. Zhao, Y., Hu, Q., Cheng, F., Su, N., Wang, A., Zou, Y., Hu, H., Chen, X., Zhou, H.-M., Huang, X., et al. (2015). SoNar, a Highly Responsive NAD+/NADH Sensor, Allows High-Throughput Metabolic Screening of Anti-tumor Agents. Cell Metab. *21*, 777–789. https://doi.org/10.1016/j.cmet.2015.04.009.
86. Viera, T., and Patidar, P.L. (2020). DNA damage induced by KP372-1 hyperactivates PARP1 and enhances lethality of pancreatic cancer cells with PARP inhibition. Sci. Rep. *10*, 20210. https://doi.org/10.1038/s41598-020-76850-4.
87. Jiang, L., Liu, Y., Su, X., Wang, J., Zhao, Y., Tumbath, S., Kilgore, J.A., Williams, N.S., Chen, Y., Wang, X., et al. (2022). KP372-1-Induced AKT Hyperactivation Blocks DNA Repair to Synergize With PARP Inhibitor Rucaparib via Inhibiting FOXO3a/GADD45α Pathway. Front. Oncol. *12*, 976292. https://doi.org/10.3389/fonc.2022.976292.
88. Jiao, B., Liu, K., Gong, H., Ding, Z., Xu, X., Ren, J., Zhang, G., Yu, Q., and Gan, Z. (2022). Bladder cancer selective chemotherapy with potent NQO1 substrate co-loaded prodrug nanoparticles. J. Control. Release *347*, 632–648. https://doi.org/10.1016/j.jconrel.2022.05.031.
89. Guo, J., Zhu, T., Chen, L., Nishioka, T., Tsuji, T., Xiao, Z.-X.J., and Chen, C.Y. (2010). Differential Sensitization of Different Prostate Cancer Cells to Apoptosis. Genes Cancer *1*, 836–846. https://doi.org/10.1177/1947601910381645.
90. Devlies, W., Handle, F., Devos, G., Joniau, S., and Claessens, F. (2021). Preclinical Models in Prostate Cancer: Resistance to AR Targeting Therapies in Prostate Cancer. Cancers (Basel) *13*, 915. https://doi.org/10.3390/cancers13040915.
91. Macedo-Silva, C., Benedetti, R., Ciardiello, F., Cappabianca, S., Jerónimo, C., and Altucci, L. (2021). Epigenetic mechanisms underlying prostate cancer radioresistance. Clin. Epigenet. *13*, 125. https://doi.org/10.1186/s13148-021-01111-8.
92. Kumaraswamy, A., Welker Leng, K.R., Westbrook, T.C., Yates, J.A., Zhao, S.G., Evans, C.P., Feng, F.Y., Morgan, T.M., and Alumkal, J.J. (2021). Recent Advances in Epigenetic Biomarkers and Epigenetic Targeting in Prostate Cancer. Eur. Urol. *80*, 71–81. https://doi.org/10.1016/j.eururo.2021.03.005.
93. Ladds, M.J.G.W., Popova, G., Pastor-Fernández, A., Kannan, S., van Leeuwen, I.M.M., Håkansson, M., Walse, B., Tholander, F., Bhatia, R., Verma, C.S., et al. (2021). Exploitation of dihydroorotate dehydrogenase (DHODH) and p53 activation as therapeutic targets: A case study in polypharmacology. J. Biol. Chem. *295*, 17935–17949. https://doi.org/10.1074/jbc.RA119.012056.
94. Chouhan, S., Muhammad, N., Usmani, D., Khan, T.H., and Kumar, A. (2024). Molecular Sentinels: Unveiling the Role of Sirtuins in Prostate Cancer Progression. Int. J. Mol. Sci. *26*, 183. https://doi.org/10.3390/ijms26010183.
95. Cui, Y., Li, J., Zheng, F., Ouyang, Y., Chen, X., Zhang, L., Chen, Y., Wang, L., Mu, S., and Zhang, H. (2016). Effect of SIRT1 Gene on Epithelial-Mesenchymal Transition of Human Prostate Cancer PC-3 Cells. Med. Sci. Monit. *22*, 380–386. https://doi.org/10.12659/MSM.895312.

96. Ruan, L., Wang, L., Wang, X., He, M., and Yao, X. (2018). SIRT1 contributes to neuroendocrine differentiation of prostate cancer. Oncotarget *9*, 2002–2016. https://doi.org/10.18632/oncotarget.23111.

97. Guo, S., Miao, M., Wu, Y., Pan, D., Wu, Q., Kang, Z., Zeng, J., Zhong, G., Liu, C., and Wang, J. (2024). DHODH inhibition represents a therapeutic strategy and improves abiraterone treatment in castration-resistant prostate cancer. Oncogene *43*, 1399–1410. https://doi.org/10.1038/s41388-024-03005-4.

98. Zhou, Y., Tao, L., Zhou, X., Zuo, Z., Gong, J., Liu, X., Zhou, Y., Liu, C., Sang, N., Liu, H., et al. (2021). DHODH and cancer: promising prospects to be explored. Cancer Metab. *9*, 22. https://doi.org/10.1186/s40170-021-00250-z.

99. KIANI, A.K., PHEBY, D., HENEHAN, G., BROWN, R., SIEVING, P., SYKORA, P., MARKS, R., FALSINI, B., CAPODICASA, N., MIERTUS, S., et al. (2022). Ethical considerations regarding animal experimentation. J. Prev. Med. Hyg. *63*, E255–E266. https://doi.org/10.15167/2421-4248/jpmh2022.63.2S3.2768.

100. Lumibao, J.C., Okhovat, S.R., Peck, K.L., Lin, X., Lande, K., Yomtoubian, S., Ng, I., Tiriac, H., Lowy, A.M., Zou, J., and Engle, D.D. (2024). The effect of extracellular matrix on the precision medicine utility of pancreatic cancer patient–derived organoids. JCI Insight *9*, e172419. https://doi.org/10.1172/jci.insight.172419.

101. Liu, H., Gan, Z., Qin, X., Wang, Y., and Qin, J. (2024). Advances in Microfluidic Technologies in Organoid Research. Adv. Healthc. Mater. *13*, 2302686. https://doi.org/10.1002/adhm.202302686.

102. Atanasova, V.S., de Jesus Cardona, C., Hejret, V., Tiefenbacher, A., Mair, T., Tran, L., Pfneissl, J., Draganić, K., Binder, C., Kabiljo, J., et al. (2023). Mimicking Tumor Cell Heterogeneity of Colorectal Cancer in a Patient-derived Organoid-Fibroblast Model. Cell. Mol. Gastroenterol. Hepatol. *15*, 1391–1419. https://doi.org/10.1016/j.jcmgh.2023.02.014.

103. Klein, J.A., Heidmann, J.D., Kiyota, T., Fullerton, A., Homan, K.A., and Co, J.Y. (2025). The differentiation state of small intestinal organoid models influences prediction of drug-induced toxicity. Front. Cell Dev. Biol. *13*, 1508820. https://doi.org/10.3389/fcell.2025.1508820.

104. Kumar, M.S., Pester, R.E., Chen, C.Y., Lane, K., Chin, C., Lu, J., Kirsch, D.G., Golub, T.R., and Jacks, T. (2009). Dicer1 functions as a haploinsufficient tumor suppressor. Genes Dev. *23*, 2700–2704. https://doi.org/10.1101/gad.1848209.

105. Wu, X., Wu, J., Huang, J., Powell, W.C., Zhang, J., Matusik, R.J., Sangiorgi, F.O., Maxson, R.E., Sucov, H.M., and Roy-Burman, P. (2001). Generation of a prostate epithelial cell-specific Cre transgenic mouse model for tissue-specific gene ablation. Mech. Dev. *101*, 61–69. https://doi.org/10.1016/s0925-4773(00)00551-7.

106. Zheng, S., Wang, W., Aldahdooh, J., Malyutina, A., Shadbahr, T., Tanoli, Z., Pessia, A., and Tang, J. (2022). SynergyFinder Plus: Toward Better Interpretation and Annotation of Drug Combination Screening Datasets. Genom. Proteom. Bioinform. *20*, 587–596. https://doi.org/10.1016/j.gpb.2022.01.004.

107. Bankhead, P., Loughrey, M.B., Fernández, J.A., Dombrowski, Y., McArt, D.G., Dunne, P.D., McQuaid, S., Gray, R.T., Murray, L.J., Coleman, H.G., et al. (2017). QuPath: Open source software for digital pathology image analysis. Sci. Rep. *7*, 16878. https://doi.org/10.1038/s41598-017-17204-5.

108. Love, M.I., Huber, W., and Anders, S. (2014). Moderated estimation of fold change and dispersion for RNA-seq data with DESeq2. Genome Biol. *15*, 550. https://doi.org/10.1186/s13059-014-0550-8.

109. Wickham, H. (2016). ggplot2 (Springer International Publishing). https://doi.org/10.1007/978-3-319-24277-4.

110. Ulgen, E., Ozisik, O., and Sezerman, O.U. (2019). pathfindR: An R Package for Comprehensive Identification of Enriched Pathways in Omics Data Through Active Subnetworks. Front. Genet. *10*, 858. https://doi.org/10.3389/fgene.2019.00858.

111. Xie, Z., Bailey, A., Kuleshov, M.V., Clarke, D.J.B., Evangelista, J.E., Jenkins, S.L., Lachmann, A., Wojciechowicz, M.L., Kropiwnicki, E., Jagodnik, K.M., et al. (2021). Gene Set Knowledge Discovery with Enrichr. Curr. Protoc. *1*, e90. https://doi.org/10.1002/cpz1.90.

112. Milacic, M., Beavers, D., Conley, P., Gong, C., Gillespie, M., Griss, J., Haw, R., Jassal, B., Matthews, L., May, B., et al. (2024). The Reactome Pathway Knowledgebase 2024. Nucleic Acids Res. *52*, D672–D678. https://doi.org/10.1093/nar/gkad1025.

113. Szklarczyk, D., Kirsch, R., Koutrouli, M., Nastou, K., Mehryary, F., Hachilif, R., Gable, A.L., Fang, T., Doncheva, N.T., Pyysalo, S., et al. (2023). The STRING database in 2023: protein-protein association networks and functional enrichment analyses for any sequenced genome of interest. Nucleic Acids Res. *51*, D638–D646. https://doi.org/10.1093/nar/gkac1000.

114. Subramanian, A., Tamayo, P., Mootha, V.K., Mukherjee, S., Ebert, B.L., Gillette, M.A., Paulovich, A., Pomeroy, S.L., Golub, T.R., Lander, E.S., and Mesirov, J.P. (2005). Gene set enrichment analysis: A knowledge-based approach for interpreting genome-wide expression profiles. Proc. Natl. Acad. Sci. USA *102*, 15545–15550. https://doi.org/10.1073/pnas.0506580102.

115. Tang, D., Chen, M., Huang, X., Zhang, G., Zeng, L., Zhang, G., Wu, S., and Wang, Y. (2023). SRplot: A free online platform for data visualization and graphing. PLoS One *18*, e0294236. https://doi.org/10.1371/journal.pone.0294236.

CellPress
OPEN ACCESS

STAR★METHODS

KEY RESOURCES TABLE

REAGENT or RESOURCE	SOURCE	IDENTIFIER
**Antibodies**		
Rabbit monoclonal anti-PTEN (138G6)	Cell Signaling Technology	Cat#9559; RRID:AB_390810
Rabbit monoclonal anti-STAT3 (D3Z2G)	Cell Signaling Technology	Cat#12640; RRID:AB_2629499
Mouse monoclonal anti-TP53 (1C12)	Cell Signaling Technology	Cat#2524; RRID:AB_331743
Mouse monoclonal anti-β-ACTIN	Proteintech	Cat#66009-1-Ig; RRID:AB_2687938
Rabbit monoclonal anti-AKT (Pan) (C67E7)	Cell Signaling Technology	Cat#4691; RRID:AB_915783
Rabbit polyclonal anti-pospho-AKT (Ser473)	Cell Signaling Technology	Cat#9271; RRID:AB_329825
Rabbit monoclonal anti-KI67 (D3B5)	Cell Signaling Technology	Cat#9129; RRID:AB_2687446
Rabbit monoclonal anti-AR (D6F11)	Cell Signaling Technology	Cat#5153; RRID:AB_10691711
Rabbit monoclonal anti-AR [(EPR1535(2)]	Abcam	Cat#ab133273; RRID:AB_11156085
Rabbit monoclonal anti-CK8 (EP1628Y)	Abcam	Cat#ab53280; RRID:AB_869901
Mouse monoclonal anti-P63 (4A4)	Abcam	Cat#ab735; RRID:AB_305870
**Bacterial and virus strains**		
MSCV CreERT2 puro	Kumar et al. (2009)[104]	Addgene #22776; RRID:Addgene_22776
**Biological samples**		
Murine healthy prostate or prostate tumor	This study	N/A
**Chemicals, peptides, and recombinant proteins**		
Geltrex™ LDEV-Free Reduced Growth Factor Basement Membrane Matrix	Gibco	Cat#A1413202
Cell Recovery Solution	Corning	Cat#354253
Matrigel® Growth Factor Reduced (GFR) Basement Membrane Matrix	Corning	Cat#356231
PDPK1/AKT/FLT dual pathway inhibitor (DPI)	Santa Cruz Biotechnology	Cat#CAS 331253-86-2
Tenovin-6 Hydrochloride (T6)	MedChemExpress	Cat#HY-15510B
Human Plasma-Like Medium (HPLM)	Gibco	Cat#A4899101
B-27™ Supplement	Gibco	Cat#17504001
Nicotinamide	Sigma-Aldrich	Cat#N0636
N-acetyl-L-cysteine	Sigma-Aldrich	Cat#A9165
4,5α-Dihydrotestosterone	Sigma-Aldrich	Cat#a8380
A 83-01	Sigma-Aldrich	Cat#SML0788
Human EGF Recombinant protein	Gibco	Cat#AF-100-15
Y-27632 dihydrochloride	MedChemExpress	Cat# HY-10583
Capivasertib	MedChemExpress	Cat# HY-15431
Enzalutamide	MedChemExpress	Cat#HY-70002
**Critical commercial assays**		
RealTime-Glo™ MT Cell Viability Assay	Promega	Cat#G9711
CellTiter-Glo® 3D Cell Viability Assay	Promega	Cat#G9681
**Deposited data**		
Human PCa RNA Seq data	Abeshouse et al. (2015)[38]	TCGA (Accession number phs000178)
Raw and analyzed data	This study	Gene Expression Omnibus: GSE291912
Mouse reference genome GRCm38.101	Genome Reference Consortium	https://www.ncbi.nlm.nih.gov/datasets/genome/GCF_000001635.20/
**Experimental models: Cell lines**		
Human 22RV1 primary PCa cell line	ATCC	CRL-2505; RRID:CVCL_1045

(*Continued on next page*)

Continued		
REAGENT or RESOURCE	SOURCE	IDENTIFIER
Human LNCaP metastatic PCa cell line	ATCC	CRL-1740; RRID:CVCL_A4BQ
Human DU145 metastatic PCa cell line	ATCC	HTB-81, RRID:CVCL_0105
Human PC3 metastatic PCa cell line	ATCC	CRL-1435; RRID:CVCL_0035
Experimental models: Organisms/strains		
Pten$^{loxP/loxP}$PB-Cre4^{+}	Laboratory of Prof. Lukas Kenner	Wu et al. (2001)[105] Wang et al. (2003)[40]
Pten$^{loxP/loxP}$*Stat3*$^{loxP/loxP}$PB-Cre4^{+}	Laboratory of Prof. Lukas Kenner	Pencik et al. (2015)[16]
Pten$^{loxP/loxP}$*Trp53*$^{loxP/loxP}$PB-Cre4^{+}	Laboratory of Prof. Lukas Kenner	Chen et al. (2005)[41]
Oligonucleotides		
Genotyping PCR primers	This study	Table S1
qRT-PCR primers	This study	Table S1
Software and algorithms		
GraphPad Prism 8.0.2	GraphPad Software	https://www.graphpad.com/
SynergyFinderplus	Zheng et al. (2022)[106]	https://synergyfinder.org/#!/
Survival analysis online tool	Smith and Sheltzer (2022)[39]	https://tcga-survival.com/
QuPath 0.4.4	Bankhead et al. (2017)[107]	https://qupath.github.io/

EXPERIMENTAL MODEL AND STUDY PARTICIPANT DETAILS

Animal models

All mice were maintained on a C57Bl/6-Sv/129 mixed background under specific pathogen-free conditions at 20°C–24°C. Previously described PCa mouse models with *loxP* sites for *Pten*, *Pten/Stat3*, and *Pten/Tp53* were bred with PB-Cre4 mice[105] to obtain mice with a prostate-specific deletion of respective genes. Tumoroids and organoids were either derived from tumors of the *Pten*$^{loxP/loxP}$PB-Cre4^{+} (*Pten* KO),[40] *Pten*$^{loxP/loxP}$*Stat3*$^{loxP/loxP}$PB-Cre4^{+} (*Pten/Stat3* dKO),[16] and *Pten*$^{loxP/loxP}$*Trp53*$^{loxP/loxP}$PB-Cre4^{+} (*Pten/Tp53* dKO)[41] mouse models, or from healthy prostates from *Pten*$^{loxP/loxP}$, *Pten*$^{loxP/loxP}$*Stat3*$^{loxP/loxP}$, or *Pten*$^{loxP/loxP}$*Trp53*$^{loxP/loxP}$ mice (WT), respectively. Male animals of all genotypes were sacrificed at 19 weeks of age and the prostate/tumor tissues were isolated. Only anterior and dorsal lobes were used. Tissue was partly embedded in paraffin or used for organoid/tumoroid generation. All animal experiments were reviewed and approved by the Federal Ministry for Education, Science and Research of the Republic of Austria and conducted according to regulatory and animal well-fare standards (BMWF-66.009/0281-I/3b/2012, BMBWF GZ 66.009/0135-WF/V/3b/2016).

Murine PCa organoids and tumoroids

Organoids and tumoroids from murine healthy prostates or prostate tumors, respectively, were isolated and cultured as previously described.[23,48] Briefly, isolated murine tissues were mechanically and enzymatically (Collagenase B, 5mg/ml, 1h 37°C) digested into single cells, filtered, washed, and the cell pellet was resuspended in Matrigel^{R} (Corning #356234) or Geltrex (Gibco #A1413202) and plated as hanging drops. After polymerisation of the matrix at 37°C, culture medium was added. Culture medium: Basal medium (adDMEM, 1% GlutaMAX, 1% HEPES, 1% PenStrep) plus B27 (1x), Nicotinamide (10mM), N-acetylcystein (1.25mM), Dihydrotestosterone (1nM), A83-01 (200nM), Y-27632 (10μM), and EGF (50ng/ml). Organoids and tumoroids were passaged every 5–7 days according to their size and growth rate (0.1% Trypsin, 27G needle, reseed at ~10 000 cells/15μL ECM). For all experiments organoids/tumoroids below passage 35 were used.

Human PCa cell lines

All human PCa cell lines were obtained from ATCC and cultured at 37°C with 5% CO2. The human PCa cell lines 22RV1, PC3, and DU145 were cultured in human plasma like medium (HPLM, Gibco #A4899101), while the PCa cell line LNCaP was cultured in RPMI (Gibco #11875085) supplemented with 10% FCS and 1% PenStrep.

METHOD DETAILS

Analysis of human publicly available data

Data for the Kaplan-Meier survival curves was extracted from the publicly available TCGA PRAD PCa datasets for RNA sequencing and genome/exome sequencing using an online tool.[39] Survival curves and statistics were performed using GraphPad Prism 8.0.2 (Mantel-Cox test).

Lentiviral transduction

For lentiviral transduction, organoid-derived single cells were seeded on 2D tissue culture plates 48h before adding lentiviral particles either generated from the MSCV CreERT2 puro vector (Addgene plasmid # 22776) or the control plasmid MSCV CreCut puro, that was created by shortening the sequence of the Cre-recombinase to make it non-functional. After 48h, transduced cells were plated as 3D cultures in ECM domes, and after 24h selection medium (culture medium +3.5μg/ml puromycin) was added. Cre-recombinase or CreCut expression was confirmed by PCR (Table S1). After recovery, the KO of the specific genes was induced by adding 500nM 4-hydroxytamoxifen. Final control organoid or KO tumoroid lines were generated from single-cell clones. For this, organoid-derived single cells were seeded sparsely into ECM domes and grown for three days. To dissolve the ECM, Cell Recovery Solution (Corning #354253) was added, and using a microscope and pipette single organoids were transferred to fresh ECM domes for expansion.

Genotyping PCR

DNA was isolated from snap frozen organoid/tumoroid pellets according to the manufacturer's protocol (Quiagen, DNeasy Blood & Tissue Kit #69504). PCR was performed to confirm the genotype of all used organoids and tumoroids using GoTaq DNA Polymerase (Promega #M3001) and primers specific for targets of interest (Table S1). Thermal cycling conditions were optimised for each primer pair and PCR products were visualised using gel electrophoresis.

qRT-PCR

Isolated RNA was transcribed to cDNA according to the manufacturer's protocol (Procomcure Biotech # PCCSKU1301). qRT-PCR was performed using Luna Universal qPCR Master Mix (NEB #M3003S) and primers specific for targets of interest (Table S1). Delta-CT values were calculated based on the housekeeping gene *β-Actin* and data was analyzed further using GraphPad Prism 8.0.2.

Western blotting

Proteins were isolated from snap-frozen organoid/tumoroid pellets. To induce TP53 expression, organoids/tumoroids were treated with 100μM $CoCl_2$ overnight. Pellets were resuspended in Hunt buffer (20 mM Tris pH 8, 100 mM NaCl, 1 mM EDTA, 0.5% NP-40, protease inhibitor, Roche), frozen in liquid nitrogen, thawed at 37°C, frozen in liquid nitrogen, and centrifuged for 30min at 15 000g. The supernatant containing the proteins was collected, and protein concentration was measured using Bradford assay. For all blots 10μg of protein per sample was used. Samples were diluted with 4x Laemmli buffer (Bio-Rad Cat#1610747), heat inactivated at 95°C for 8min, and loaded onto 10% SDS-PAGE gels. Gels were run at 100V and transferred to nitrocellulose membranes (wet transfer, 120V 2h). Membranes were blocked with 5% milk powder or 5% BSA in TBST and incubated with the primary antibodies at 4°C overnight (Table S2). Then membranes were washed, incubated with HRP-conjugated secondary antibodies (1:10 000, 1h RT), and the signal was developed using chemiluminescent solution ECL (Cytiva Amersham ECL #RPN2232) and measured using ChemiDoc XRS+ (Bio-Rad). Quantification of blots was done using ImageLab software 6.1, and statistical analysis was performed using GraphPad Prism 8.0.2 (One-way ANOVA, Tukey's test).

Proliferation assay

Organoid/tumoroid-derived single cells were seeded at a density of 3000 cells/9μL Geltrex in a 96-well plate. Medium containing RealTime-Glo (Promega #G9711) was added after ECM polymerisation and refreshed on day three. Luminescence signal was measured every 24h for six days. Data was normalised to signal from day one to calculate the proliferation rate. Statistical analysis was performed using GraphPad Prism 8.0.2 (One-way ANOVA, Tukey's test).

Immunohistochemistry

Organoid/tumoroid-derived single cells were grown for seven days at a density of 10,000 cells/15μL Matrigel^R or Geltrex. After fixation with 4% paraformaldehyde, organoids/tumoroids were washed and resuspended in agarose domes (0.8% in PBS), which were dehydrated and embedded in paraffin. Both murine tissue and embedded 3D lines were cut into 2μm thick sections and stained with haematoxylin and eosin (HE), or with antibodies against proteins of interest (Table S2). Signal was developed using AEC substrate (BD Pharmingen #551015) or ImmPACT DAB EqV Substrate (Vector laboratories #SK-4103) and slides were scanned for further analysis by pathologists trained in uropathology. Quantification of stainings was done using QuPath 0.4.4 on manually selected healthy prostate glands or tumors (excluding stromal cells) and organoids/tumoroids.

RNA sequencing and gene expression analysis

Organoid/tumoroid-derived single cells were seeded at a density of 10,000 cells/15μL Geltrex. After seven days, ECM domes were dissolved in lysis buffer (Qiagen RNeasy Kit #74104) and samples were stored at −80°C. RNA was isolated according to the manufacturer's protocol. The RNA of all generated *in vivo* and *in vitro* organoids and tumoroids was sent to Lexogen GmbH for bulk-RNA sequencing (Illumina shared lane, 100M total reads). Results were mapped to mouse genome GRCm38.101 using STAR aligner and quality control was performed with the RSEQC Quality control package (Python). Changes in gene expression were analyzed using DESeq2.[108] Genes with an adjusted *p*-value <0.05 and an absolute Log2fold >0 between groups were considered significant. Data visualisation, including volcano plots and heatmaps, was done using ggplot2.[109] Biological processes were inferred through pathway enrichment analysis using the pathfindR package.[110] Significant DEGs were further analyzed by Enrichr,[111] Reactome,[112] and

STRING database 12.0.[113] For generating STRING networks a maximum of 200 DEGs were used. All analyses were conducted using R version 4.3.1. Gene set enrichment analysis was performed by mapping significantly differentially expressed genes to the mouse-orthologue hallmark gene sets using Mouse MSigDB v2024.1.Mm.[114] Additional plots were generated using SRPlot.[115]

Medium-throughput compound screen

All screened compounds were obtained from commercial sources within the PLACEBO in-house collection at the Center for Molecular Medicine (CeMM) of the Austrian Academy of Sciences, Vienna. Compounds were dispensed into 384-well plates (Corning #3701, #3764) as nanodroplets using an acoustic dispensing system (Echo 550 BeckmanCoulter). *In vivo* tumoroid-derived single cells were grown for two days before isolating small tumoroids using Cell Recovery Solution (Corning #354253). Using an automatic multichannel pipette, 1000 tumoroids were seeded in suspension in 50µL medium supplemented with 5% Geltrex per well. For the initial screening, 388 compounds were tested at a concentration of 10µM. As a follow-up, 8-point dose-response curves in a 3-fold dilution series were performed for eight selected compounds following the same protocol. After 48h, viability was measured using CellTiter-Glo 3D Cell Viability Assay (Promega #G9681) and luminescence was measured using the Envision (Revvity) plate reader. DMSO and 10µM bortezomib were used as negative and positive controls, respectively. "Hits" were defined as compounds leading to more than 50% signal inhibition compared to DMSO controls (percentage of control: POC value). Negative POC values are the result of tested compounds showing a higher inhibitory effect than the positive control bortezomib. Initial data analysis of luminescence readouts was performed using Biovia PipelinePilot (Dassault Systems) and Spotfire Analyst (TIBCO) software. Further data analysis and visualisation was performed using the ComplexHeatmap and ggplot2 packages in R. Curve fitting for IC50 calculation and statistical analysis was performed using GraphPad Prism 8.0.2.

Validation of compounds on murine tumoroids

Dose-response curves for capivasertib (MedChemExpress # HY-15431), the PDPK1/AKT/FLT dual pathway inhibitor (DPI) (SantaCruz Biotechnology #CAS 331253-86-2) and tenovin-6 (T6) (MedChemExpress #1011301-29-3) were performed on *in vivo* and *in vitro* organoids and tumoroids. Organoid/tumoroid-derived single cells were seeded in 9µL Geltrex domes at a density of 2000 cells onto 96-well plates and grown for two days, before adding the compounds in a 3-fold dilution series. After 48h, CellTiter-Glo 3D Cell Viability Assay (Promega #G9681) was added to assess cell viability. Half-maximal inhibitory concentration (IC50) was calculated based on negative (max. 0.27% DMSO) and positive (30% DMSO) controls using GraphPad Prism 8.0.2.

Validation of compounds on human cell lines

For IC50 calculations, 4000 cells per human PCa cell line (22RV1, LNCaP, DU145, PC3) were seeded in HPLM per well onto 96-well plates. After cell attachment, the compounds (DPI and T6) or enzalutamide (MedChemExpress #HY-70002) at specified concentrations were added, and Bemcentinib (10 µM) or 30% DMSO were used as positive controls. Cell viability was determined after 48h by adding Resazurin (Sigma Aldrich #B70717) diluted 1:5 in HPLM. After incubation for 2h at 37°C the fluorescence signal was measured (excitation 530/570 nm, emission 580/620nm). Curve fitting and statistical analysis was performed using GraphPad Prism 8.0.2.

Synergy assay

For synergy experiments, LNCaP cells were seeded onto 96-well plates at a density of 4000 cells per well in HPLM. After 24h, enzalutamide together with either DPI or T6 were added at specified concentrations. After 48h, cell viability was measured using Resazurin. Data was analyzed based on the highest single agent (HSA) synergy model using SynergyFinderplus.[106]

QUANTIFICATION AND STATISTICAL ANALYSIS

All statistical analysis was performed using GraphPad Prism 8.0.2. Specific statistical tests and size of groups (N) are mentioned in respective method section and figure legends. 95% confidence interval: ns $p > 0.05$; *$p \leq 0.05$; **$p \leq 0.01$; ***$p \leq 0.001$; ****$p \leq 0.0001$.

Cell Reports Methods, Volume *6*

Supplemental information

Biobank of genetically defined murine prostate cancer tumoroids uncovers oncogenic pathways and drug vulnerabilities driven by PTEN-loss

Jessica Kalla, Thomas Dillinger, Zlata Pavlovicova, Reema Jacob, Emine Atas, Katarina Mišura, Anil Baskan, Kristina Draganić, Andreas Tiefenbacher, Tanja Limberger, Theresia Mair, Gabriel Wasinger, Ludovica Villanti, Stefan Kubicek, Lukas Kenner, and Gerda Egger

SUPPLEMENTARY FIGURES AND FIGURE LEGENDS

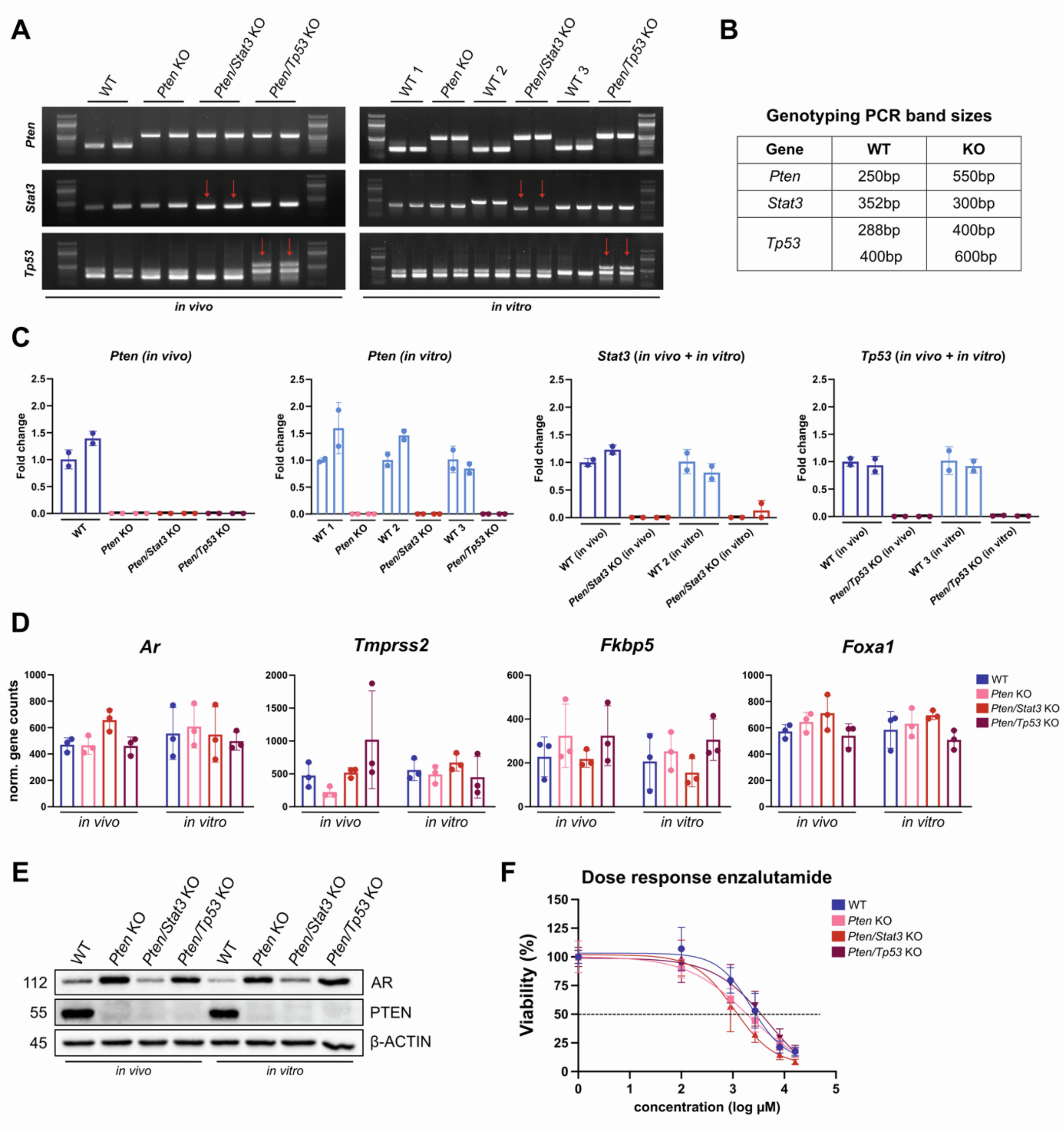

Figure S1. Confirmation of deletion of genes of interest on DNA and RNA level, and androgen status of organoid/tumoroid models, Related to Figure 1.

(A) Representative images of gel electrophoresis for genotyping using genomic DNA of *in vivo* (left) and *in vitro* (right) organoids and tumoroids for *Pten* (top), *Stat3* (middle), and *Tp53* (bottom). Bands were visualised using UV light. Arrows in middle and lower panels point to KO-specific bands (N=2). **(B)** Table summarising band sizes shown in (A) for WT or KO sequences for *Pten*, *Stat3*, and *Tp53*. **(C)** Representative gene expression analysis for *Pten*, *Stat3*, and *Tp53* of indicated organoid and tumoroid lines quantified by qRT-PCR. Expression levels are shown relative to *β-Actin* as housekeeping gene and normalised to one WT organoid line. Bars represent the mean ± SD of technical duplicates. **(D)** Bar graph showing normalised counts of androgen receptor (*Ar*), AR target genes *Tmprss2* and *Fkbp5*, and AR co-factor *Foxa1* across different models and genotypes. Bars represent the mean ± SD of biological triplicates. Normalisation and transformation were performed using DESeq2 to account for library size differences. **(E)** Western blot analysis of representative murine *in vivo* and *in vitro* organoids and KO tumoroids for indicated genotypes for AR, PTEN, and β-ACTIN as loading control. **(F)** Dose-response curves for enzalutamide on *in vivo* WT organoids and KO tumoroids (WT: 22.86μM, *Pten* KO: 18.28μM, *Pten/Stat3* KO: 10.86μM, *Pten/Tp53* KO: 48.19μM). Points represent means and ± SD of technical triplicates (N=3). Curve fitting was performed using GraphPad Prism 8.0.2.

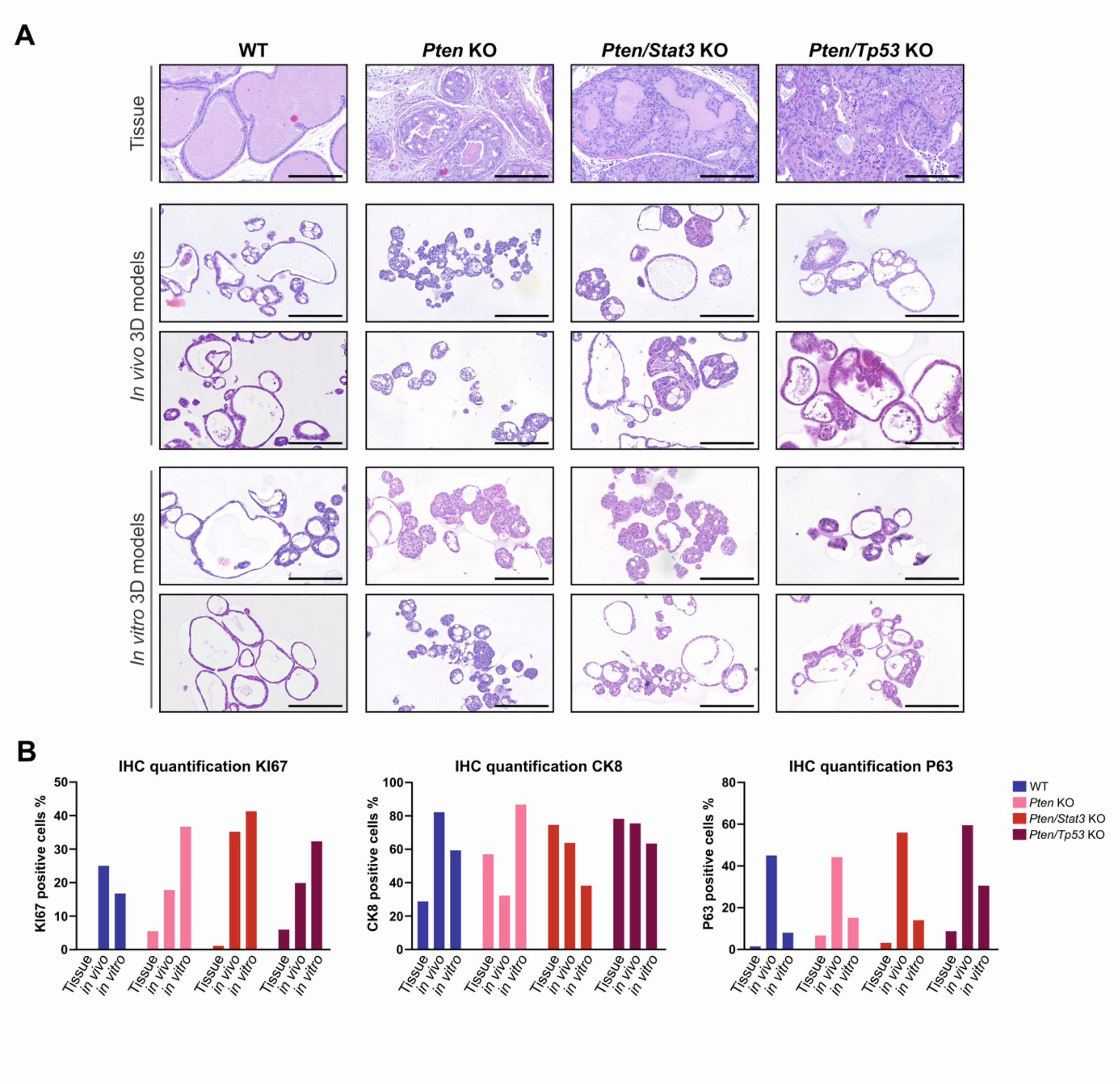

Figure S2. Morphology and marker expression with quantification of tissue and organoid/tumoroids lines, Related to Figure 2.

(A) Representative images of murine WT and tumour tissues and *in vivo* and *in vitro* organoid and tumoroid models stained with haematoxylin and eosin (HE). Stainings show biological replicates for tissues and organoids/tumoroids in addition to samples shown in Figure 2. Scale bar 200µm. (**B)** Quantification of protein expression of immunohistochemistry stainings from Figure 2 for KI67, CK8, and P63. Bars represent percentages of positive cells (nucleus or cytoplasm) for each representative tissue/organoid/tumoroid. Quantification was performed in QuPath 0.4.4 and data was visualised with GraphPad Prism 8.0.2.

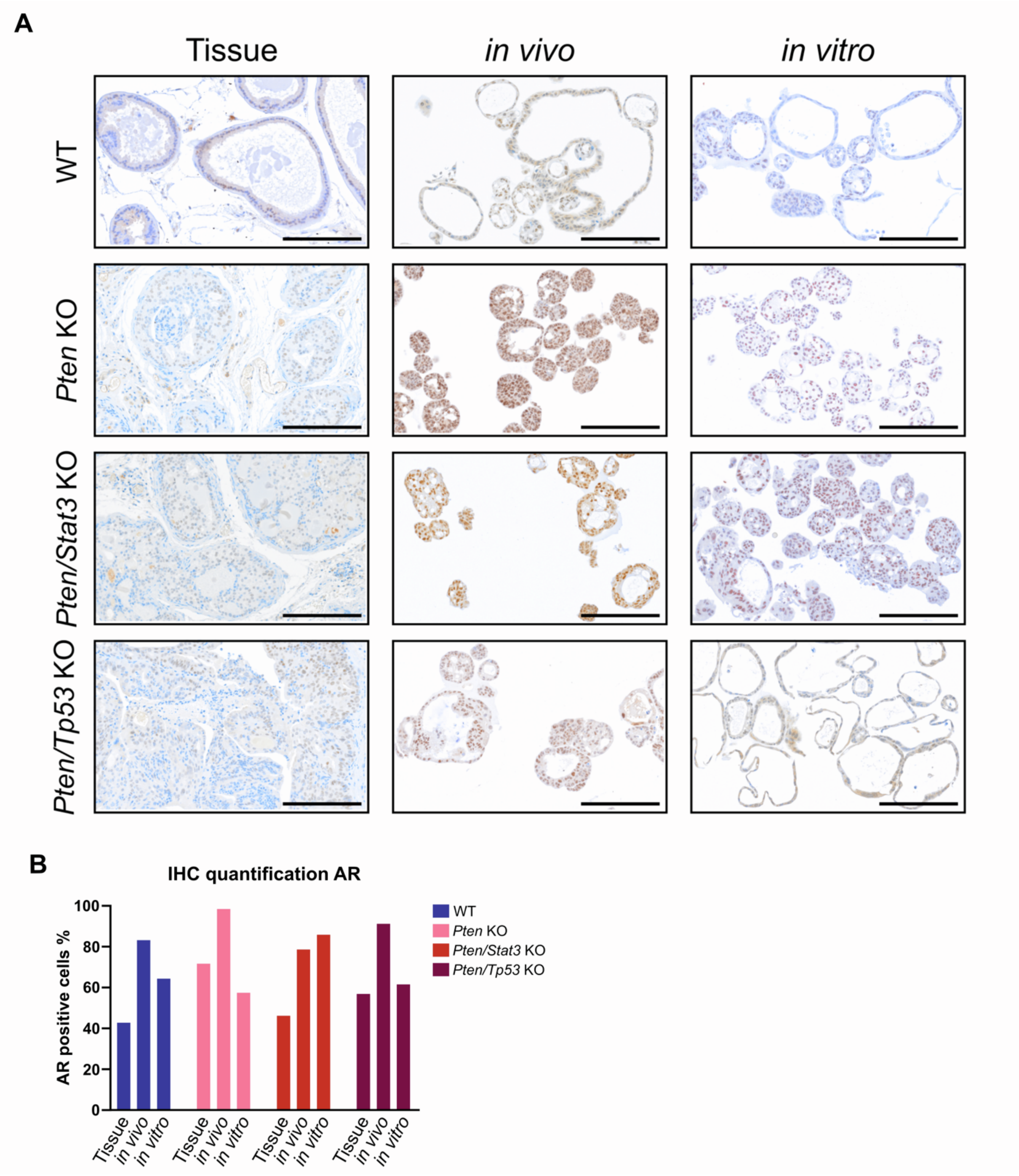

Figure S3. AR expression with quantification of tissue and organoid/tumoroids lines, Related to Figure 2.

(A) Immunohistological comparison between representative prostate tissues/tumours and organoids/tumoroids of all genotypes stained for androgen receptor (AR). Same samples as in Figure S1D-E are shown. Scale bar 200µM. **(B)** Quantification of nuclear AR expression shown in A. performed as described in B.

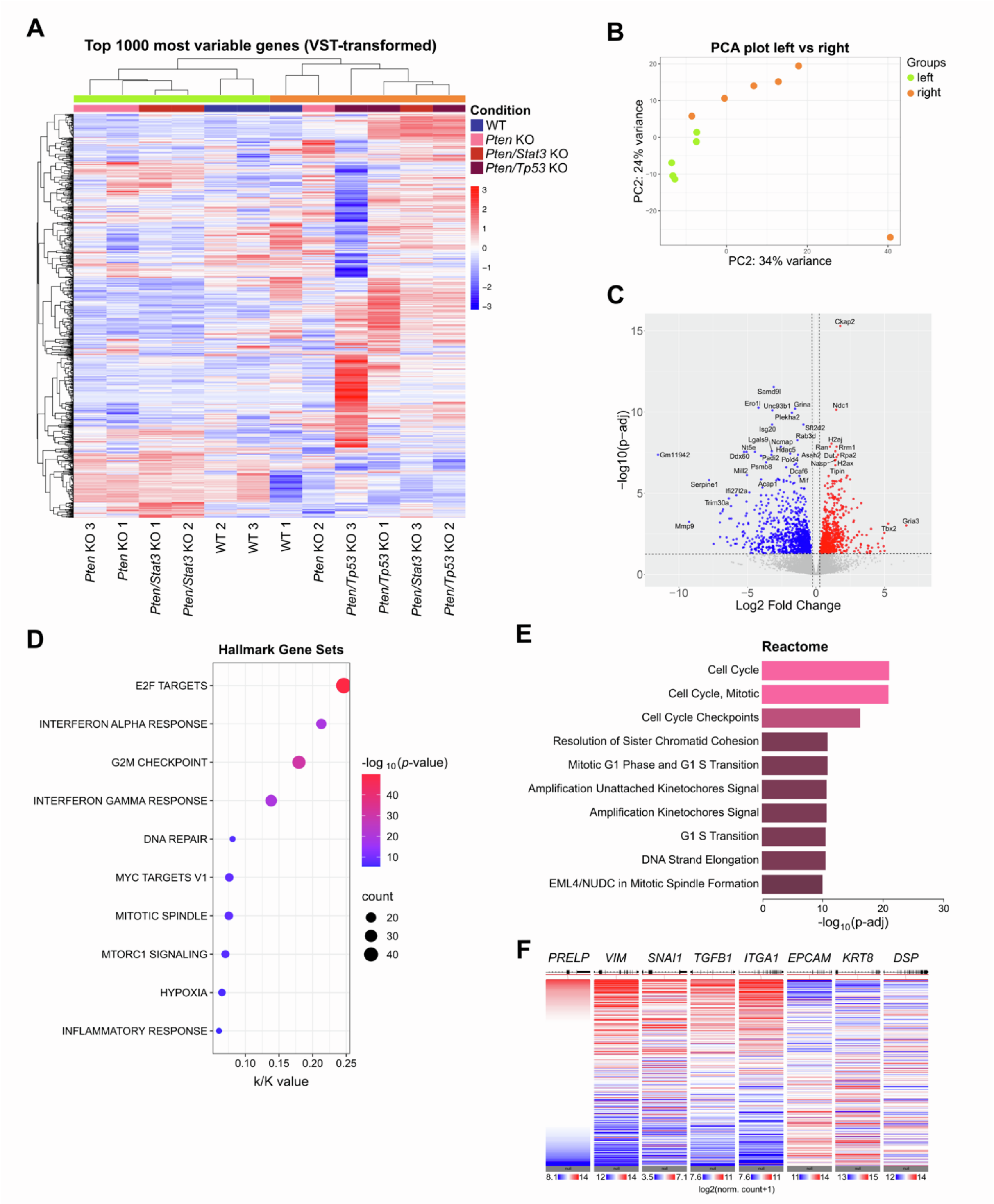

Figure S4. Organoid and tumoroid lines show different transcriptomes based on cell cycle regulation, Related to Figure 3.

(A) Dendrogram and heatmap showing unsupervised hierarchical clustering of the top 1000 most variable genes for all *in vivo* organoid and tumoroid lines based on VST-normalised gene counts as shown in Figure 3D, colour-coded for the two main branches of the dendrogram in green (left) and orange (right). **(B)** Principal component analysis (PCA) of bulk RNA sequencing data of *in vivo* WT organoid and indicated KO tumoroid lines (N=3). Samples are grouped based on clustering in the heatmap in (A). **(C)** Volcano plot depicting differentially expressed genes (DEGs) for *in vivo* WT organoid and KO tumoroid lines grouped as in (A,B). Genes with p-adj < 0.05 and Log2fold > 0 (red) or < 0 (blue) are highlighted. **(D)** Bubble plot showing the top 10 enriched hallmark gene sets identified by MSigDB gene set enrichment analysis of DEGs as in (C). Size of points reflects number of DEGs mapped to specific pathways, while colour reflects statistical significance (-log10 *p*-value). k/K value describes the

ratio of number of genes in input list (k) divided by the number of total genes in the gene set of the database (K). (**E)** Bar graph showing the top 10 enriched Reactome pathways (Enrichr) of DEGs as in (C) sorted by statistical significance (-log10 p-adj). (**F)** XENA browser analysis[1] showing gene expression analysis based on the TCGA-PRAD RNA sequencing data set[2] for *Prelp* and its correlation with mesenchymal genes *VIM*, *SNAI1*, *TGFβ1*, and *ITGA1*, and negative correlation with epithelial genes *EPCAM*, *KRT8*, and *DSP*. Each line represents one patient with gene expression indicated as log2 (norm. count +1, red: upregulation, blue: downregulation).

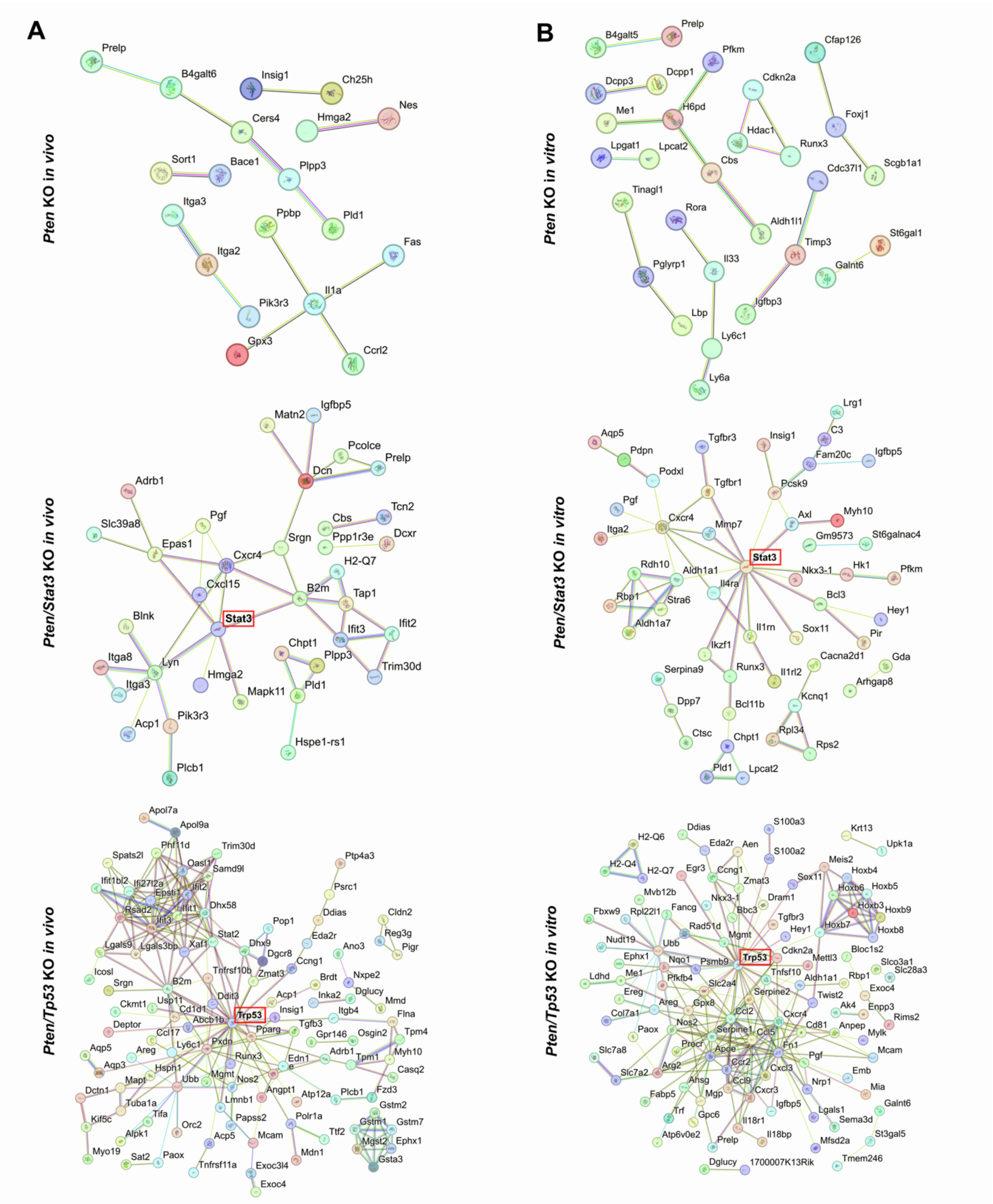

Figure S5. Differentially expressed genes extracted from in vivo and in vitro tumoroid vs organoid comparisons form functional clusters, Related to Figure 4.

Protein-protein interaction networks based on significant differentially expressed genes (DEGs) for *in vivo* (**A**) and *in vitro* (**B**) *Pten* KO (top), *Pten/Stat3* KO (middle), and *Pten/Tp53* KO (bottom) tumoroids compared to WT organoids constructed using the STRING database. For *Pten* KO and *Pten/Stat3* KO tumoroids all DEGs were used, while for *Pten/Tp53* KO tumoroids the top 200 DEGs were used. *Stat3* and *Tp53* are highlighted in red.

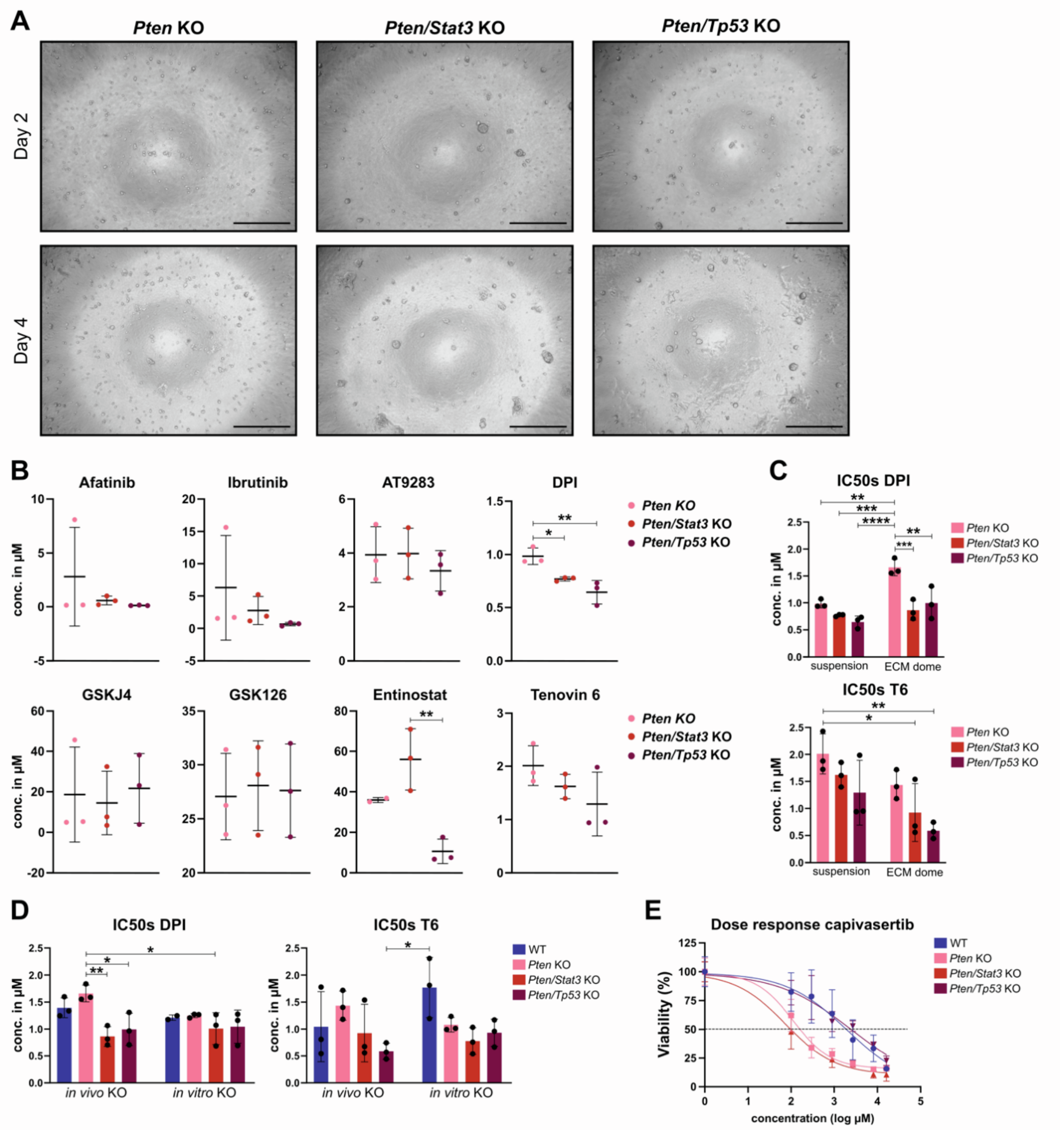

Figure S6. Dose-response of selected compounds and survival analysis of PDPK1/AKT/FLT dual pathway inhibitor (DPI) and tenovin-6 (T6) targets, Related to Figure 6.

(A) Representative bright-field microscopic images of untreated control tumoroids seeded in 384-well plates for dose-response screens. Day 2 corresponds to the first day under drug treatment, after tumoroids were seeded as single cells on Day 0. Scalebar 500µM. (**B)** Dot plots showing the mean ± SD of the half-maximal inhibitory concentrations (IC50) for eight compounds selected from the medium-throughput drug screen (related to Fig. 6E) screened in suspension (N=3). Statistical analysis was performed using GraphPad Prism 8.0.2 (One-way ANOVA, Tukey´s test). $p > 0.05$ if not specified otherwise, *$p \leq 0.05$; **$p \leq 0.01$. **(C)** Bar graphs showing means and ± SD of IC50 values for the DPI (top) and T6 (bottom) either measured in suspension or in extracellular matrix (ECM) domes for all *in vivo* KO tumoroids (N=3). Statistical analysis was performed using GraphPad Prism 8.0.2 (One-way ANOVA, Tukey´s test). $p > 0.05$ if not specified otherwise, *$p \leq 0.05$, **$p \leq 0.01$, ***$p \leq 0.001$, ****$p \leq 0.0001$. **(D)** Bar graphs showing means and ± SD of IC50 as shown in Figure 7D for DPI (left) and T6 (right) for *in vivo* and *in vitro* WT organoid and tumoroid lines of all genotypes (N=3). Statistical analysis was performed using GraphPad Prism 8.0.2 (One-way ANOVA, Tukey´s test). $p > 0.05$ if not specified otherwise, *$p \leq 0.05$. **(E)** Dose-response curves for capivasertib on *in vivo* WT organoids and KO tumoroids (WT: 19.82µM, *Pten* KO: 1.12µM, *Pten/Stat3* KO: 0.74µM, *Pten/Tp53* KO: 25.59µM). Points represent means and ± SD of technical triplicates (N=3). Curve fitting was performed using GraphPad Prism 8.0.2.

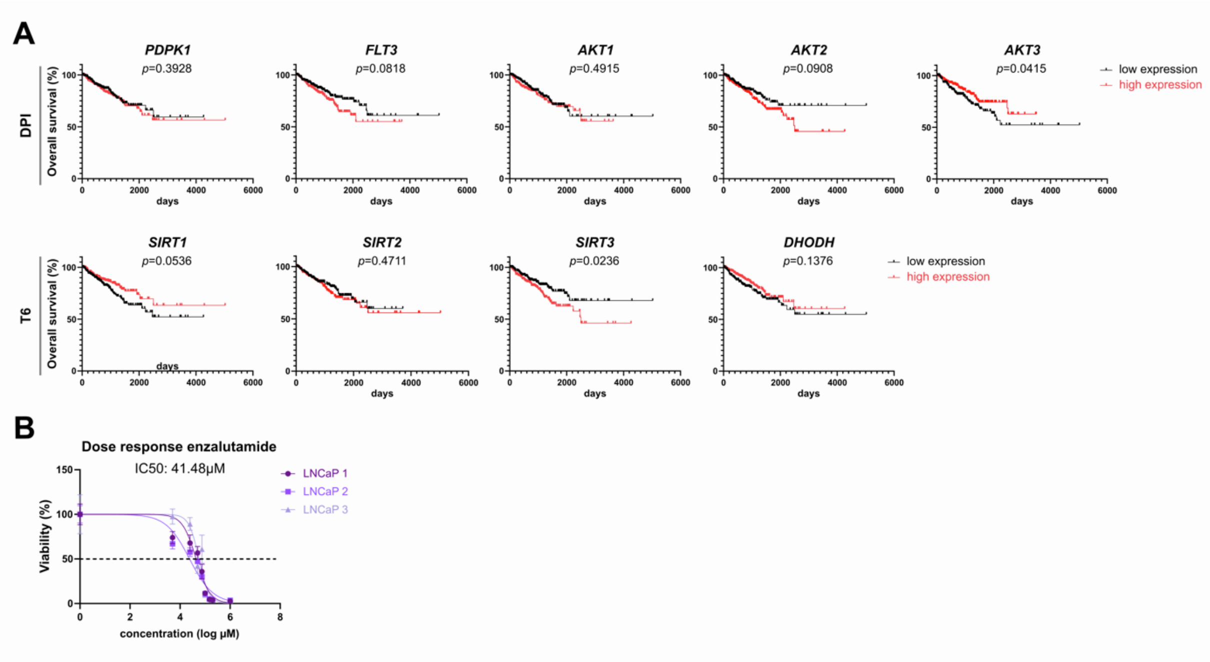

Figure S7. Kaplan-Meier analysis of DPI and T6 targets on publicly available human PCa data and response of LNCaP cells to enzalutamide, Related to Figure 7.

(A) Kaplan-Meier survival analysis based on TCGA-PRAD RNA sequencing data for DPI targets *PDPK1*, *AKT1*, *AKT2*, *AKT3*, and *FLT3* (top) and T6 targets *SIRT1*, *SIRT2*, *SIRT3*, and *DHODH* (bottom).[3] Statistical analysis was done using GraphPad Prism 8.0.2 (Mantel-Cox test). **(B)** Dose-response curves for enzalutamide on the human PCa cell line LNCaP. Points represent means and ± SD of technical triplicates (N=3). Curve fitting was performed using GraphPad Prism 8.0.2.

SUPPLEMENTARY TABLES

Table S1. PCR primer sequences, Related to STAR Methods.

**Genotyping primer**	**Sequence**
Cre fw	ATGCTTCTGTCCGTTTGCCG
Cre rev	TGAGTGAACGAACCTGGTCG
CreCut fw	CGACCAGGTTCGTTCACTCA
CreCut rev	GGTTGGCAGCTCTCATGTCT
Pten fw	TTCAGTTGTCTTTTACATTTCCTTTG
Pten rev 1	GTCAGAAACGGCCTTAACGA
Pten rev 2	TCAGGTAAGGGGTACTGATTTTT
Stat3 fw	GCGTCTGACTCTACA ACC
Stat3 rev 1	AGCCTCATCCTTAGGTACT
Stat3 rev 2	GACTGTGATAACCTTCAGTG
Tp53 fw	CACAAAAACAGGTTAAACCCAG
Tp53 rev 1	AGCACATAGGAGGCAGAGAC
Tp53 rev 2	GAAGACAGAAAAGGGGAGGG
**qRT-PCR primer**	**Sequence**
Pten fw	GACATTATGACACCGCCAAA
Pten rev	CAGTGAATTGCTGCAACATGA
Stat3 fw	AGACTCTGGGGATGTTGCTG
Stat3 rev	ACAGGCTGCCGTTGTTAGAC
Tp53 fw	CCATGGCCCCTGTCATCTTT
Tp53 rev	TGACCCACAACTGCACAGG
Ar fw	ATTGAGCCAGGAGTGGTGTG
Ar rev	ACCATCAGTCCCATCCAGGA
β-Actin fw	ATGCCCTGAGGCTCTTTTCC
β-Actin rev	AATGCCTGGGTACATGGTGG

Table S2. Antibody dilutions, Related to STAR Methods

**Target**	**Dilution**
β-ACTIN	WB 1:5000
AKT (pan)	WB 1:1000
CK8	IHC 1:200
KI67	IHC 1:400
P63	IHC 1:80
pospho-AKT	WB 1:1000
PTEN	WB 1:1000
STAT3	WB 1:1000
TP53	WB 1:500
AR (Abcam)	WB 1:1000<br>IHC 1:120
AR (Cell Signaling)	IHC 1:120

SUPPLEMENTARY REFERENCES

1. Goldman, M.J., Craft, B., Hastie, M., Repečka, K., McDade, F., Kamath, A., Banerjee, A., Luo, Y., Rogers, D., Brooks, A.N., et al. (2020). Visualizing and interpreting cancer genomics data via the Xena platform. Nat Biotechnol *38*, 675–678. https://doi.org/10.1038/s41587-020-0546-8.

2. Abeshouse, A., Ahn, J., Akbani, R., Ally, A., Amin, S., Andry, C.D., Annala, M., Aprikian, A., Armenia, J., Arora, A., et al. (2015). The Molecular Taxonomy of Primary Prostate Cancer. Cell *163*, 1011–1025. https://doi.org/10.1016/j.cell.2015.10.025.

3. Smith, J.C., and Sheltzer, J.M. (2022). Genome-wide identification and analysis of prognostic features in human cancers. Cell Reports *38*, 110569. https://doi.org/10.1016/j.celrep.2022.110569.
